# Exploring barriers to and opportunities for improving mental healthcare access for women caregivers living with HIV in Nigeria: A qualitative study

**DOI:** 10.1371/journal.pgph.0005834

**Published:** 2026-08-14

**Authors:** Ifeyinwa Lilian Ezenwosu, Ugochukwu Uzoechina Nwokoro, Elias Chikee Aniwada, Izuchukwu Frank Obi, Justus Uchenna Onu, Chidiebere Clifford Aniagboso, Eunice Enabulele, Chijioke Thaddeus Asogwa, Chika N. Onwasigwe

**Affiliations:** 1 Department of Community Medicine, University of Nigeria Teaching Hospital, Enugu, Enugu State, Nigeria; 2 Department of Community Medicine, Federal Teaching Hospital, Owerri, Imo State, Nigeria; 3 Department of Community Medicine, Faculty of Medical Sciences, College of Medicine, University of Nigeria Enugu Campus, Enugu, Enugu State, Nigeria; 4 Department of Mental Health, Nnamdi Azikiwe University, Awka, Anambra State, Nigeria; 5 Federal Neuropsychiatric Hospital, Enugu, Enugu State, Nigeria; 6 Department of Community Medicine, Enugu State University College of Medicine, Parklane, Enugu, Enugu State, Nigeria; University of Colorado Anschutz Medical Campus: University of Colorado - Anschutz Medical Campus, UNITED STATES OF AMERICA

## Abstract

Human immunodeficiency virus **(**HIV) has become a chronic condition due to improved outcomes with antiretroviral therapy (ART), resulting in increased mental health needs among people living with HIV. Women caregivers living with HIV, additionally burdened by caregiving stress, are at higher risk of mental health problems compared to their HIV-negative counterparts and the general population. In Nigeria, efforts to improve HIV-mental healthcare access largely prioritize key populations such as youth, with these caregivers remaining an underserved group. This study aimed to identify barriers to and opportunities for improving mental healthcare access for these caregivers in Nigeria. We conducted a qualitative phenomenological study involving four focus group discussions with these caregivers and 12 key informant interviews with frontline healthcare workers providing HIV services. We interviewed 52 participants (40 women caregivers and 12 healthcare workers). We analyzed transcripts using content analysis and identified themes using Levesque and colleagues’ framework for access to healthcare. This study was conducted and reported in accordance with the Consolidated Criteria for Reporting Qualitative Research (COREQ). Eight themes across five dimensions of healthcare accessibility at health system level and five corollary abilities at population level emerged. Barriers included unavailability of integrated mental healthcare-ART services, lack of training for healthcare workers on mental healthcare, stigma, lack of awareness and limited mental health literacy among the caregivers, long clinic waiting times, transportation costs and concerns for productivity losses while accessing care. Existing support groups and economic empowerment schemes emerged as opportunities for improving access to mental healthcare for these caregivers. Barriers to mental healthcare access for the women caregivers span across dimensions of healthcare accessibility at health system and patient levels, with implications for integrating mental healthcare with routine HIV services. Effectively integrating mental healthcare into routine HIV programmes requires systematically addressing these barriers while leveraging existing opportunities.

## Introduction

According to the World Health Organization (WHO) and the Joint United Nations Programme on HIV/AIDs (UNAIDS), an estimated 40.8 million people were living with HIV, and 1.3 million more people acquired HIV globally in 2024 [1,2]. Women and girls bear a substantial burden of the HIV pandemic [3], accounting for more than half of all people living with HIV globally in 2024 [2]. Furthermore, approximately 45% of all new people acquiring HIV were women and girls [2]. In sub-Saharan Africa, women and girls accounted for approximately 63% of all new people acquiring HIV [2].

Over the last two decades, significant gains have been recorded in the global effort to end the HIV pandemic. The gains made in the treatment and management of HIV, specifically the use of antiretroviral therapy (ART), have resulted in significant improvements in life expectancy for people living with HIV globally and particularly among those in the developing regions of the world [4,5]. With these advancements, HIV is no longer considered terminal; rather, it is a manageable condition that permits long-term survival and healthy ageing. However, this has also resulted in an increasing psychosocial and mental health need among people living with HIV, requiring greater clinical and public health attention [6].

Consequently, the prevalence of mental health disorders remains high among people living with HIV, with 28% to 62% of people living with HIV reported to have mental health symptoms [7]. Among people living with HIV, the prevalence of common mental health disorders such as depression is significantly higher in women compared to men [8–10]. Vulnerable groups such as women caregivers living with HIV are particularly at increased risk of mental health disorders compared to their HIV-negative counterparts and the general population [7]. Common mental health disorders reported among these caregivers include depression, anxiety and trauma-related mental health illness [11]. Despite women accounting for a substantial proportion of people living with HIV and the number of new people acquiring HIV, they remain under-represented in the efforts to improve mental healthcare access [11]. Furthermore, these caregivers, additionally burdened by caregiving stress, are at even higher risk of mental health disorders [12] but may face peculiar challenges with accessing the needed mental healthcare services. A recent study found that of a cohort of 1422 women living with HIV, 51.1% lacked access to mental health services either because the services were not available, they were unaware of the availability of the services, or they were not engaged in antiretroviral therapy (ART) care [13]. In high-income settings, multi-level factors such as system-level barriers, like lack of resources and lack of mental health support; provider-level barriers such as limited clinic space and time constraints; and individual-level barriers such as socio-economic conditions and stigma have been identified as important barriers limiting access to psychosocial care for people living with HIV [14]. On the contrary, multidisciplinary and dedicated support for mental health challenges, as well as peer support, have been found to facilitate access to mental healthcare [14]. In low-income settings, similar system, provider and individual-level barriers and facilitators of mental healthcare access for people living with HIV have been reported [15,16]. The World Health Organization (WHO) advocates for the integration of mental health care into HIV programmes, as this improves access to mental health care for people living with HIV and promotes the delivery of cost-effective mental health interventions to those who need them [17]. National guidelines for the treatment and management of HIV in many Low- and Middle-Income Countries (LMICs), including Nigeria, recommend routine screening for common mental health disorders such as depression and anxiety among people living with HIV [18,19]. This is in recognition of the rising need to integrate mental healthcare services into routine HIV services. However, evidence shows that routine screening for common mental health disorders among people living with HIV is not regularly done by healthcare workers [20]. Additionally, in Nigeria, efforts to improve mental healthcare access for people living with HIV have largely focused on key populations such as adolescents and young people because of emerging mental health challenges that they face as they transition from childhood to adolescence and young adulthood and also due to the high tendency to disengage from HIV care. Mental healthcare services for other key populations, such as men who have sex with men, female sex workers and people who inject drugs, as well as the general population of people living with HIV, are scanty. Furthermore, limited attention has been given to women caregivers living with HIV, despite the high burden of common mental health disorders among these caregivers and the peculiar challenges they face in accessing the needed mental healthcare. In Nigeria, as in many LMICs, there is a paucity of empirical data on the barriers to the provision of and access to mental healthcare services for people living with HIV, including women living with HIV [11]. Addressing the mental well-being of people living with HIV requires an understanding of the context-specific factors constraining access to mental healthcare, especially for vulnerable groups such as women caregivers living with HIV. This study aimed to identify the barriers to and opportunities for improving access to mental health care for people living with HIV from the perspectives of frontline healthcare workers providing clinical care to people living with HIV and women caregivers living with HIV receiving antiretroviral therapy (ART).

## Materials and methods

### Study area and setting

This study was conducted in Enugu State, one of the five states in the South-East geopolitical region of Nigeria. The state has a projected population of 4.4 million people with an annual growth rate of 3.0% [21]. The state is predominantly rural, and a substantial proportion of the working population is engaged in farming, although traders and civil servants are notable in number. The state has an estimated HIV prevalence of 2.0% among adults aged 15–64 years, based on the National HIV/AIDS Indicator and Impact Survey, a national population-based survey conducted in 2018 [22]. A more recent modelling study estimated the prevalence of HIV to be 1.3% among adults aged 15–49 years in 2022 [23]. The study setting comprised antiretroviral therapy (ART) clinics at two large government-owned tertiary health facilities, the University of Nigeria Teaching Hospital (UNTH) and Enugu State University of Science and Technology Teaching Hospital (ESUTH). The ART clinics in both health facilities provide comprehensive HIV care services for people living with HIV. Each of these two facilities provides HIV care services to approximately 150 people living with HIV every week. They serve as referral centres for the secondary and primary health care facilities within the state and from neighbouring states in the South East region of the country.

### Study design

This was a qualitative phenomenological study involving the use of focus group discussions (FGDs) to explore barriers to and opportunities for improving access to mental health care for women caregivers living with HIV accessing ART services at the selected health facilities and key informant interviews (KIIs) used to obtain information from healthcare workers providing clinical care to people living with HIV on their experiences with mental health care availability and access for these caregivers at the study sites.

#### Design and analytical framework.

**Fig 1:**
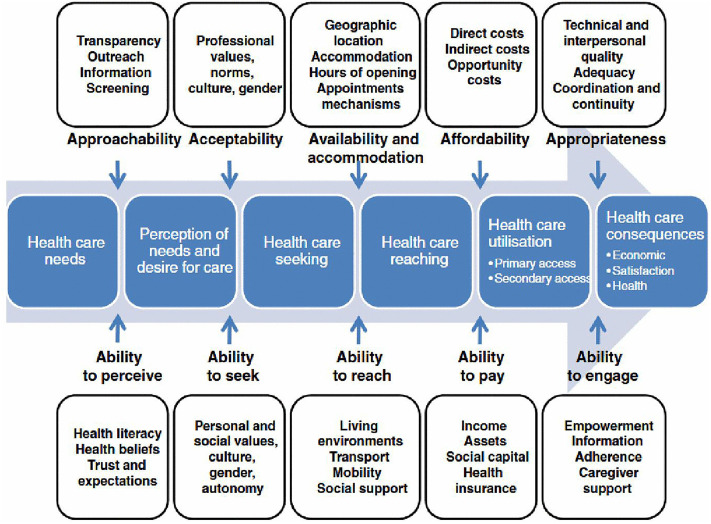
Conceptual framework for access to health care by Levesque et al. [24] Adopted from Levesque et al. [24].

The design and analytical framework for this study were informed by Levesque and colleagues’ conceptual framework for access to healthcare [24]. Levesque and colleagues’ conceptual framework (Fig 1) has been widely used by researchers to explore access to various healthcare services and in diverse settings. The framework allows researchers to comprehensively assess the complex and dynamic process of access, both at the health system and population levels [25,26]. The framework conceptualizes access to health care across five dimensions (i.e., approachability, acceptability, availability and accommodation, affordability, and appropriateness), along with five corollary abilities of populations (ability to perceive, ability to seek, ability to reach, ability to pay, and ability to engage) [24]. These concepts, originally conceptualized by Levesque and colleagues [24], are summarized by Schwartz and colleagues [26] as presented in Table 1.

**Table 1:** Summary of dimensions of barriers to health care access by Levesque and colleagues.

Dimension	Description	Population-level corollary	Context in mental health care access
Approachability	Approachability of health services refers to the provider’s efforts to make patients more aware of their services and is often related to transparency, outreach, provision of information and education about services to patients	Barriers to perceiving need	Health literacy, as well as existing cultural beliefs about mental health and mental illness, may pose a barrier to the ability to perceive the need for mental health care
Acceptability	Seeking health care relates to cultural and social factors affecting services. It examines whether people will accept services that do not conflict with their personal, social, or cultural values so that they can access them without feeling unsafe or uncomfortable	Barriers to seeking care	Stigmatization and discrimination, often associated with mental health illnesses in some cultural settings, may pose a barrier to the ability to seek mental health care by people living with HIV
Availability and accommodation	This dimension includes patients’ ability to physically access services based on factors such as personal mobility (e.g., the presence of disability, access to transportation) and external circumstances (e.g., occupational flexibility)	Barriers to reaching health care	In the context of mental health care, the availability of services also refers to the general presence of mental health services and health care providers needed, as well as their accessibility to patients
Affordability	Affordability refers to patients’ economic capacity to spend resources and time in order to use the required services (e.g., direct costs, secondary costs (for getting to an appointment), and opportunity costs (for going on sick leave from work). The ability to pay for health care refers to patients’ economic capacity to pay for actual healthcare services	Barriers to utilizing health care and barriers to the ability to pay	Although certain aspects of HIV services, like antiretroviral medications, are provided free by the government in Nigeria, accessing mental healthcare services in addition to ART care may imply making additional payments for medications relating to the mental illness for which care is being sought
Appropriateness	Appropriateness of care is reflected in the fit between treatment and patients’ needs, its timeliness and coordination, and its quality	Barriers to the ability to engage	People living with HIV may be unwilling to engage with the health system to access mental healthcare services if that means waiting longer to consult with the healthcare personnel responsible for providing needed health services

ART = antiretroviral therapy, HIV = Human immunodeficiency virus.

### Recruitment of study participants

#### Focus Group Discussions (FGDs).

A total of four FGDs were conducted among women caregivers living with HIV, consisting of 10 FGD participants per group in the two study sites (two per site). Caregivers, aged 18 years and above, were identified and recruited to participate in the interview on the day of ART clinic attendance using purposive sampling. A caregiver was defined as a participant who provides unpaid physical support, such as helping in activities of daily living, shopping, food preparation, helping in administering medication, overseeing medical appointments, and providing financial and emotional support to the child/children or other family members [27]. In each study site, two separate FGDs were conducted on different days, with eligible participants in each FGD comprising a mix of younger women caregivers aged 18–24 years and older women caregivers aged 25 years and above.

#### Key Informant Interviews (KIIs).

A total of 12 KIIs were conducted with selected healthcare workers at the two study sites (six healthcare workers providing HIV care services per site). Key informants in each study site comprised two clinicians, two clinical nurses and two counsellors with more than one year of experience providing clinical care to people living with HIV. Eligible key informants in each study site were identified by the research team at the ART clinics, approached face-to-face, and purposively recruited to participate in the interviews. The use of a purposive sampling technique ensures that participants with relevant lived or personal experiences and those with sufficient knowledge in the subject being researched are identified and selected to participate in a qualitative study.

### Data collection

Data were collected through qualitative interviews utilizing FGDs and KIIs conducted between 1^st^ July and 30^th^ September 2024. Three of the researchers and two trained research assistants conducted the interviews with selected eligible participants in secure, convenient locations within the study sites, prioritizing participant privacy and research integrity. Two separate but complementary semi-structured interview guides, an FGD guide (S1 File) and a KII guide (S2 File), were developed by the researchers through literature review and consultation with experts. The FGD guide was used to obtain information from the caregivers regarding their personal experiences with mental health problems following their diagnosis with HIV, health-seeking behaviour for mental health problems, as well as their perceived barriers to accessing mental health care and existing opportunities for improving access to mental health care services. A five-item KII guide was used to obtain information from selected frontline healthcare workers on their experiences with recognizing and managing mental health disorders among people living with HIV, availability of facility-based support programmes for prevention and management of mental health disorders among people living with HIV, perceived usefulness of the support programmes to women caregivers living with HIV, barriers to access and opportunities for improving access to mental health care for these caregivers. In total, 16 interviews were conducted (12 KIIs and 4 FGDs). Interviews and FGDs were conducted in English and recorded using a digital recorder with the consent of the study participants. All interviews were conducted face-to-face, and each lasted approximately 26–48 minutes. Data saturation was assessed iteratively through concurrent data collection and reflexive thematic analysis. Saturation was achieved when no new themes emerged. The research team used a two-phase approach across Key Informant Interviews (KIIs) and Focus Group Discussions (FGDs) to ensure rigour. After an initial round of ten KIIs and three FGDs established a codebook, two additional KIIs and one FGD were conducted to confirm that no new codes or themes were identified.

### Data management and analysis

Audio recordings from the interviews were transcribed verbatim, and transcripts were stored in a secure, password-protected computer. To ensure analytical depth and researcher immersion, all qualitative data collected during this study were analyzed manually without the use of computer-assisted qualitative data analysis software (CAQDAS). Analysis rigorously followed the inductive thematic analysis framework: data familiarization, generating initial codes, searching for themes, reviewing themes, defining and naming the themes and ensuring trustworthiness. To ensure trustworthiness, this study adhered to the four criteria of qualitative inquiry: Credibility was established through methodological triangulation of FGD and KII findings; transferability was supported by rich descriptions of the sampling approach, study setting, and participant demographics; dependability was maintained through a traceable coding framework and theme identification process; and confirmability was achieved through continuous researcher reflexivity to minimize bias. During the thematic analysis process, transcripts were coded line-by-line using a structured codebook. This codebook was continuously refined to reflect emerging themes and is available as Supplementary File 3 (S3 File). Transcripts were carefully read and reread by the lead investigator, and content analysis was deployed to analyze transcripts from the FGDs and KIIs. Content analysis is a frequently used approach for analyzing qualitative data and involves systematically coding large amounts of qualitative data to explore patterns of words used and how they relate to each other in the dataset [28]. Directed qualitative content analysis was employed to identify themes or categories informed by Levesque and colleagues’ conceptual framework for access to health care [24]. In this study, themes were generated by categorizing texts within each transcript and using these labels to identify and code similar text both within and across transcripts. The specific steps taken in identifying and coding texts included, firstly, repeatedly reading the texts to obtain a comprehensive overview. Secondly, highlighting key elements in the texts that relate to or align with a theme. Thirdly, rereading KII and FGD transcript texts and labelling themes based on the main areas (Approachability, Availability and accommodation, Acceptability, Affordability and Appropriateness). Fourthly, identifying subareas relating to the population-level corollary according to Levesque and colleagues’ conceptual framework and lastly, assigning each subarea as a barrier or opportunity based on its influence on mental healthcare access. Coding continued until no new concepts emerged from the data. Coding consistency was checked by one of the co-investigators to ensure trustworthiness, and theme confirmation was reached after discussion among the lead investigators and two co-investigators. The most common responses in each thematic area were presented as quotes. To be reflexive, three authors (some of whom are working in the study sites) involved with the analysis tried to be self-aware and transparent about how their roles, perspectives, and experiences may have influenced the analysis and interpretation. Key approaches adopted by the authors towards ensuring reflexivity included the use of reflexive notes to document their thoughts and reactions to the data generated and holding regular collaborative discussions with research supervisors, including the lead researcher, to ensure interpretation of the themes reflected the study participants’ perspectives. This qualitative study was conducted and reported in accordance with the Consolidated Criteria for Reporting Qualitative Research (COREQ) [29].

### Ethics statement

Ethical approval for this study was obtained from the Health Research Ethics Committee of the University of Nigeria Teaching Hospital, Ituku-Ozalla, Enugu (UNTH/HREC/2024/04/939). Permission to conduct the study was obtained from the clinical lead of each study facility. Written informed consent was obtained from all study participants after providing detailed information on the study objective, procedure, and anticipated risks and benefits. Confidentiality of all study participants was ensured and maintained during and after the study by ensuring the anonymity of study participants.

## Results

A total of 40 women caregivers living with HIV and 12 HCWs participated in the FGDs and KIIs, respectively, from the two study sites. Of the 40 FGD participants, the majority were aged 25 years and above, and most had been on ART care for five years or more (Table 2). Among the 12 KII participants, seven were females, and the majority had provided ART care to people living with HIV for five years or more (Table 3).

**Table 2:** Characteristics of focus group discussion participants.

Age	Duration on ART care		Total
	< 5 years	≥ 5 years	
18 – 24 years	15	2	17
≥ 25 years	0	23	23
**Total**	15	25	40

**Table 3:** Characteristics of key informant interview participants (N = 12).

Age	Sex	
	Male	Female
25 – 34 years	3	1
35 – 44 years	1	3
45 – 54 years	1	3
**Years of experience providing ART care**		
1 – 4 years	1	1
5 – 9 years	2	3
≥ 10 years	2	3

Analysis of interviews with frontline healthcare workers and the women caregivers revealed barriers to and opportunities for improving access to mental healthcare services across the five dimensions of healthcare accessibility at the health system level and five corollary abilities at the population level, which interact with the dimensions to pose as barriers and opportunities for improving access (Table 4).

**Table 4:** Summary of barriers to and opportunities for improving access to mental healthcare services by women caregivers living with HIV.

	Dimension	Population-level corollary	Theme
**Barriers**			
1	Availability and accommodation	Ability to seek	Unavailability of formal mental health care services integrated with HIV services at ART clinics
			Lack of training of healthcare workers providing ART services to recognize mental health challenges and integrate mental health care into routine HIV care
2	Approachability	Ability to perceive need	Lack of awareness among people living with HIV and women caregivers living with HIV about mental illness
			Limited mental health literacy among people living with HIV and women caregivers living with HIV
3	Acceptability	Ability to seek	Stigma
4	Appropriateness	Ability to engage	Long clinic waiting time
5	Affordability	Ability to reach/Ability to pay	Transportation costs
			Productivity losses
**Opportunities**			
1	Availability and accommodation	Ability to seek	Existing support groups
			Economic empowerment schemes

### Barriers

#### Availability and accommodation: Ability to seek.

**Unavailability of mental healthcare services at ART clinics:** Multiple healthcare workers highlighted the unavailability of mental healthcare services within the ART clinic as a major barrier to access when needed by women caregivers living with HIV. Many of the healthcare workers reported that mental healthcare services for people living with HIV were either not available or integrated with ART services at the clinic; or that they were unaware of the availability of mental healthcare services for people living with HIV tailored to the needs of women caregivers living with HIV in the clinic.

*Right now, right now, right now, not just no, right now, absolutely no, I’m not aware – P1 (Clinician)*

*I have not, I am not sure of that one [availability of mental healthcare service or programme] – P12 (Counsellor)*

*There’s no special [mental health] programme. The only thing I know is that we have like their support group meetings that help them, but not specifically to mental health – P7 (Clinician)*

Similarly, multiple participants among the women caregivers highlighted the unavailability of mental healthcare services as a significant barrier impacting access to mental healthcare services.

*What I want to say is that, you people should try and get a [mental health] department here…Please, it will be better for all of us if mental health became a department here… - P14 (Woman caregiver)*

*No, I have not heard about that one [mental healthcare service availability]… - P20 (Woman caregiver)*

*I don’t know of any [mental healthcare] programme. - P27 (Woman caregiver)*

Other healthcare workers reported that cases of mental health disorders among these caregivers are referred to the psychiatrists at another clinic outside the ART clinic. However, there is no way of knowing if the patients actually access the mental healthcare that they need there due to the lack of a two-way referral system in the health facility.

*I don’t know but all I know is that if we identify such patients, we usually refer the person to go and see the doctor, our clinic, the clinician here. So, doctor will now assess and refer the person to see a psychiatrist. You know [this health facility] has psychiatric unit… - P10 (Nurse)*

*In fact, I referred one to psychological clinic…and she never came back… that’s the problem, you refer there, you do not get [feed]back, so you don’t know what happens… - P1 (Clinician)*

**Lack of training for healthcare workers to integrate mental healthcare services with ART services:** Another critical barrier to access to mental healthcare services for these caregivers identified by healthcare workers is the lack of training for frontline healthcare workers to recognize and screen for mental health disorders among people living with HIV.

*One of the barriers I see is [lack of] training. We need to be trained. So that you may… pick the signs at a very early stage. Because if you are not trained, you may not pick it... so training is important… - P7 (Clinician)*

#### Approachability: Ability to perceive the need for mental healthcare services.

**Lack of awareness and limited mental health literacy**: One healthcare worker identified lack of awareness, limited mental health literacy, poor knowledge about mental health and beliefs related to mental health disorders to be important barriers to access to mental healthcare services by the women caregivers. Hence, limiting the ability to perceive the need for mental healthcare services by these caregivers.

*Barriers will be maybe ignorance. If they don’t know that uhm there is uhm things like that [and] maybe care or resources that will solve their problem, they will not access the care... So, ignorance, then the provision of that care and then acceptance. If they will equally accept it okay somebody has mental health issues and you refer the person to psychological unit [but]…if the person doesn’t accept that he is even having a problem, the person will not go… - P4 (Nurse)*

#### Acceptability: Ability to seek mental healthcare services.

**Stigmatization**: Some participants identified fear of stigmatization and discrimination as significant barriers to access to mental healthcare services, even when made available at the ART clinic. This poses a particular challenge to the ability to seek mental healthcare services alongside ART services by women caregivers living with HIV

*Then most of them too, we found out that most of them from the statistics, most of them come from very far areas…They want somewhere far from where they stay because of the issue of stigmatization and so it’s also […] a barrier… - P8 (Clinician)*

*…There are some people that are shy. They wouldn’t want to run into people that know them… - P35 (Woman caregiver)*

#### Appropriateness: Ability to engage mental healthcare services.

**Long clinic waiting time**: An important determinant of access to healthcare services is the appropriateness of the health services provided in terms of whether the health services meet clients’ needs, are provided in a timely manner, and the amount of care taken by healthcare personnel in assessing health problems and determining the appropriate course of treatment. One healthcare worker acknowledged that long clinic waiting times may discourage women caregivers living with HIV from seeking or engaging additional care for mental challenges they may be facing at the health facility, impacting their ability to engage mental healthcare services they may need.

*…the waiting time in the clinic is a barrier, so we try to shorten the waiting time but if it’s not shortened it will affect them, they will just pick their medication and go home, neglecting this mental health […] issue and it keeps aggravating—you know mental health once you don’t treat it immediately, it keeps aggravating [and] it keeps getting worse and worse, and it turns into another thing. Somebody that has anxiety today may come down with depression tomorrow and start talking about suicide the next time… - P4 (Nurse)*

Some women caregivers highlighted that the processes of obtaining care can be tedious, leading to long waiting times at the clinic.

*One thing I want to talk about is time. Some of us are public servants and civil servants. Like me, I told my [supervisor that] I want to rush somewhere and come back and I have been here since eight o’clock. If you can help us, […] adjust the time to favor us in our works… - P18 (Woman caregiver)*

*If you come early, you are meant to go early whether you work in the office or you sell goods. But we will come here, and the nurses and doctors waste our time. – P13 (Woman caregiver)*

#### Affordability: Ability to reach and ability to pay for mental healthcare services.

**Transportation costs and productivity losses**: Participants raised concerns regarding the economic cost in terms of direct and indirect costs that patients encounter to be able to access mental healthcare services. For example, distance and transportation costs were reported by frontline healthcare workers as barriers to accessing mental healthcare services at the health facility. This impacts the ability to reach and the ability to pay for mental healthcare services by these caregivers.

*Hmm, some will tell you that we are coming from a very far place…that they won’t be making it because of transportation…- P11 (Counsellor)*

*For me the challenge that I observed is challenge of transportation fare… - P10 (Nurse)*

*The problem, what they usually complain, most of them when they miss appointment and you call them, it is usually transport fare that is their problem. Usually, that’s why they miss their appointments… - P8 (Clinician)*

Additionally, potential productivity losses due to the length of time spent in the clinic when accessing mental healthcare services, where available, were highlighted by one healthcare worker as a barrier to accessing mental healthcare services by the women caregivers.

*Most of them having to leave […] what they are doing; they cherish it so much. Some of them are farmers, and it could be time of harvesting so it’s going to be a barrier. Whatever time of the year, whether it’s rainy season, they are in their farms, dry seasons they are harvesting… - P8 (Clinician)*

Some caregivers acknowledged the barrier posed by the high cost of transportation to their ability to reach the health facility and their ability to attend support group meetings if available at the health facility. In addition to transportation costs, other indirect costs, such as concerns about the timing of provision of services conflicting with other economic activities, such as farming or family engagements, were identified as barriers to access to mental healthcare services

*The second one could be that the person doesn’t have transport money to attend or come for the meeting. Like me, I do well in the support group. I attend with my child but I haven’t been able to attend this year because I relocated […]to a farther place… - P35 (Woman caregiver)*

*There are some people that get prepared to attend but won’t have transport money while some may not have chance to attend. There was a woman I reminded about the meeting, but she said she wouldn’t have time because she was going to the market to buy foodstuff for her family… - P37 (Woman caregiver)*

### Opportunities

#### Availability and accommodation: Ability to seek.

**Support groups**: Multiple healthcare workers highlighted the role support groups play in providing an opportunity for the women caregivers to obtain emotional support from peers.

*…support group, when you’re coming out, the thing is they say that [an] idle mind is the devil’s workshop. I believe that if you’re coming out, you’ll be learning so many things…when you come there [support group meetings], you’ll see people with sickness but they are happy, because they are meeting […] people that can teach them, people that can talk to them, make them to ease their pains. Yes, so I believe that, that social […] support group helps a lot… - P12 (Counselor)*

*…there are groups that help. There is the Orphans and Vulnerable Children [support group], apart from the support group, especially mothers maybe that have children that are positive so they help them… - P7 (Clinician)*

Similarly, one of the women caregivers reported that although there are no formal mental healthcare services available at the ART clinics, support groups serve as the most common form of mental health care available to them. The availability of support groups and attending meetings was reported as an important facilitator to mental healthcare access, mainly through social engagements with peers and community-based organizations that sponsor the support group meetings.

*There’s a meeting that holds every Saturday in a month. When you come to the meeting, they address things like this. Instead of overthinking, the things they talk about in the meeting, helps to encourage us [...] We also get to meet people like us which is comforting… - P38 (Woman caregiver)*

**Economic empowerment schemes**: One healthcare worker highlighted the role economic empowerment schemes, such as village savings and loan schemes, play in not only providing economic support as their primary outcome, but also psychosocial benefits as a secondary outcome.

*…I know that Village Loan and Savings Association, has been going on here…so you’ll be saving, no matter how little it’s something, at the end of the cycle when they will share it, you carry your own savings. Ehen, then it will help you, even your school eh, your children’s school fees, it will help you, their clothing, food, so many of them, so many of them that you can use your savings to achieve… P11 (Counsellor)*

## Discussion

This study identified the barriers to and opportunities for improving access to mental healthcare services for people living with HIV from the unique perspectives of frontline healthcare workers providing clinical care to people living with HIV and women caregivers living with HIV receiving antiretroviral therapy (ART). These caregivers are a population that is especially prone to inequalities and inequity of access to mental healthcare services, and were previously under-researched. This study identified eight broad themes spanning across the five dimensions of healthcare accessibility at the health system level and five corresponding abilities at the population level, which interact with the dimensions to pose as barriers and opportunities for improving access to mental healthcare for these caregivers.

There were six themes relating to barriers to accessing mental healthcare services (unavailability of mental healthcare services integrated with HIV services, lack of training for healthcare workers on mental healthcare, lack of awareness/limited mental health literacy, stigmatization, long clinic waiting time and transportation costs/productivity losses). According to the Levesque framework, the lack of integrated ART-mental healthcare and trained staff corresponds to the availability and accommodation dimension. Here, availability refers to the physical presence of health resources, including facilities, qualified personnel, and the operational capacity to provide care, amongst other considerations. In this study, participants highlighted that formal mental healthcare services were unavailable at ART clinics. This is a critical finding because it highlights the persistent gaps in optimizing patient-centered care for people living with HIV, especially with regard to prioritizing their mental health needs, which impacts their mental well-being and health-related quality of life. This poses a barrier to the ability to seek mental healthcare services by these caregivers at the patient or population level. Lack of resources within ART clinics has been reported to be a key barrier to access to psychosocial and mental health care for people living with HIV [14]. Studies have documented that to achieve long-term success and optimal quality of life for people living with HIV, there is a need to ensure the integration of ART services and mental healthcare services [30]. Furthermore, the existence of opportunities for the integration of mental health into HIV programs in LMICs has been demonstrated by previous studies [31]; however, this is seldom done [20]. In this study, the lack of training of frontline healthcare workers providing routine ART services on mental health care was reported as a barrier to accessing mental healthcare services by women caregivers living with HIV. This has implications for integrating mental health care with routine ART services by HCWs, which has been shown to be effective in achieving viral suppression and reducing depression and other mental health disorders, such as harmful substance use among people living with HIV [32]. In Nigeria, national HIV prevention and treatment guidelines recommend that people living with HIV be routinely screened for mental health disorders such as depression [18,19], but studies have shown that this is rarely done by healthcare workers, reflecting potential gaps in capacity. The reason for this gap between policy and practice may be that the training for these healthcare workers focuses only on building their capacity to provide HIV care, with little or no attention paid to building their capacity to simultaneously screen for and provide mental healthcare to those who need it while accessing their routine HIV care. Lack of training or inadequate capacity of healthcare workers providing ART services to screen, identify and treat common mental health disorders has been highlighted as an important barrier to integrating mental health care services with routine ART care for people living with HIV by previous studies [31,33].

Participants in this study reported fear of stigmatization as a barrier to accessing mental healthcare services by women caregivers living with HIV. This is noteworthy because women are more likely to experience stigma [34] and particularly important because of the negative social, cultural and spiritual beliefs associated with not only HIV but also mental illnesses within the community and by healthcare personnel, resulting in multiple stigma - HIV-related stigma and mental illness-related stigma. This may lead the caregivers to disregard symptoms of mental illness or be unwilling to accept mental health services if available. The negative impact that stigma associated with mental illness has on the health-seeking behaviour of people living with HIV has been previously documented [15]. Furthermore, stigma has been associated with an increased likelihood of being diagnosed with other mental health disorders, such as anxiety and depression, especially in the face of social isolation [35]. Stigma has been shown to be a barrier to accessing healthcare services for people living with HIV [34]. The impact of this as a barrier to mental healthcare access is heightened for individuals who have experienced Adverse Childhood Experiences (ACEs) and especially for women and girl children living with HIV who have a background of Adverse Childhood Experiences (ACEs) [36]**.** Because acceptability of healthcare services, including mental healthcare services integrated with routine ART services for people living with HIV, is dependent on cultural and social factors which dictate whether people accept the services being offered, it is important that interventions for improving access to mental healthcare services for people living with HIV holistically address stigma at the individual, community and health system levels.

Long clinic waiting time was reported by participants to be a barrier impacting the ability to seek mental healthcare services by the women caregivers in this study. Timeliness of provision of health services is a critical element in the appropriateness dimension in Levesque and colleagues’ framework [24]. Hence, clients may be unwilling to access mental healthcare services if that means that they would have to wait longer to engage with the healthcare personnel responsible for providing the health service needed. Longer waiting times have been shown by other researchers to be a barrier to accessing healthcare for people with chronic conditions, including mental illnesses [26].

As highlighted by some healthcare workers interviewed in this study, lack of awareness and limited mental health literacy among the women caregivers play a vital role in the ability to perceive the need for mental health services and thus pose a barrier to accessing mental healthcare services, even when available at the health facility [33]. This population-level element relates to the health system dimension of approachability in Levesque and colleagues’ framework [24]. Improved mental health literacy among people living with HIV can result in recognizing mental health challenges earlier, reduced stigma associated with mental illness, improved help-seeking behaviour, and enhanced access to mental healthcare services when provided for people living with HIV, including women caregivers living with HIV. The positive relationship between mental health literacy and improved health-seeking behaviour for mental health disorders has been previously demonstrated [37].

Direct costs, such as transportation costs, and indirect costs, such as productivity losses, were reported by participants as significant barriers impacting the ability to reach and the ability to pay for mental healthcare services by these caregivers. These relate to the affordability dimension of Levesque and colleagues’ framework for access to health care [24]. Direct costs incurred from consultation fees, transportation and medications and indirect costs incurred as a result of time spent seeking mental health care can pose significant barriers to accessing mental healthcare services, especially in poor resource settings and in the absence of optimal health insurance coverage. The inability to access and utilize mental healthcare services when needed due to the economic burden may result in missed diagnoses with attendant deterioration of mental health disorders, which may in turn negatively impact adherence to HIV medications, viral load suppression and clinic appointments with poorer health outcomes. Studies have demonstrated the high economic burden faced by primary caregivers of people living with HIV when seeking health care [38], and this burden is even more significant among people living with HIV who are being managed with other co-morbidities, including mental illness [39].

Regarding opportunities for improving access to mental healthcare services for women caregivers living with HIV, two broad themes (existing support groups and economic empowerment schemes) were identified. These fall within the availability and accommodation dimension and the corresponding ability to seek corollary of the Levesque and colleagues’ framework [24]. These findings suggest that there are existing opportunities for improving access to mental healthcare services for these caregivers, despite the current barriers. Some healthcare workers described the role economic empowerment schemes, such as village savings and loan schemes, play in providing economic support as their primary objective, but also psychosocial benefits as a secondary outcome. Economic empowerment can potentially help these caregivers to address the issue of transportation costs and other direct costs associated with clinic attendance, thereby improving their ability to seek and access available mental healthcare services alongside their ART clinic consultations and medications. Studies have demonstrated that enhancing social support for women affected by HIV in low-income settings through economic empowerment is feasible [40] and that economic empowerment of young girls can improve mental health and wellbeing by reducing mental health symptoms such as depressive symptoms [41]. Likewise, support groups could be leveraged to strengthen social support, create awareness and improve mental health literacy among people living with HIV, address stigma and foster a sense of community among these caregivers. Studies have shown that peer support groups improve clinical outcomes, retention in care, mental well-being and quality of life of people living with HIV [42,43]. As a result of these clinical, psychosocial and economic benefits of peer support for people living with HIV, national standards in high-income countries have evolved with recommendations that everyone living with HIV should have access to peer support [44]. Existing support groups present unique opportunities for improving access to mental healthcare services for people living with HIV, including women caregivers living with HIV, by providing veritable platforms for integrating mental healthcare services with routine ART services in LMICs [31].

### Strengths and limitations of study

An important strength of this study is the use of a broad mix of participants, including healthcare providers comprising clinicians, specialist nurses and counsellors with a vast range of experiences, roles and educational qualifications, as well as patients seeking care. This heterogeneous sample ensured that a comprehensive and multi-dimensional perspective was obtained from all stakeholders in the exploration of the barriers to and opportunities for improving access to mental healthcare for a particularly vulnerable group, often disproportionately affected by inequalities and inequities in HIV and mental health care service provision. Obtaining patients’ perspectives directly rather than by proxy provided additional insights into their unique experiences of access to mental healthcare services.

However, our study is not without limitations. As in other group participation-based research, FGD participants’ opinions and ideas may have been influenced by others in the group. To address this, we conducted multiple FGDs in each study site, utilizing groups consisting of a mix of younger and older women caregivers to obtain robust and diverse experiences and perspectives. In addition, we ensured that each member contributed adequately to the group discussions. This enabled the elicitation of as many varied experiences from participants without let or hindrance. Furthermore, because data collection, collation, and analyses were conducted by the authors, this may inadvertently lead to the infusion of the authors’ perspectives in interpreting the data. To address this, co-authors independently reviewed transcripts to verify and confirm themes, and the varying backgrounds and experiences of co-authors ensured that perspectives in the analysis and interpretation of the results are balanced. Lastly, although some who took part in conducting the research are living with HIV, this has not been documented here, owing to ongoing stigma and discrimination that can arise. Despite their involvement as research assistants and peer researcher, the limited inclusion of women caregivers living with HIV in the design, implementation and analysis stage of this study may have limited the ability of the study to explore certain key areas facing participants. For example, fear of violence, including violence against women and girls (VAWG) at the family, community and health facility levels, could play a critical role in hindering mental healthcare access for these caregivers. Incorporating women caregivers living with HIV in co-creating future research in this area would provide a more holistic understanding of the social and structural factors shaping mental healthcare access for this important key sub-population.

## Conclusions

Barriers to access to mental healthcare services for women caregivers living with HIV span across dimensions of healthcare accessibility at the health system and patient levels, which have implications for integrating mental health care service delivery with routine HIV services. Findings from this study reveal that despite the critical and multi-level barriers to mental healthcare access, existing support groups and women economic empowerment schemes present opportunities for improving access to mental healthcare services. While there is a clear need to accelerate efforts towards integrating mental healthcare with routine HIV services, this study suggests that for such efforts to be effective, context-specific barriers faced by vulnerable groups, such as women caregivers living with HIV, must be systematically addressed while leveraging existing opportunities to advance and scale mental health interventions, especially in poor resource settings. To address systemic failures such as the absence of a functional two-way referral system between ART clinics and specialized mental health clinics, implementing digital health solutions and referral trackers is highly recommended. These tools help bridge communication gaps, track patient linkage to mental health specialists, monitor treatment continuity, and enhance care coordination.

## Supporting information

S1 File Focus group discussion guide. (PDF)

S2 File Health care worker key informant interview guide. (PDF)

S3 File Themes, categories, subthemes, codes, descriptions and quotations. (PDF)

S1 Data Key informant interview and FGD transcripts. (PDF)
