# Supplementary material for: Exploring barriers to and opportunities for improving mental healthcare access for women caregivers living with HIV in Nigeria: A qualitative study
Source: PLOS Glob Public Health. 2026 Aug 14;6(8):e0005834. doi: 10.1371/journal.pgph.0005834 (PMC13475909; doi:10.1371/journal.pgph.0005834)
Supplement: Supplementary file 1 [file pgph.0005834.s001.pdf]

Focus Group Discussion Guide (WCLHIV)

Factors Influencing Access to Mental Healthcare Services among Women Caregivers living with HIV in Enugu, Nigeria

Audience: Women caregivers living with HIV (WCLHIV) including those caring for children living with HIV at selected HIV treatment centers in Enugu

Part 1 Introduction/Ice breaker (10-15 minutes)

NOTE TO MODERATOR: It is very important to build rapport with the participants and create a 'permissive' environment for discussion. During the discussion, you'll want to use an informal tone and have a relaxed and open body language. The discussions should be interactive and not dominated by one or 2 people (including the moderator!). The location you choose for the discussion can be either indoors or outdoors but make certain that it is **quiet, comfortable, private, and free of onlookers or other distractions**. Ensure all participants are seated in a circle at the same level where everyone can easily see everyone else – this seating arrangement helps foster an interactive discussion and diminishes hierarchy among the group.

***Moderator Script**: Good morning (afternoon/evening) everyone. I would like to thank you for coming today. My name is ……………………… and I have been asked by the Epidemiology Research Unit, Department of Community Medicine, UNTH to moderate this group today. Over the next 60 minutes, we would like to obtain your views and experiences with mental health problems following your becoming aware of your diagnosis with HIV, how you feel about seeking help in this health facility for mental health problems as well as what makes it easier or difficult to access mental health care. The information we're gathering will be analyzed and made into recommendations that we hope will ultimately improve the care provided to you and other mothers assessing care in this health facility. During the course of this interview, your opinions will be very important and we want everyone to feel free to say exactly what she thinks—there are no right or wrong answers. Everything you say here will be kept confidential and anonymous - no one will ever know what you personally said.*

As we begin the discussion, I will be writing down what you say (with no names of course! This is totally anonymous), but just in case we can't get everything down on paper, we're tape recording this session. We will use only one name for each person in our discussion today so that will make the conversation more anonymous. Because we don't want to miss anything, it is important that only one person talks at a time and that you share everything that you have to say with the group and not with only one or 2 people in the room. Please speak up if you disagree with what's being said - we want lots of different ideas and opinions and again, there is no right or wrong thing to say.

Does anyone have any questions before we begin our introductions?

Okay - Let's start by introducing ourselves and telling the group a little about your background, including your profession, how long you've been a mother and the number of children you have.

[NOTE TO MODERATOR: Start the introduction from one direction and go around the room. At the end of the participant introductions, make sure you introduce yourself and tell a little about your background as well]

Moderator Script: *I'm going to start tape-recording now and will begin by asking you about any mental health problems you may have had since you got to know about your HIV status*.

Question 1: How has your HIV status affected your mental well-being?

Question 2: How frequently do your healthcare providers (doctors, nurses and counsellors) make enquiries about your mental well-being?

Question 3: Based on your experiences, what factors do you think contribute to your mental health challenges or mental health challenges of other PLHIV?

Question 4: Do you know of any program or resources which are meant to address the mental health issues of PLWHIV in this facility?

If yes, Can you describe them?

Question 5: What are the obstacles or barriers to having access to mental healthcare services in HIV settings?

Question 6: What in your opinion are the possible solutions to the challenges of access to mental healthcare services in HIV settings??

Is there any other question you would like to ask me?

Thank you for taking time to participate in this interview.
