# Supplementary material for: Exploring barriers to and opportunities for improving mental healthcare access for women caregivers living with HIV in Nigeria: A qualitative study
Source: PLOS Glob Public Health. 2026 Aug 14;6(8):e0005834. doi: 10.1371/journal.pgph.0005834 (PMC13475909; doi:10.1371/journal.pgph.0005834)
Supplement: Supplementary file 2 [file pgph.0005834.s002.pdf]

Key Informant Interview Guide (Clinical Healthcare Staff)

Factors Influencing Access to Mental Healthcare Services among Women Caregivers living with HIV in Enugu, Nigeria

Audience: Health care workers providing HIV care at HIV treatment centers (Doctors, Nurses, and Counselors)

Introduction

Good morning/afternoon/evening Sir/Ma. My name is -------------------------. I am here on behalf of the Epidemiology Research Unit of UNTH, Enugu. The ERU is a specialized research unit of the Department of Community Medicine, University of Nigeria Teaching Hospital, Enugu. We are conducting this research to gain insight into the mental health needs and factors influencing access to mental health care by women caregivers living with HIV in Enugu, Nigeria. The purpose of this interview is to learn from your view and your experience relating to mental health challenges faced by HIV-positive mothers while caring for their children living with HIV, and use the information to provide policy makers with research evidence needed to design public health interventions for the prevention of mental health problems among women caregivers living with HIV in particular and the persons living with HIV in general. The interview will last for about 40 minutes. Your participation is voluntary and all information will remain confidential. I will also be using a tape recorder to ensure information is captured accurately. At any time during our conversation, please feel free to ask me any questions or inform me if you would rather not answer a specific question. At this time, do you want to ask me any questions about the interview? (ANSWER ANY QUESTIONS AND ADDRESS ANY CONCERNS)

May I begin the interview now?

[__]

Yes, permission is given

Health Facility…………………………………………..

Interviewee: Clinical Healthcare staff

Designation of clinical healthcare staff: Doctor ☐ Nurse ☐ Counselor ☐

Ice-breaker

Question 1: **Do you or any other colleague of yours have any training (professional training or on the job training) or experiences with diagnosing mental health challenges among persons living with HIV accessing care at your health facility?**

Question 1b: If yes, what is the nature of the training or experience and how long ago was the training?

Question 1c: If no training, would you like to be part of a mental health training in diagnosing and managing mental health challenges among this group of clients? And why would you like to be trained?

Question 2: How common do you think mental health challenges are among persons living with HIV?

Question 2b: Have you interacted with a patient with a mental health challenge?

Question 2c: If yes, what were their complaints?

Question 2d: How did you address the mental health challenge?

Question 3: In your opinion, what factors are contributing to mental health challenges among persons living with HIV accessing care at your health facility?

Question 4a: Are you aware of any programs, resources or facilities available for preventing, screening and managing mental health challenges among persons living with HIV accessing care at your health facility?

Question 4b: Are some of these programs, resources or facilities tailored specifically for Women caregivers living with HIV?

Question 4c: Have you ever used the programs or resources within the health facilities in preventing, screening and managing mental health disorders in WCLHIV or PLHIV?

Question 4ci: If yes, please what mental health disorders have you prevented/screened for/ managed?

Question 4b: How helpful have these programs, resources or facilities being in preventing, screening and managing mental health problems among Women caregivers living with HIV?

Question 5a: Are there factors that make it easy for Women caregivers living with HIV to access these programs, resources or facilities in your health facility?

Question 5b: If yes, what are these factors?

Question 5b: Are there obstacles or barriers (factors that make it difficult or challenging) limiting Women caregivers living with HIV to access these programs, resources or facilities in your health facility?

Question 5bi: If yes, what are these factors?

Question 5c: How can these obstacles or barriers be addressed?

Is there any other question you would like to ask me?

Thank you for taking time to participate in this interview.
