# Supplementary material for: Exploring barriers to and opportunities for improving mental healthcare access for women caregivers living with HIV in Nigeria: A qualitative study
Source: PLOS Glob Public Health. 2026 Aug 14;6(8):e0005834. doi: 10.1371/journal.pgph.0005834 (PMC13475909; doi:10.1371/journal.pgph.0005834)
Supplement: Supplementary file 3 [file pgph.0005834.s003.pdf]

Themes identified, categories, subthemes, codes, descriptions and quotations

Main Area	Theme	Category	Subtheme	Code	Description	Representative Quote(s)	Source Type
Approachability	Lack of awareness about mental illness/Limited mental health literacy	Barrier	Ability to perceive the need for mental healthcare services	Mental illness knowledge	Participant describes a lack of awareness of mental health and mental healthcare	*"Barriers will be maybe ignorance. If they don't know that uhm there is uhm things like that [and] maybe care or resources that will solve their problem, they will not access the care... So, ignorance, then the provision of that care and then acceptance. If they will equally accept it okay somebody has mental health issues and you refer the person to psychological unit [but]...if the person doesn't accept that he is even having a problem, the*	KII - P4 (Nurse)

						person will not go"	
Availability and accommodation	Unavailability of integrated HIV-mental health care	Barrier	Ability to seek	Mental healthcare availability	Participant mentions a lack of mental healthcare services, a lack of knowledge of mental healthcare programmes or the need to provide mental healthcare services	*"Right now, right now, right now, not just no, right now, absolutely no, I'm not aware"*<br><br>*"I have not, I am not sure of that one [availability of mental healthcare service or programme]"*<br><br>*There's no special [mental health] programme. The only thing I know is that we have like their support group meetings that help them, but not specifically to mental health*<br><br>*"What I want to say is that, you people should try*	KII – P1 (Clinician)<br><br>KII - P12 (Counsellor)<br><br>KII – P7 (Clinician)<br><br>FGD - P14 (Woman caregiver)

						and get a [mental health] department here…Please, it will be better for all of us if mental health became a department here"	
						"No, I have not heard about that one [mental healthcare service availability]"	FGD - P20 (Woman caregiver)
						"I don't know of any [mental healthcare] programme"	FGD - P27 (Woman caregiver)
						"I don't know but all I know is that if we identify such patients, we usually refer the person to go and see the doctor, our clinic, the clinician here. So, doctor will now assess and refer the	KII - P10 (Nurse)

						person to see a psychiatrist. You know [this health facility] has psychiatric unit"	
						"In fact, I referred one to psychological clinic…and she never came back… that's the problem, you refer there, you do not get [feed]back, so you don't know what happens"	KII - P1 (Clinician)
	Lack of training for HCWs providing routine HIV care on mental health care	Barrier		Training gaps	Participant describes the need for training of health care workers providing routine HIV care to be able to provide	*"One of the barriers I see is [lack of] training. We need to be trained. So that you may… pick the signs at a very early stage. Because if you are not trained, you may not pick*	KII - P7 (Clinician)

					mental health care	*it... so training is important"*	
	Existing support groups	Opportunity	Ability to seek	Resources and programmes	Participant describes improved mental health care access because of the availability of resources or programmes (such as support groups, savings and loans associations)	*"...support group, when you're coming out, the thing is they say that [an] idle mind is the devil's workshop. I believe that if you're coming out, you'll be learning so many things...when you come there [support group meetings], you'll see people with sickness but they are happy, because they are meeting [...] people that can teach them, people that can talk to them, make them to ease their pains. Yes, so I believe that, that social [...] support group helps a lot"*	KII - P12 (Counselor)

						"…there are groups that help. There is the Orphans and Vulnerable Children [support group], apart from the support group, especially mothers maybe that have children that are positive so they help them"<br><br>*"There's a meeting that holds every Saturday in a month. When you come to the meeting, they address things like this. Instead of overthinking, the things they talk about in the meeting, helps to encourage us […] We also get to meet people like us which is comforting"*	KII - P7 (Clinician)<br><br>FGD - P38 (Woman caregiver)

	Economic empowerment schemes	Opportunity				*"…I know that Village Loan and Savings Association, has been going on here…so you'll be saving, no matter how little it's something, at the end of the cycle when they will share it, you carry your own savings. Ehen, then it will help you, even your school eh, your children's school fees, it will help you, their clothing, food, so many of them, so many of them that you can use your savings to achieve"*	KII - P11 (Counsellor)
Acceptability	Stigma	Barrier	Ability to seek	Concealment and anticipated rejection	Participant mentions efforts to hide identity or fear of being treated poorly	*"Then most of them too, we found out that most of them from the statistics, most of them come*	KII - P8 (Clinician)

						from very far areas…They want somewhere far from where they stay because of the issue of stigmatization and so it's also […] a barrier"	
						"…There are some people that are shy. They wouldn't want to run into people that know them"	FGD - P35 (Woman caregiver)
Appropriateness	Long clinic waiting time	Barrier	Ability to engage	Time challenges	Participant expresses frustrations regarding length of time spent when accessing care	*"…the waiting time in the clinic is a barrier, so we try to shorten the waiting time but if it's not shortened it will affect them, they will just pick their medication and go home, neglecting this mental health […] issue and it keeps aggravating—you know mental health once you*	KII - P4 (Nurse)

						don't treat it immediately, it keeps aggravating [and] it keeps getting worse and worse, and it turns into another thing. Somebody that has anxiety today may come down with depression tomorrow and start talking about suicide the next time"	
						"One thing I want to talk about is time. Some of us are public servants and civil servants. Like me, I told my [supervisor that] I want to rush somewhere and come back and I have been here since eight o'clock. If you can help us, [...] adjust the time to	FGD - P18 (Woman caregiver)

						favor us in our works"<br><br>*"If you come early, you are meant to go early whether you work in the office or you sell goods. But we will come here, and the nurses and doctors waste our time."*	 <br>FGD – P13 (Woman caregiver)
Affordability	Transportation costs	Barrier	Ability to reach/Ability to pay	Transport challenges	Mentions related to the high cost of transportation or geographic distance.	*"Hmm, some will tell you that we are coming from a very far place…that they won't be making it because of transportation"*<br><br>*"For me the challenge that I observed is challenge of transportation fare"*<br><br>*"The problem, what they usually complain, most*	KII - P11 (Counsellor)<br><br>FGD - P10 (Nurse)<br><br>KII - P8 (Clinician)

						of them when they miss appointment and you call them, it is usually transport fare that is their problem. Usually, that's why they miss their appointments"	
						"The second one could be that the person doesn't have transport money to attend or come for the meeting. Like me, I do well in the support group. I attend with my child but I haven't been able to attend this year because I relocated […]to a farther place"	FGD - P35 (Woman caregiver)
	Productivity losses	Barrier		Livelihood and income loss	Participant describes any concern	*"Most of them having to leave […] what they are*	FGD - P8 (Clinician)

					about economic loss or economic activities forgone to access care	*doing; they cherish it so much. Some of them are farmers, and it could be time of harvesting so it's going to be a barrier. Whatever time of the year, whether it's rainy season, they are in their farms, dry seasons they are harvesting"*	
						"There are some people that get prepared to attend but won't have transport money while some may not have chance to attend. There was a woman I reminded about the meeting, but she said she wouldn't have time because she was going to the market to buy	FGD - P37 (Woman caregiver)

						foodstuff for her family"	
