# Supplementary material for: Exploring barriers to and opportunities for improving mental healthcare access for women caregivers living with HIV in Nigeria: A qualitative study
Source: PLOS Glob Public Health. 2026 Aug 14;6(8):e0005834. doi: 10.1371/journal.pgph.0005834 (PMC13475909; doi:10.1371/journal.pgph.0005834)
Supplement: Supplementary file 4 [file pgph.0005834.s004.pdf]

Transcript of key informant interview with healthcare worker 1, Facility X

I: Good afternoon, ma'am
R: Good afternoon

I: My name is [].
R: Okay

I: And I am here on behalf of the Epidemiology research unit of [name of facility], Enugu. Uhm we are here to get your perspectives on access to mental health care services in this your health facility for women caregivers living with HIV
R: Okay

I: Uhm your participation is voluntary and all information will remain confidential. I will be using a tape recorder just ensure information is captured accurately. At any time during our conversation please feel free to ask me any questions or inform me if you will rather not answer a specific question
R: Okay

I: Uhm at this time will you want to ask me any question about the interview?
R: No. I'm good

I: Alright thank you very much. So, may we begin the interview?
R: Okay

I: Alright thank you very much. So, ma the first question we have for you is uhm, do you or any other colleague of yours have any training, either professional training or on the job training or experience with diagnosing mental health challenges among person living with HIV that are accessing care at your health facility?
R: Okay so formal training

I: Or on the job training?
R: On the job training

I: Anyone at all
R: Well, no, no on the job training. But I think in the course of residency training, we are expected to learn about HIV, care for HIV patients and mental health. So indirectly yes.

I: Okay so thank you very much ma. So uhm but will you be willing or like to be part of a mental health training uhm that centers around diagnosing and managing mental health challenges among these groups of clients?
R: Yes, very much

I: Okay and may I know why you will like to be uhm trained on this particular aspect?
R: Okay because mental health is a topical issue at the moment. A lot of people ignore their mental health. So even when people have signs of some mental health issues, they suppress it and because of the way our community and our environment is structured, people feel they should hide whatever mental health challenges they have. So, women already they are like a disadvantaged group and then the stigma attached to HIV uhm patients as well compounds the problem so it actually likely that it is like a two-- double arrow attack. So, you are a woman and then you are HIV positive as well, so your mental health is really problematic, so yes.

I: Okay thank you very much ma. Uhm let me ask you this question, how common do you think mental health challenges are, among persons living with HIV?
R: I actually think they are much more common than you would expect. Uhm because like I said stigma is a major thing, you know, they have stigma, they have the pill burden, they have you know-- they have a lot of things they are trying to organize in their minds. You know, especially when you talk about a mother who is uhm coming to terms with the fact that she has the disease and then she may also have passed it on to their children. So, there's also that thing of guilt and then the stress and you know so-- definitely I think it is under reported

I: Okay
R: Or it goes undiagnosed

I: Okay
R: In most cases

I: Thank you very much. In the course of your work, your daily work at this health facility, have you interacted with a client with a mental health challenge?
R: Hmmm. So yes-- yes, I would say yes

I: Okay and uhm just for clarity. Mental issues are like--
R: A broad spectrum

I: A broad spectrum so it could range from anxiety—depression, even stress, stigma as you mentioned earlier, all the way to the you know over psychosis issues. Any range of these is okay, we just want to understand if you have had any interactions at all in the course of your
R: (cuts in) Yes, I have

I: ... duties with clients with any mental health challenges
R: Yes, I have

I: Okay, could you tell us what were the complaints or what they were exhibiting that made you uhm believe that they had some clinical issues relating to their mental health?
R: Okay so for instance we had a mother who uhm—who lost uhm—she was a widow but she had children. So, she had uhm-- she was still—after she lost her husband, she still lived in the family home. And at some point, she started saying the cowives were trying poison her, were trying to kill her, now she has children but her children are not positive. She had three kids, so at some point her brothers from her own paternal family came and took her back because they noticed she started exhibiting some signs of psychosis, like she just wasn't coherent most of the time and she was having voices speaking to her, she was acting erratic. So, she was moved back to her father's house with her children and then the brothers said in the middle of... and they realized she stopped taking her pills and she would lie about it. So, the brothers will bring her to the clinic give her, her drugs. So, she was brought back so sick that she had to be placed on admission. And the brothers said she woke up in the middle of the night one day and it was pouring rain and she ran away and left her children in the house and it took them sometime before she returned. So that was just a clear-cut case of psychosis, and then she was telling me oh people—voices in her head, they keep telling to go, eh they keep telling her if she stays she will kill her children so she had to—she had to go.

I: Okay
R: So that was the most extreme case. But then I've had cases of mothers who bring their babies and then they are like in denial, you know. So, they just keep telling you it's not their portion, it's not their portion you know. So, but you know they don't—you are not sure until that one-year mark and then you know that bell they ring so until that one-year mark you are not a hundred percent sure that you've not transferred it but then you just see mothers everyday just telling you no—no—no it's not my babies' portion, my baby is not going to have it you know. Most of them are even reluctant to come for appointment or do test or do you know give drugs to their kids

I: Okay thank you very much ma. So uhm my next question would be a rider to your response. I will just like to know how you addressed uhm you know one or both of these mental health challenge scenarios you described?
R: Okay so for the lady that had the clearly obvious psychosis. Well, I counselled her and then I invited the social workers to come seat with me and we talked with her, we talked with her siblings, you know made them understand that she is not doing this to be wicked cause you could see her brother was already frustrated, you know they felt she was such a wicked person and they kept complaining. They showed us a big bag of drugs she collected the last time, she hasn't taken anyone, no matter how they begged her, cajoled her, forced her, she is not taking the drugs. So, I counselled her and I reiterated the importance to her of being—you know being consistent and regular with her drug intake. We invited the social worker and then still wrote a referral. So, while she was on admission, the psychological medicine team met with her and began managing her. Unfortunately (chuckles) I have not followed up.

I: Okay no problem ma. So, uhm my next question would be, in your own professional opinion, uhm I know you've mentioned a couple of them earlier on, but what factors are contributing to mental health challenges among persons living with HIV that are accessing care at your health facility?
R: Uhm I think coming to terms with HIV as a disease, the pill burden, having to take pills every day. Uhm the economic situation is harsh. Most of them struggle to get money to pay their transport fares to come, most of them struggle to get money to do their test and they must do this test regularly. So, you know they have a lot of these stressors. Uhm, we have some people, a lot of ladies that are teachers in private schools, the schools don't give them permission, they won't give them days off to come to the hospital to – you know so a lot of these things weigh down on them and they can't quit those jobs cos they need the money. A lot of them are hiding it from their family members, they don't have the support, the fear of stigma, stigmatization. So, I think there are quite a few other but this is—

I: Alright thank you very much ma. So uhm are you aware of any programs or resources or facilities available for either preventing, screening or managing mental health challenges among persons living with HIV that are accessing care at your facility?
R: (silence) Uhmm no but for adolescents. Okay, so there—initially the []uhm adult clinic had a support group, I have forgotten what it's called. The support group used meet at the old site but then lack of incenti—incentizi

I: Incentivization
R: Incentivization-- made it fall apart. But then the adolescent group, they still have an adolescent support group, the OTZ, operation triple zero. So, within that group they have regular programs you know for the adolescent. So, they help them handle stigmatization, they help encourage them through pill burden, so, operation triple zero has its pillar and these are the pillars targeting things that could cause nonadherence and non-compliance. So it tackles mental health issues as well but then the adult clinic, no, not anymore

I: So, what you are saying essentially is that they are really no programs or resources available for the adults?
R: Yes

I: And especially for women caregivers living with HIV that you are aware of?
R: Yes

I: However, a support group is in existence but focuses more on adolescents
R: On adolescents but adolescent mothers have theirs too, OTZ plus.

I: Okay
R: So, women who have children who are within that adolescent age range yes

I: But do you know if that aspect of the support group is you know is fully operational for adolescent mothers in your health facility?
R: Yes, it is fully operational and I think its not for adolescents, I think it's for young mothers

I: Young mothers
R: So, it gets up to 24 years of age or thereabout

I: So, do you know any specifics about this particular program that is tailored specifically for these young mothers who are HIV positive?
R: Okay so I think uhm mainly what they – okay so it's called— it is separate from the regular adolescent's support group that is the OTZ. It is called the OTZ plus so it's meant for like young mothers. So, I think pretty much what they do is just to provide extra support for them. So, there's a lot of counselling, there's a lot of activities like uhm extracurricular activities organized for them. I think basically it's just mainly founded on counselling. They really do little or no— but then they have like end of year activities, they have philanthropist sponsoring, parties and stuffs like that for them so, but uhm I don't know if they actually engage them in any—I don't know honestly

I: Okay alright no problem ma. Thank you so very much. I will just like to know from your own perspective, what other programs or resources do you think can be made available for prevention, screening and managing uhm mental health challenges among persons living with HIV coming to receive care at your health facility? In addition to—
R: what is already there

I: -- what is already available
R: Okay so I think definitely we need an adult support group so maybe a larger adult support group but then like with subgroups where you have like the women group, you have the men's group, you have—so that the support is equit— equitable

I: (inaudible speech)
R: Support you know pretty much

I: Okay alright thank you very much. So uhm have you ever had course to use the program or you know refer a client to these program that you've just described? Particularly a young mother that is HIV positive?
R: Yes, I have

I: Okay uhm. So, do you have an idea or can you take us through any particular mental health disorder that you think uhm that resource or those resources available were able to be used to either prevent, or manage or screen for?
R: Hmm. Okay so I think uhm it was essentially what they try to do for the most part was uhm to support this mother through the uhm preg—PMTCT essentially. So, they were—so she had the contact number of a nurse who she was allowed to call anytime of the day or night. Uhm she had people checking up on her you know calling her regularly, ensuring she was okay, uhm she was introduced to other mothers like her, who had gone through the program, who adhered to all the advice that were given and how they turned out healthier and had healthy babies and uhm I don't know essentially

I: Alright thank you very much ma'am. You've given us very great insight. So uhm just as a rider, uhm how helpful uhm do you think uh this specific program you mentioned for young mothers living with HIV, uhm how helpful has it been either preventing, screening or managing uhm mental health problems particularly among the young mothers who are living with HIV?
R: Okay so I think it's been quite—I think I would say it's quite helpful for the preventing, for the screening and the management because they give a lot of health talks, so they give a lot of health talks, encouraging them. So, that's part of the prevention. As per the screening, they have one on one counselling with the nurses, and the staff, the social workers that are attached to the program, so you explain to them what your problems are and then they can refer. Well, they can't refer directly but then they would speak to a doctor who would refer to psychological medicine. But I think most of the time they found that they can handle this thing within themselves just by constantly following up and you know calling them constantly and you know they organize a lot of uhm fun activities you know. So, they have a lot of people sponsor them. So, they have a lot of fun activities where the young people can just come, you know hang out together, let their heads down but one constant is they always have health talks. So, they still keep encouraging them, telling them you know, you have to take your tablet, but do you know that people that have cancer wish they had HIV because there's a cure you know, they do a lot of those things

I: Alright. Okay thank you very much. My next question is uhm are there factors that make it easy for you know these young mothers or other caregivers living with HIV to access these programs in your health facility? Or this specific program that you mentioned in your health facility?
R: Okay are there factors?

I: Yes, are there—are there factors that make it easy?
R: (cuts in) easier

I: That enable them to you know key into this program?
R: Okay so I think at the point of enrolment, most of them are just migrated into the program automatically. They don't have to go through any bottlenecks, so at your point of enrolment once you are within the age group uhm you are automatically enrolled in the program and then the—everything just follows subsequently

I: Okay alright thank you very much. But then are there barriers you know that make it challenging or difficult or even limit women care givers living with HIV from accessing programs such as the one you just described?
R: Hmmm (silence) Yea well. Okay so making their way to the facility may be the most major barrier but then I think deciding to go to the facility and actually coming to the facility will be the barriers I would identify. But I think with regards to enrolling in the program once they are in the facility, I think it's pretty straight forward once you're within the age group. They've been known to take mothers up to 29 years, even though there's always a conflict with uhm you know the adults but they try to take young mothers up to 29 years of age. So, I can't think of any barriers within—

I: --the facility
R: Yes

I: Except you know distance or the cost of transportation to the facility you mean?
R: Yes

I: Alright thank very much. Uhm so this barrier that you mentioned now that the distance factor or the cost of transportation to the facility that you mentioned, how do you think is the best way to address this barrier to minimize its effect on the clients?
R: Okay, so the economic situation has made it you know hard for everybody, so it's hard across board but if a person understands the importance of that visit, then they will be willing to make it work. If they understand how important it is for them to get to the facility to attend the uhm consultations, and the meeting and get their prescriptions. If someone understands that, then the person is willing to sacrifice to pay you know for the transportation and then disregard the distance just to get the—but then that being said OTZ is not only in [this facility]. So, they have like in various other facilities, so they may have uhm facilities, everybody has a facility that's proximal to their community somewhat. So that may –

I: ...minimize the impact of socioeconomic and transport barrier. Alright so uhm thank you very much ma'am uhm is there any other question you would like to ask us?
R: Hmmm no. Thank you.

I: Alright, alright.
R: What would be the recommendation? What would be your recommendation? Like what is it?

(both laughs)
R: okay so I think if I had to recommend, I will say okay that uhm the most important thing I would, in my opinion is to have a support group for the adults. And then these support groups would not just be for the caregivers, would have medical personnel attached to the support group. So, in as much they are discussing their issues among themselves, providing support for themselves, you have medical personnel within them who are able to identify you know these telltale signs of mental illness. If someone recalcitrant, like you are coming every day, other people will cheer up after sometime but you're persistently depressed or (sighs) unmotivated and all that. And then obviously the medical personnel need to be trained on these-- finding early signs of mental illness. We don't have to wait for full blown psychosis, so you are able to ask all those demotivated questions like do you still enjoy doing things you use to do before? do you feel like staying away from people more? So, you know we ask all those questions but people don't know what questions to ask if they are not trained. So, you are trained to detect early signs of mental illness

I: Yea, okay. Thank you so very much ma
R: Thank you

I: ...for your time and for participating in this interview. We really appreciate
R: Thank you very much

Transcript of key informant interview with healthcare worker 2, Facility X

I: So, we have few questions to ask you hmm, and whatever we are going to ask you. (Anything we will ask you, let me say it in Igbo---) (background noise)
R: Speak English, English is better, because the person that will transcribe might not be an Igbo person.

I: So, whatever-whatever we will ask you, whatever we are going to ask is that, we will record it
R: Sure, sure

I: So, and it's just about your work in HIV clinic, or what you've been doing
R: Fine

I: Biko, so we can go ahead
R: Please go ahead

I: Okay. So, I want to ask you eh, do you know, do you have any training or experience with diagnosing mental health challenges among people living with HIV that are accessing care eh in HIV clinic
R: Before I started or the length of time, I had in HIV clinic?

I: Yes, yes
R: Yes, I have seen a few, but very few

I: No, do you have any training, (you yourself), you particularly?
R: Any training about the people having mental challenges?

I: Yes
R: No

I: Okay, do you know of any colleagues that have been trained in mental health eh services?
R: No, no

I: Okay, but will you like to be part, incase if you want-
I2: I wanted to ask him, but in the course of your work, you may not have had any formal training but in the course of your working in the HIV clinic in [facility name], or in the health facility where you work, have you had any experiences whatsoever with diagnosing mental health challenges?

R: Yes

I2: Among persons living with HIV?
R: Yes, I have had some and I felt, it's part of HIV in fact uhm eh, if-if it classifies HIV, World Organization uhm eh 4, mental challenges is there, I have seen some, okay but it's-it's rare, if I have seen 100 patients it's not up to 1 patient. It's not up to 1% sorry, not 1 patient, 1%.

I2: Okay
R: But I feel we should recognize it as part of HIV pha-uhm, the classification, should be class 4, the mental challenges I have seen. Thank you.

I2: Okay alright
I: Would you like to be part of the mental health training?
R: Yes

I: Why will you like to be part of the training?
R: Because, as a person working in HIV clinic, I want my patients to get better, and if I'm trained and I can train or I offer the services to some others, that would be a very good thing to do

I: Can I ask you a question, how common do you think mental health challenges are among persons living with HIV?
R: Uhm eh, I will say it's just the social statistics, because I'm (Unintelligible words) but right now, those challenges have gone a lot far to deal with the issues, because people have learnt that living with HIV is not a stigma, it was before now that people felt so much, so those mental challenges now depends on the person (inaudible speech) and you can't put it the same for all of them. Most people that live with HIV now, once they come in, the voluntary testing, you'll be told you have it, and you'll be educated, you'll be given enough of knowledge for good, you're not the only one, all of us here- in fact I have told some patients that I have, so that to just convince the patient that there's not stigma about it.

I: But before you give the counselling, I just want to know, as in, once they come in, do you- or maybe during the course of treatment, are they exposed to mental health challenges, as in like how common do you see these patients before you-
R: Ehm I'm looking at this your question, mental challenges and psychological issues, is it the same?

I & I2: Yes, yes

R: I don’t agree with you

I2: So, the mental challenges we are looking at, we are not restricting it to-
R: The psychological challenges we are talking about, mental challenges, the person gets-

I2: We are not restricting it to psychpious psychosis or that level of some-, we are looking at even-
R: (Talks to an unknown party: I’m in an interview, please one second).

I2: Yes, go ahead please, go ahead
R: See every other patient remains a patient and eh we will say that patient remains a patient. (Unintelligible words) (Takes a private phone call) So far so good, many people are more educated that HIV or AIDS does not mean death, people have learned so much that they are now easy to get access and they know. At least in our centre in [], when you come in you have time limit, you come in, we can deal with any issue you have and we make sure you don’t sit too long, one, two, the second point is that we have made things quite easy for anybody that has HIV, but I can have it, you can have it, so we have dealt with that challenges not fully but up to an extent, but the mental challenges, the psychological challenges is a lot less now than before. I can’t give you a percentage, roughly now, percentage those are, those that have not learnt, that we wouldn’t have talked to, every morning we teach we talk to our patients and they will talk to others, we have people have combined so that HIV positive will marry HIV positive, we have told HIV positive if you bring your viral load down, you can marry HIV negative and then we are achieving. Right now, preponderance and even the incidence is reducing because of so much efforts have been done. We thank everybody for doing so with us.

I2: Thank you, thank you Sir. So-so Sir ehm, ehm, we want to now be a bit more specific to know
R: Sure, go ahead

I2: In the course of your work ehm, have you had-have you had patients or interacted with any patient that you perceived had, you know, either a mental or psychological challenge while the person has come to receive treatment in your clinic
R: Yes, two, two.

I2: Okay

R: In fact, I referred one to psychological clinic because I felt it was HIV, now we have, in stage 4, (Unintelligible words) stage four, there are certain challenges you- (inaudible speech) stage 4, I have had, two, but I referred one to psychological clinic and she, was a she, she never came back

I2: Okay, okay. Thank you very much. So, what were the complaints that that patient had or the manifestations that you saw that made you refer to--
R: There are difference sef, the difference is symptoms and diagn- ehm, symptoms and signs, okay. The symptoms, this patient taking her drugs was eh she, infact both I had was shes.

I2: Okay
R: Unfortunately (laughs). No, I think, the actual issue, she was just misbehaving. one, she was having hallucinations and illusions, no, she thought it was the drugs cos she was on, she had been on first line for a couple of years, ehm I think up 8 years, when we go into the hospital we can pull those folders. About 3 years after being on second line, she started having psychological issues, please I don't know why I don't want to use mental

I2: It's okay
R: Started having psychological issues, she was complaining that her husband was telling her to take drugs which she has been taking. I think it's a complaint which I look at as a symptom, signs I didn't see any sign, I didn't see anything physical with her, and she was doing well, weight she had gained weight, skin texture got so well and so good. She was doing so good, but her problem was, that her husband was forcing her to take drugs, and they were- both of them we will call it- both of them were positive-positive, so the husband was taking drugs but she stopped taking her drugs and when her husband says take your drugs, she will (Unintelligible words), and they came, she agreed they came to my clinic and I discussed with them, her problem that the husband is challenging her, I now asked a question, what's the problem. From day-day, from my point I have seen some patients not in HIV clinic, have you seen a patient that has Alzheimer's disease, dementia. She was talking like someone that has Alzheimer's because she no longer remembers what has happened before, she said no, I have never been taking antiretroviral, I have never been taking ART, my husband is saying I have been taking ART. I said this is what you've been taking she said--.But its only two patients that had issues then, it's only one client, but with talking to, she-she came back, I involved the (*Unintelligible words)* the counselling unit, they took her, we've been able to convince her to continue her drugs. Today she's viral load non-detectable.

I2: Good
R: So, fine

I2: Good, good, good, thank you, thank you Sir. So, so you've already told us how you addressed that challenge, so our next question is eh, in your own opinion as a professional,

uhm what are those factors that you think are contributing to psychological challenges among persons living with HIV coming to access care at your-at your facility
R: I think the problem is ehm, eh, social and psychological because they don't want you to come into that clinic, with this eh, this is my driver

I2: Okay
R: He's okay, but the first time we came to the clinic, he was like, they will think he has HIV, and I said you mustn't have HIV to come to this clinic

I2: Hmmmm
R: I think it's just a social issue, I'll answer that question please

I2: Alright, okay, thank you
I: So, what are the social issues?
R: Social issues like look at me, I'm a doctor I'm going to the HIV clinic and you don't know me, and you see walking inside there, you will feel like everyone that comes there is HIV positive. Now, we have been educating, it is because it's a kind of stigma, I don't want to have HIV, but if I have HIV, I will not die. And I will tell you something, I have told my patients it's better you have HIV as you have now, than you have hypertension or diabetes. Fine, I'm done.

I2: Alright chief, thank you very much. So now, we-we want to look at ehm, whether there are available resources for helping people who may have some of these psychological, ehm, challenges that are also HIV positive, so we wanted to know, are you aware of any programs or resources available for preventing-
R: Right now, right now, right now, not just no, right now, absolutely no, I'm not aware.

I2: Okay, okay, okay, so-so we wanted to know as a rider to your answer, since you say you are not aware of any such programs or resources available at your health facility, eh, but what kind of resources do you think would be appropriate to make available to help eh tackle psychological challenges among HIV positive persons?
R: Psychological challenges?

I2: Yes
R: I don't know, its eh, those like myself and some others in that unit, we will think about it and ask the management of the hospital, cos as if you said, if the local place has a way, then go to the higher place so we can ask of some resources, could be easier. I want to put something here now, in the unit in HIV clinic in [name of facility], sometimes you go and ask for gloves, they say the gloves have been provided for us, we should get from or HIV clinic or ART clinic, the hospital does not provide for you. That's the greatest struggle I have as a person, right now I think I'm the

lead clinician in HIV clinic now, I say, I ask for gloves they say I should go and ask for-and get it from it nurses, no, I think it's wrong, I'm done.

I2: Okay sir, alright. So-so we almost at the end of our interview, and erm, we wanted to know, earlier you mentioned that you had to refer one of your clients that you perceived had some psychological challenge to psychological medicine.

R: Yes

I2: So, we wanted to know, if uhm-did you get any feedback to know whether that intervention helped

R: No, that's not our problem. Repeat that question so I will answer it well. Let me now help you.

I2: So, we wanted to know whether referral because-

R: No, that's the problem, you refer there, you do not get back, so you don't know what happens. It's actually there (*Unintelligible words*). What do you do? I wouldn't go tracing the patient, I was expecting, I sent a patient to you, it's a referral and it should go in two ways, we work together now, so, unfortunately it didn't happen that way.

I2: Okay so the next question we want to ask you sir is ehm are there factors that make it easy for, particularly women who are HIV positive and are also caring for their children who are also HIV positive to have access to resources to help them with their psychological challenges or mental health challenges as the case may be?

R: Okay let me tell you the truth, right now, as far as I'm concerned No

I2: Okay, okay. So-so there are no factors that you think might make it easy?

R: I think there are that can make it easy but I disagree with you

I2: Okay

R: Each time they say women, why not the men

I2: Yeah, so-

R: Why only women, the men that are positive, why-why don't you deal with men and deal with everybody. You're telling me women, I don't accept that, please.

I2: Okay, okay, no problem. So, you feel that whatever resources that should be made available

R: Should be to all

I2: Should be for everyone not just women and caregivers, alright

R: Yes

I2: Okay Sir. So now we've talked about whether there are factors that make it easy, how about you tell us if you are aware or know of factors that make it difficult, like in this case now the patients that you saw, to access-to access ehm you know resources to help with their psychological challenges
R: I will tell you, I have seen, in fact women are being helped, as a person because it's a woman, in my life I will never fight a woman, I will fight you as a man. So, because, firstly I think the woman is uhm not as strong as a man, and if a woman have challenges she's going to fall immediately, and if a woman falls in the family there is a lot of problem. So now, but I will not see a woman because she's a patient, the other man is patient I will see the woman first, no. But I will not see a woman that I say is heavy, what I mean heavy, pregnant. Or is breastfeeding, I must make sure you come fast. In that same way, I won't see an elderly woman or an elderly man, you can jump the queue, we will see you and you go, that's respect.

I2: Alright sir, so-so can you-can you tell us a little bit more about some of these obstacles that make it difficult for persons living with HIV accessing the help they need for their psychological challenges
R: Ehm, I think physical challenge like I can discuss about, psychological challenges I'm not sure, okay you asked a specific question let me answer psychological challenges. Physical challenges I feel, I'm coming here because someone is looking at me, and I'm coming, he may run away it's the same thing that the person that has HIV. He's so, not being afraid but shy, not shameful but shyness or some- the persons don't want to walk into that clinic. We have had those challenges and we have even tried, if you don't want to come, you send anybody coming, we can come and see you under the tree, anywhere, anywhere we will see, because we insist and maintain that the important thing is immediately, we diagnose that this person is HIV positive and then test and treat and we start treatment, that has reduced incidence, in fact even reduce preponderance, I'm done

I2: Okay, alright chief, so uhm, we are almost at the end of our interview, we want know if you have any question for us as a research team that are asking you these questions
R: Okay, now I want to ask you, is it research from [facility name], or is it [] and others?

I2: Laughs
R: Okay I have asked my question

I2: No

I2: …Chief thank you so much for-for your time. We really appreciate your time…

Transcript of key informant interview with healthcare worker 3, Facility X

I: Okay, so, the first question, which one are you conversant with, should I use English or Igbo?
R: English.

I: English, fine, thank you. The first question is, do you have any training or experience with diagnosing mental health challenges among persons living with HIV, accessing care at your health facility?
R: No.

I: Eh?
R: No.

I: Okay, you don't have any training. What of your colleagues, have anybody been trained on mental health services?
R: No, okay, uhm, I wouldn't know whether there was a training for her, but I know that Chiefo, eh, she did work, Chiefo did work with eh Red-Aid on that, some of the cough monitors, but that is in aspect of tuberculosis.

I: Okay Okay, so but after the training, did they step... do any stepdown training for you people here?
R: No, no, no, no.

I: Okay, thank you. So, would you like to be trained on mental health, for you to give care to our people, our patients in this facility?
R: It is very important, yes, I'll be willing to be trained.

I: So where do you want to be trained?
R: The truth is that... you see eh, uh, most of our patients are emotionally troubled, and I know that emotion has a-a-a high role to play in psychological balance, so, and if they can be managed well, by our aid because, it's just like we come in contact with them all the time. It will improve our services to them and it will help them to be adherent to their drugs.
I: Alright, thank you. So, then the next question is, uhm, the... sorry, let me ask, the-the nurse that was trained, do you know how long she was trained on mental health?
R: I wouldn't know whether she was even trained

I: Okay

R: I wouldn't know whether she was trained, but I know that they were doing something on Red Aid

I: Okay
R: Ehen, and it's still ongoing, based on depression, substance use and all that.

I: Okay, and when was that?
R: It started eh (inaudible speech) Chief!

I: Don't worry, don't worry just let's continue, let's continue. So, you don't know when it started?
R: No.

I: It's okay, let's continue. So, how common do you think mental health challenges are, among persons living with HIV? How common do you think mental health challenges are, among persons living with HIV?
R: Mhmm, it's very common, it's very common but I see it more with those ones, erm, more with the adolescent age, those that are having family issues, because we've had cases of those ones that because social welfare couldn't come in, they went into depression and they-they were, they landed in psychia. Then another set is eh, the-the pregnant ones or those ones that have small children, you know that does not have eh, eh, child spacing, especially now that the economy is bad and those ones that have marital issues, cos some of them, their husbands are challenges to them, so of course.

I: So, in this clinic, I want to ask you eh, in this our clinic now, how many patients, like, from your own opinion, how many patients have you seen with mental health issues?
R: If I should take a count with the ones I've come in contact with, I know I would have said I have been in contact with up to like eh 40.

I: 40?
R: Yes, 40, blown, this one is full blown, it's obvious, you don't even need to screen the person or when you now talking of the small small ones, like the ones you see signs, you know, and all that and all that, you start saying up to 100 now.

I: So, these ones you're saying, these ones you say you are seeing, so, what were their common presentations? As in, what was the common symptoms they presented with?
R: First of all, physical appearance, no grooming, physical appearance, no grooming, eh, they are lost out of time, ehh, place and position. They don't even know anything about themselves health wise, you know, physical presentation is... appearance is zero.

I: Apart from that, what other symptom do they present with?

R: Then, erm, they soliloquize, they talk to themselves, they are absent minded, they, some of them, they lose sight. They will, they can stand here and you will just be calling their name, they are looking at you, you're calling somebody's name, the person is lost, by the time he will remember himself and, will now like you know, ehen. Then, erm, there's this three that always be, you know, flipping through books, just flipping through books, and you know, when you now call them, there's even the religious fanatics among them, they will just carry bible and come here and be preaching, just be preaching and preaching and repeating the preaching and repeating the preaching.

I: Okay, so, I want to ask eh, these people that you saw with these symptoms, what did you do as a health care worker in addressing this their problems?

R: Oh, the thing is that, I will attend to them fast so that they will go (laughs), because-

I: Apart from that eh, in terms of the mental health issues that they had, did you do anything to address it?

R: No, no, because there's not, we know little, or nothing to it, except if the doctor now refers them to psychia because there are two that already, that we referred to psychia, so those two, like one is eh Blessing and the other one Nnaji is pediatrics, we referred them back to psychia, ehen, yes.

I: Okay, thank you, thank you. So, most times you refer them to the place?

R: We refer psychia or you just discharge them sharp sharp so that they don't come and cause trouble for us in this place (inaudible sound).

I: Okay, so I want to ask but those ones that you didn't refer to psychia, did you do anything or just gave them drugs and they go-went home or what?

R: You give, you just attend to them, give them their drugs so they will be going

I: Then those that you referred to psychia, did they come back here? Were you able to identify them again after erm, being seen by the psychiatrist?

R: The-the-the-the performance is-is poor, is usually poor.

I: What do you mean by the performance is poor?

R: When you rate them, like they are in psychia now, they should improve now, if they are in psychia, but, (makes a sound).

I: But do they normally come back to you?

R: Yes, they-they do, they do, we still have some of them sef still here, they do.

I: Okay.

R: But those ones that have eh, emotional challenges, that they are still tending to, you know these ones, the ones that I talked about previously, are the full blown ones, but these ones that are still tending to, by the time you call them, and start, you know, discussion, counselling and all that, somehow there is a threshold you will get to in that counseling they will open up, and I feel that after such counseling, they do better. The next time you see them, there is improvement, but the problem is that with the kind of surge that we have here, we don't have all that time for that kind of counselling, so for these kinds of people is, you can even attend to just 1 out of, you know, 10 that you see in a day or 20 that you see in a day, ehen.

I: Alright, thank you, thank you so much. So, I want to know, sorry, we are almost coming to an end. I just want to know, erm, in your opinion, what factors do you think contribute to mental health challenges among persons living with HIV? What factors do you think, what are the things that contribute to our mental health problems among our patients, you know you've interacted with them one on one, so what are the things that they face, what are the problems that they feel contribute to their mental health issues?

R: one is eh, stigma. So, because of stigma they tend to hide themselves and they are, once you're not free with yourself and you're not free with the society, you're already challenged. Then another one is eh, poverty, cos poverty should include, eh, not feeling well and all that, everything that has to do with money, ehen, poverty. Then, another one is erm, I would attribute some to be genetic, maybe running in the family.

I: Okay, so in this people you have seen in our facilities, what and what factors when you interacted with them, do you notice that affected their mental health?

R: Mostly is, family issues.

I: Family issues like what?

R: Social issues like their husbands, marital challenges, their spouses, then those ones that are widows, their husband's people, like eh, the one of eh, []. The one of [] that happened, you know. This one is our patient, the girl is bad, but her uncle never wanted her mum and her siblings to remain in the family house after their father died, so the man had to beat this girl to death, mm, beat her to death, so when you are in such an environment, such a hostile environment, you know, all these social trauma, ahn ahn why won't the person, will the person be sick, the person is already sick psychologically so mentally he's sick, so the main thing is family, you know, marital issues, relations, then stigma and then now, another thing is eh poverty, no money, cos assuming you have money and your family is troubling you, you can just go elsewhere and live, ehen and cater for yourself there, yeah.

I: Thank you so much, thank you, so I want to ask, do you have any program or resources or facilities available, do you know of any program that is available or resources whereby people with mental health challenges can be managed?
R: I know of Red Aid; I know of Red Aid.

I: In our facility?
R: No, in this facility, no, no, no, no, no, none, I don't know, none.

I: Okay, okay, so, erm, I just want to ask, what do you think we can put in place for this facility to have erm, mental health eh, services?
R: Okay, if we are to have mental health services, means we have to have a resident, a doctor, a psychiatric physician that will always be on call, that will also have nurses, chain nurses in that aspect, also, that will..., the nurses will be running education, health education and also doing the counselling, mhmm, of course the doctors and nurses can collaborate or if we can have counsellors, cos counselling is key, we can have counsellors, also have counsellors that will have time, you know, to counsel these patients.

I: Alright, thank you so much, so do you have any question for me? Do you have any question for me, or what do you think? Do you have any question for me because I have come to the end of this.
R: The question I have for you is, what do you hope to achieve from....

I: With this?
R: Yes.

I: That is a good question. The-the, what I hope to achieve is to know how to improve the mental health services that our patients, eh, receive, you know, because that's why I'm sharing this with you, just to know what and what problems do you people encounter as health care workers and then how to improve our mental health, eh, services of our patients.
R: That's okay, we would be glad to see changes and improvements.

I: Exactly.
R: Alright.

I: Thank you so much.
R: Thank you.

I: Thank you
(background noise)

Transcript of key informant interview with healthcare worker 4, Facility X

I: Okay so good afternoon ma
R: Good afternoon doctor

I: My name is [] and I am here on behalf of the Epidemiology research unit of [], uhm, we are conducting this research to gain insight into the mental health needs and the factors that influence access to mental health care by women caregivers living with HIV in Enugu. Your participation is voluntarily and all information will remain confidential. I will also be using a tape recorder to ensure information is captured accurately. At any time during our conversation, please feel free to ask me any question or inform me if you will rather not answer a specific question. Uhm, may we begin the interview now?
R: Yes, go ahead

I: Alright thank you very much
R: You are welcome

I: So, I will start by asking, do you or any colleague of yours have any training, either professional training or on the job training or any experiences with diagnosing mental health challenges among person living with HIV accessing care at your health facility?
R: Yes, but uhm it's not only for diagnosing, it's a general training on mental health care

I: Okay
R: Yea

I: Okay—okay so can you explain a little bit more about the nature of this training?
R: It was a workshop organized by nurses in [name of facility] and it's for nurses on mental health care of patients, how to know when a patient is having such – or exhibiting such complaint or complaining of any problem. You watch out on a cue on how to diagnose mental health problem, how to manage depending on your capacity and then or referral as the case may be

I: Okay thank you very much
R: You are welcome

I: How—how long ago was this training?
R: I can't remember to be specific but it's not up to five years

I: Okay, alright thank you

R: You are welcome

I: So, I will like to know uhm from you, how common do you think mental health challenges are among persons living with HIV?
R: Persons living or carers? Is it—

I: Persons living
R: Persons living

I: Yes, persons living with HIV generally
R: Yes, it's common. But you know that in this our community, or in this part of the world, people hide their mental health problems or challenges a lot, and because of the stigma attached to it, but there are obvious ones. So, we have—from, let's say, about uhm, let me say 40% of our clients have mental health issues, including the young ones. So, it's somehow common but not really common—but actually, in the present condition of Nigeria, a lot of people have mental health issues. So many people are anxious, some are depressed because of the economic crunch, and then taking medication every now and then. When they remember they have HIV, they have to take their medication, they have to talk about HIV, when they want to see a doctor or a nurse anywhere, they remember it, and it keeps depressing them a lot. So those who are—who I know have obvious mental health challenges, let's say 40%. But about 80 have mental health issues.

I: Alright thank you very much
R: You are welcome

I: Have you interacted with a patient with a mental health challenge?
R: Yes, I have

I: Okay, okay what were their complaints?
R: Uhm, currently, I'm managing—I'm managing a young person living with HIV that's an adolescent that should be 19 or 19 years old. So, he forgets a lot, he complains of forgetfulness, feeling bitter, feeling depressed, wanting to kill himself, because he is the only boy. And uhm, he has failed first-line, second-line, and he is on—he is currently on third-line medication. And when he is told his medicines are not available, he tends to—he said it's like he will end his life, that it's not worth living again. He feels uhm—doesn't—all this anorexia, anger experiencing. Then sometimes, he will not eat for like 2 days. But when I uhm encourage him to take his medication but he has to take his food before the medication, he will take something, he will take maybe Sprite or take tea. So, he complains of anxiety, thinking that HIV will soon kill him and that no one loves him. Whenever he is told that the medications are not available, he said that even the health providers don't like him, that his parents hate him by giving him HIV. All those uhm irrational

talks, he is mostly irritable, he is mostly irritable. Whenever his appointment is there, it's difficult for him—he comes from Nsukka, and then we have encouraged him to seek for transfer to Nsukka so it will be nearer. He talks about stigma, in fact, he doesn't like Nsukka as he said, so a lot of negative talks coming from him. He thinks, sometimes he's sad, he doesn't smile, you have to force him to smile a little. We will equally help him to enter university so that he will meet his peers and be happier. That has been happening, it has been helpful.

I: Alright, okay. Thank you very much
R: You are welcome

I: Uhm how did you—uhm how were you able to address this uhm mental health challenges for these patients?
R: Okay yea we doing that alone. It requires multi-disciplinary care. The nurses, the doctors, the counsellors even the enhance adherence counsellors. Then we even referred him to psychological unit of [name of facility] that is psychiatric. You know they don't call it psychiatric again because of the stigma attached to psychia. So, he was referred to that unit and they have been managing him. The first time we referred him, he was afraid of that attending that place because of the stigma but we assured him that nothing like that. He has been going there and it has been helpful

I: Alright. Thank you very much
R: You are welcome

I: In your opinion, what factors are contributing to mental health challenges among person living with HIV that are accessing care at your health facility?
R: Hmm yea one of the major factors is that HIV itself and the stigma attached to HIV. It really affects them a lot. That name HIV, for the fact it doesn't have a cure, for the fact that people are running away from persons who are living with HIV, all those stigma issues. Then some psychological uhm attachment to HIV when they remember that it has no cure, and that uhm they are limited—they have limitations. For example, having marriage mate that is uhm partners and uhm these are major things, factors. Then talk about the economic aspects, the issue of funding, it affects them, there's no money in the country or that it's limited and then there are hikes in prices of commodities including transport fare, it affects them. When they come to the clinic and there's no medication, they tend to be irritable, they scold, they shout you know you keep calming them down, that it's not your—it's not the health providers fault, it is the policy makers you know issue, providing third line, its not in the country. Then talking about, you know that those on third line, for example the boy on third line, for us to place him on drugs, he has to do the phenotype test eh and so all those sending samples to Lagos because it's not done here, sensitivity test all those stuffs. So, sending sample to Lagos and the payments there are issues, there are issues. So, raising those funds, all those issues. And uhm seeing other—cos he wanted this psychological care to be rendered to him from the HIV clinic, so for the fact that he has to enter inside hospital to seek that

care is another challenge; he doesn't like going there cos when he gets there he has to talk about HIV and when they start talking about HIV psychological care, he feels that there's big burden so that burden alone is another factor. Burden of living with HIV and living with mental illness and being a young boy who is in school. He looks at his agemate, the peer pressure. When he looks at them and they are not there, he's the only one there, seeing himself alone in the matter, makes him—make it aggravated or what. You know so all these are factors that are—these are the ones I can remember now. If I remember anymore, I'll tell you.

I: Alright thank you very much ma'am
R: You're welcome

I: So, my next question would be to know uhm are you aware of any programs or resources or facilities that are available for preventing, screening and managing mental health challenges among persons living with HIV that are accessing care at your health facility?
R: Yes, I am aware

I: Okay. Are some of these programs or resources tailored specifically for women caregivers living with HIV?
R: Hmm not tailored specifically for them only. For instance, we have this psychological unit I told you about, if anyone has any issue, we send the person to the psychological unit of [name of facility] that is uhm [name of facility] is a big facility.

I: Hmm
R: Then uhm you as I have told you that uhm you know women and their hiding nature. They wouldn't want to make it obvious that they have mental health but from your – from the salient point the person is making or from the actions the person is exhibiting, you know they have uhm—so from time to time there's a program we call operation triple zero OTZ program. In as much as it was developed for young adults living with HIV, from 10 to 19 years—sorry from 10 to 24 years. We still incorporate their caregivers into the program because you can't take care of the child when you are not taking care of the person caring for them. So, from time to time when you have uhm OTZ club meeting for the adolescent and young people, we equally have uhm meeting for the caregivers and from there we note those who have any issues, mental health issues, any—. There's a woman who brings uhm an adolescent who is disabled, she doesn't walk—she doesn't walk well. She can walk but she has to be supported. This woman she doesn't find it easy, so we are always encouraging her, bring in psychosocial support for her and uhm—uhm sometime we will tell her not bring the child, she can come and pick the drugs on proxy for the child. So, we try to support in anyway we can. So that operation triple zero has been helpful. They provide small small funds that will help them psychosocially and then we have CBOs; community-based organizations who have been helpful too. They support them in income generating activities to help them in their transportation, feeding the—helping the child, feeding, all those stuffs, so these people help. And

if there's uhm big problem we can refer to the social workers in [name of facility], we incorporate so many uhm facilities and uhm agencies and bodies in managing these women taking care of our adolescents especially those who have mental health issues.

I: Alright thank you very much ma
R: You are welcome

I: So uhm have you ever used these particular program or programs you talked about uh within the health facility to help in either preventing or screening or managing mental health disorder—
R: (cuts in) Yes

I: ....in uh women caregivers living with HIV?
R: Yes, thank so much. You know that uhm that calling that say prevention is better than cure. So, we don't really want them breakdown to that uhm to mental health issues, we don't want them to break down. So, for time—the essence of that psychosocial support is to prevent all those mental health issues; irritability, anxiety, unnecessary anxiety, depression, we try to help them from all those uhm avenues, the OTZ club, the CBO stepping in, social worker from [name of facility]. So, people who have uhm-- maybe who has admission or whose wards are admitted in the hospital we try to help them come out from that shock because it's not really easy. So, we try to help in preventing it, try to help in—helping them, you know treating them. If there's any problem, we equally refer people to psychiatric hospital New Haven and we support them with some funds, and then follow up. So, we do support and if there's any uh issues within us, we always arrest it, if the one is above us, we refer

I: Alright. Okay, thank you. So uhm what specific mental health challenges have you been – do you think you've been able to either prevent or screen for or manage uh using these resources within your health facility?
R: The specific ones like anxiety. We talked about anxiety uhm and we don't really want them to come down to depression. We equally help them in – those who are depressed. Those who lose their loved ones, losing their husband, we help them through all these avenues, those who have uhm all these mental, amne— uhm what's it called sorry anxiety related to uh—cause forgetfulness what is it again? Uhm what's that name again? Borrow me the word

I: Amnesia
R: Amnesia eh Ehn! When they come to the clinic they even forget where they are. So, we always have them, we support, we have counsellors, we use counsellors too. Anywhere, anybody who can help or any resources that will be of help to them we will always use them.

I: Alright
R: It has been helpful. The social workers, they are always there in the clinic, we refer and they have been helpful in managing anxiety, depression, irritability and what not

I: Alright thank you very much. So—
R: (cuts in) you know— sorry

I: Yes, go ahead
R: I didn't mention uhm violence. You know the commonest one is violence; Gender Based Violence because of HIV. You see a woman who is thrown out of the house by the husband because she's HIV positive or some men will abandon the woman who has children cos the woman turn out to be—you know maybe when they screen the child, the child is positive so they will now screen the parents. If the woman turns out to be positive and the man turns out to be negative, the man runs. A lot of them now, so that's even what we are dealing with currently. We've had about 4 or 5 cases of violence, gender-based violence and then it causes them a lot of mental health issues because some will complain to you that they don't sleep, can't eat, they are – they have poor concentration at home, in business. Someone tell me that she was there, somebody came and took some things—some product from her, she didn't even know until somebody alerted her. So, a lot of—she was there but she wasn't there. So, this gender-based violence is another issue

I: Alright thank you very much.
R: Yea

I: So uhm I have wanted to ask how helpful these programs, you know you mentioned the OTZ program, you mentioned the CBO, who step in to provide some income generating activities for these persons living with HIV uh and the linkage to psychological medicine in the health facility. So, I wanted to know how helpful have these resources been in preventing or screening and managing mental health problems?
R: Yea absolute—

I: (cuts in) specifically among women caregivers living with HIV?
R: Yea absolute they've been helpful—they've been helpful. More of them are happy. They call on phone saying thank you—thank you, express gratitude. And you know that, that's within our capacity, we can't provide what we don't have, we can't give what we don't have. So, what we have we give and we use word of encouragement, motivation to back it up then prayers. So, if there are other ones that can be added fine and good but the ones available within our reach we will give and they have been helpful from the testimony of uhm the receivers that's the beneficiaries

I: Alright thank you so much ma

R: You're welcome

I: So uhm are—are there factors that you know make it easy for women caregivers living with HIV to access these uhm programs that are in your health facility?
R: Yea—yea factors that will make it easier

I: That make it easier
R: that will facilitate it?

I: Yes, that facilitate it
R: yea

I: That facilitates them having access to these programs
R: We have to break the factors into two, personal factors and then facility factors. Personal factors in the sense that they have to come up and talk cos if you don't say your problem, you will die in—you know what they say, if you keep—If you keep your problem to yourself, it will kill you. So first of all, factors that will facilitate is them expressing how they feel and not uhm uhm what's it called uhm

I: (inaudible speech)
R: this uhm transferring aggression no transferring aggression from the negatives, from that—exhibited by the child. So, if they can express themselves, if they can write it down even if they will not tell us, they can write it down or they can express to maybe someone around us that will help us know. Let us be on the know, let us be aware, have the knowledge that this is your problem, it will help. Then healthcare workers in the facilities, healthcare workers, having the uhm the—where with thou, having that where with thou that this maybe—must—maybe happening. They now ask questions; they should observe it first and acknowledge it because we are all human beings. If you are taking care of somebody, you bring the person today, you bring the person tomorrow; you know you have shops, you have businesses so it will affect. So, we the healthcare workers be on the know that they have these challenges and then try to ask questions about it and find out. You know the other one is subjective, the person talks about—then this one objective. We have uhm maybe tools to find out what they are passing through and it has been helpful. So there are checklists we have that we will ask them what and what, how do you feel all those stuffs, you know these are facilitators and then try to find out how to solve that problem because if you don't solve the problem of the health—the care giver, it will inadvertently affect the child the person is taking care of, it's either there is poor adherence to treatment, poor adherence to the medication itself at home, unsuppressed viral load, so it will affect all these things including the quality of life because it's not just about medicine—medicine; How happy is the child, does the child goes to school? It's when the caregiver is healthy and then have good mental health that they will now attend to the child

I: Alright thank you very much ma'am. So, on the other hand I will like to know, are there you know like barriers that make it difficult for women caregivers living with HIV to access or to have access to—to these programs in your health facility?
R: Barriers will be maybe ignorance. If they don't know that uhm there is uhm things like that maybe care or resources that will solve their problem, they will not access the care now. So, ignorance, then the provision of that care and then acceptance. If they will equally accept it okay somebody has mental health issues and you refer the person to psychological unit of [name of facility], if the person doesn't accept that he is even having a problem, the person will not go now. So, acceptance and then the psychosocial aspect, stigma, these are barriers and then the economic aspect of it. If they should pay when they get there or will it be free of charge. And the acceptance by the health care workers over there because it's not the way we do in [] that other people do. They may meet so many cadres of staff doctors, nurses, cleaners, and then somebody may make one uh comment that may throw somebody off and they may not go again. So, follow—It will affect the follow-up. All these uhm barriers, healthcare workers attitude, these are all barrier.

I: Alright
R: And even the client's attitude self. Then health seeking behavior, because if they don't seek care or you write referral for them to go and they will not go. They don't have that healthcare seeking behavior, it will—these are all barriers. Then policies, there are poor policies that doesn't really favor them, things will not really—Then another thing is waiting time. It's a barrier because sometimes when they go to the— when they come to the clinic, the waiting time in the clinic, if it is too long, they will not meet up with follow up in the clinic, they will now say they will go there another day, in fact another day they may not go till the next time they have appointment in the clinic. So, the waiting time in the clinic is a barrier, so we try to shorten the waiting time but if it's not shortened it will affect them, they will just pick their medication and go home, neglecting this mental health uhm issue and it keeps aggravating—you know mental health once you don't treat it immediately, it keeps aggravating (it keeps getting worse and worse, and it turns into another thing). Somebody that has anxiety today may come down with depression tomorrow and start talking about suicide the next time.

I: Alright thank you very much. So uhm one last question I have for you is that how can these barriers can be addressed?
R: (Pause) It can be addressed on so many levels: client level, health facility level, and policymaker level. So, from all the things I have listed, we have to segregate them into the ones clients has to solve or then the healthcare workers. So, for the clients, being aware, saying—talking it out, then saying their problem, accepting it, going when they are referred, and if there's medication, adhering to the treatment regimen is a facilitator, among others. Then for health facility, you talk about the system and the personnel, but the system—this facilitator that we are talking about—resources should be put in place. Heath facility should have their bit to collaborate with other

bodies who can render this help, because they may not do it alone; no one has everything. Collaborating with the CBOs, implementing partners, and programs. For example, for women who are pregnant, there's PMCTC that's uh Prevention of Mother-to-Child Transmission of HIV. For the young ones, we have the OTZ club, that is the adolescent club, where they can meet their peers, talk about their problems, they're screened. Then uhm for the adults, they have their support groups; they can equally enrol there. So, the facility knowing that all these uhm—all these resources exist and collaborating with them, and then the healthcare workers knowing that they are part of—they are—they are holding big stake in preventing, diagnosing, and managing this mental health illness. So, they have to look out for salient clues in clients to know when they have these mental health issues. So, to prevent them—cos I told you before, prevention is better than cure. Anything that will warrant someone coming down to mental health problem should be prevented. For instance, this waiting time I talked about, some things as waiting time you may take it as nothing, but it is big because if somebody is— stays long in the clinic, he may lose his job or her job, he may lose customers in the shop, he may leave child at school. A child that was taken to school instead of going for school run, the child remain there. (If they kidnap that child) the woman comes down with mental health issues. (If she is sacked from work) the woman—I hope you understand Igbo? Actually, the person is sacked, the person comes down with depression. So, the healthcare workers knowing all these—then attitude to work. Healthcare workers (they come first) in somehow poor attitude, talk about yourself, yourself, yourself. You don't remember that you are there for the client. Every small thing the client does, you start antagonizing them. No! You have to treat the client objectively, just manage their health, forget about their beliefs and all those things, and then manage them so that will go. For example, if you meet MSM, men who sleep with men, and uh start talking about their beliefs, start preaching the gospel, you will not render the healthcare you are there to render. And start talking about them sleeping with men, does it concern you? It doesn't concern you— So, the healthcare workers facing their work, providing it promptly. Then the system itself, the healthcare system knowing all these, harnessing and training their healthcare workers so that they will know how to prevent, diagnose, and manage mental health issues. Then the policymakers should also be aware that there are people who live with mental health issues. (Today nobody is mentally okay, even me) (laughs) So everybody is—we are all uhm exposed, or they say it's either you are infected, you are affected. So, if we are not infected, we are affected by the mental health issues, so we need to get good policy that will preserve our mental health, prevent anyone that will—the fuel issue that you know, fuel that is ₦1250 now, somebody buying such fuel, the person will be shouting (mimics) why are you? Why this price— All these policymakers, not just one, because one affects the other, you know. So, they should uhm consider the health of people. Health in the sense that the mental health, social health, physical health. All these should be considered in making any policy they are making.

I: Alright thank you very much.

R: Thank you

I: Is there any other question you will like to ask me at this point?

R: You are asking me about training, peradventure I tell you that I don’t have formal training in preventing and uhm—preventing, diagnosing and management of mental health issues, will they train me on one?

I: Yes, in fact we—I would have wanted to ask if you will be willing to you know uhm undertake such training if the opportunity arises?
R: Sure sure, it will help me in management of my client, in preventing it because my own I like prevention. I don’t like the them coming down with that – it is really exorbitant treating mental illness and it takes time to heal. Somebody whose husband abandoned to take care of 5 kids come down with a lot of mental health issues. Before she was cured, a lot of strategies were used, even if, if at all she was cured but now she is calm so I will like to (pause) undergo such training.

I: Alright thank you so very much for taking time to participate in this interview
R: Thank you

I: We have come to the end of our interview today
R: Wow thank you so much

I: Thank very much
R: I appreciate

I: We really appreciate your time and thank you
R: You are welcome

Transcript of key informant interview with healthcare worker 5, Facility X

I: I will start now, mm, so the first question is, ehn, do you have any training or experience with diagnosing mental health challenges among persons living with HIV, in your facility? Do you have any training? (any training at all). Have you had any training on mental health?
R: For mental health

I: Yes, among persons living with HIV
R: Nah

I: Eh?
R: Nah

I: No, okay, no. Then, do you? What of any of your colleagues? Is there any of your colleagues that have been trained, mental health
R: Mental health, nah

I: Services in your facility?
R: Nah

I: No, okay. So, if you've not been trained, like you said that you've not been trained, would you like to be part of a mental health training that will help in health diagnosing and treating mental health challenges among, eh-
R: Yes

I: People living with HIV, especially eh, women care givers.
R: Yes, I would love-I would love to

I: Okay, you would love to be part of it?
R: Yes, yes, I would love. I would love to be part of it

I: Biko eh, why would like to be part of it?
R: Because I have the passion to care for people of all sort on health issue especially mental issue

I: **Okay, you have the passion to care for people with mental health issues**
R: Yes

I: **Okay, thank you so much, so I want to know, how common do you think mental health challenges are among persons living with HIV. How common, in your work in this health facility, how common do you think mental health challenges is in your patients?**
R: It's, it's, it's rampant, because of eh, even though stigma link into it, that is I said it is common.

I: Then if you say it is common, have you interacted with a patient with a mental health challenge?
R: Yes, especially, when a client, don't want to-don't want to disclose her status to another eh, eh, in the- (silence) because of that non-disclosure, they cannot fluently to interact or share their views, their views and on HIV. So, because of it, except there is a, another common room where you can interact with such clients but if it's eh, this our waiting hall, mainly there are still issue on eh, disclosure is an issue and it's a mental issue, the status of that mental (inaudible sounds)

I: So, eh why do you feel that our room as in counselling somebody in that room is a problem. You said except as in like if it is our room you're using.
R: I mean eh, you cannot fluently, you cannot fluently at our waiting hall okay. Many are still shy when you talk about HIV okay, but when you go to counselling room, when you shut door, the client can listen and eh but you know disclosure is an issue

I: Okay thank you but like how many patients have you interacted with hmm that has mental challenge like how many patients have you interacted with
R: At least for yesterday, 15 clients and eh 10 have mental issue. The other one don't want to tell his husband, the other one don't want to talk about it to your (silence). They don't want to talk about it to the spouse or the partners okay. On the other side, the caregiver, they don't want to share it with the family member because once she or he supposed to have at least two caregivers so that if if when one is not at home the other one can care for the clients but there's no care giver it's because the client is still, because of no disclosure. They don't want to disclose okay. They have the battle with the mental issue

I: So, eh you said you have interacted with 10 person who have mental health challenges. So, I want to know what were their complaints, those 10 persons with mental health challenges, what were their complaints? What they complained to you?
R: Their complains were one like I said early, they don't want anybody to hear or talk about their status. The... the... the...the spouse I met yesterday, they want to do wedding today and the man said they want, they need their result before they will wed them okay. So, because of it, the lady don't want to disclose but the man want to disclose and because of it they have issue of disclosing to the man that will wed them. Because of it the lady started having that mental issue

I: What mental issues?
R: Anxiety, Anxiety uuhm, depression, how is that we stop her from doing her wedding and so on.

I: Thank you. Apart from this lady, what other complaints were the other persons?
R: Yes, the other person. Because of felt stigma. So other people, they felt that what they are discussing is about her, so because of it, instead of going to pharmacy to collect her drug. She went back because she don't want for those people that are talking about her and so she went back until she was now the last person. She went out entirely until it was only her that was remain.
I: okay so she entered the pharmacy and felt they were discussing about her
R: yes

I: So, she left the pharmacy and came back when everybody must have left the pharmacy
R: Yes. So, then the third person. She said because of the clients, the way she talked ... she talked to her (she talked to her in a harsh tone) So, she felt bad. She said if not because of HIV how will she talk to her the way she did, if not HIV okay? In fact, she started lamenting. When she is lamenting, it shows that it's guilt, guilt if not HIV.

I: Thank you. So, I just want to know those ones that you were seeing here in our clinic that complained about mental health challenges, how did you address the mental health challenges?
R: (pause) Going back, I (inaudible sound) I'll calm the patient, I'll calm the client down, then talk about it's not the end of the world, okay? so use instances, if not HIV there are other illness that is far deadlier than HIV. HIV is just an infection that if you manage it well the person will live longer and the person thought. So, mainly what people is self-stigma. What other people is saying instead of changing yourself up and seeing HIV is just like malaria. When you treat malaria, the malaria doesn't go entirely. If you don't treat it well and you didn't prevent other, the way (silence). Alright so the next thing, it is just like malaria like I said so what should you do. When you treat malaria what will do again your preventive ways do that the malaria will not come back again okay. That is how it is. The only thing is that you will be taking your medication daily so that the malaria will not come back okay, so that is how it should be. Just be social with other people but don't be your own all the time, interact with other colleagues at work, at home, so that you will free from depression, anxiety and so on.

I: Alright thank you. So, I want to know what factors do you think contribute to mental health problems in our clients in this clinic? What are the factors, what are things that cause mental health problem in our clinics, among our patients in this clinic?
R: (inaudible sounds) Okay, (what affects the mental health of clients is), firstly, when it's time to do other test, (the money becomes too much, some will tell you ₦5500, others will charge ₦7500 and they heard that treatment is free) okay and, along the line uhm finance is now coming up, it'll trigger the mental issue of that client and ehh it's not only that the client is positive, they are now how to managing it and (money is part of it, money to do test) and so on. (So, you see that it destabilizes someone. The person thought it was free, along the line the individual will still pay some money). Even though, but we tell our clients that there are other illness that are not free okay but this one because (it's been announced publicly that HIV treatment is free, along the line it's not true)

I: So apart from money, is there any other thing?
R: hmm I know, waiting time, waiting time. You know many, they are healthy not that they have other comorbidity that they have. It's just to collect their drug. At times they will stay for up two hours, at times three hours, four hours at times. So, because of it, (it becomes difficult and they become angry) (pause). You know, we will tell them that there are other process from record to

vital sign to see doctor and go to see pharmacy. But if there are other like doing lab, it takes time and so on.

I: Thank you. So, is there any family issues like when you interact with them, when you interact with them, do they complain about family things, I mean family issues that affect them mentally?
R: Yes, you know they have problem ever before they have, they know their status of HIV okay so it's now combine upon it so, it affects them in a such a way that they have issue it's not because of HIV mainly they have issue.

I: Like what type of issues?
R: Like for spouse who have not been, they as in (they don't tell themselves the truth okay). They married not on trust but because they want to marry (their age mates are getting married okay). So, but they have skeleton on their cupboard before they marry. So, when you talk about HIV, it's another uhmm (silence)

I: Okay, I want to add something, this skeleton you are talking about? I'm sorry I didn't understand it, what you meant by some of them, they are married by through lack of trust? Please help me explain better by what you meant by that?
R: So, because of that skeleton on the board, so you see that the man doesn't give money for feeding, okay? At times they are on... (drink excessive alcohol) so when he comes back (you know someone that drinks excessive alcohol), even on the bed cannot do much okay? So (the man or woman goes out to have an affair and contract any infection), it will infect the spouse okay. Eh ehn or that the man is infected and looking for other avenue to infect the partner I mean the wife okay. So, there are other skeletons like they have, they cannot tell them (silence). So, because when the man have more, other sexual partner and when the wife now know about it, it (it affects the mental health of the lady because she will be disturbed in her thoughts)

I: Okay thank you
R: And even (it will make the medicine, HIV medicine to not work effectively because she will be disturbed mentally)

I: Alright thank you, thank you so much. Are you aware of any program, any program or facility that is available for screening and treating of this mental health challenges in your facility? Are you aware? Do you know of any program or services that are in charge of treating mental health problems among people living with HIV in your clinic?
R: In my clinic? For now, no

I: Okay you are not aware of any program?
R: No

I: Okay
R: Ehh, there was a time we tried using questionnaire to help if there are issue about mental health issue status of clients but along the line (sighs) I in particular, I stopped

I: Okay but are there any program or facility that are in charge of mental health problems for these people living with HIV that you know about?
R: No

I: Alright thank you. So, do you have any question for us?
R: You said question

I: Yes, do you have anything you want to say or contribute or ask me, anything?
R: What I want to say is now that we are talking about it. It is good if we start towards that end of having mental services in our.... in our HIV clinic specially. I would be glad if we have one here okay. At least if with, like I said earlier, a template that can help those question that will come up that help to see that our clients, our health mental is stable

I: Alright thank you so much. Do you still want to contribute?
R: (silence)

I: Do you have any other question?
R: Not a question but like is said, I'm eager to hear that in our clinic, here in [name of facility] we will have one of (pause) (we will have) mental services in our... in our (silence)

I: In our clinic?
R: (inaudible response)

I: Alright thank so much. Thank you, for your time

Transcript of key informant interview with healthcare worker 6, Facility X

I: So, I will start with asking some few questions about mental health? So, the first one is do you have any training? or experience with diagnosing mental health challenges among people living with HIV in this facility? Do you have any training, have you been part of any training?
R: I have gone – I have gone for trainings from NEP 1, CCRM, Caritas but particularly on mental health

I: But they added mental health in the training?
R: In the –Yea, I am a gender-based activist, local person here. So mental health is part of what I do give out, counsel patients

I: What of your colleagues, do you know of any of them that have been trained?
R: On mental health?

I: On mental health
R: I would not say because I don't know if I have heard of any training that is captioned mental health

I: okay what you are saying that's there's no training that been captioned mental health training?
R: Yes –yes – yes— yes

I: The one you attended was in addition to other –.
R: Other things

I: Okay
R: You know our work is basically on counselling, so it's part of it

I: Okay. So how long ago was the training?
R: More than three years now.

I: More than three years?
R: Hmm

I: Are you having—(interrupted)
R: Because since caritas came out, they have been sending only their staff, adhoc staffs. They don't normally call us for training again.

I: Okay
R: Like now they are on training. One of us, Eva is there but none of us went

I: What training are they –?

R: I don't even know when she came, because she will step down for us, they will step for us. I don't know the training.

I: So, the last one was three years ago
R: If I could remember because I'm not with the certificate now

I: So how common do you think mental health challenges are, among persons living with HIV? You work here in this facility, so we just want to know how common, the patients you interact with? How common do you think mental health challenges are, among our patients?
R: It's very, very common because HIV is one sickness they see as death sentence, and not even death sentence, that anybody that is infected with HIV is a wayward person. That is why it is very-very common, let me cite an example now. There was one, one lady was here yesterday. So, she was diagnosed, we did the test and it was confirmed that, she's positive. She was just remembering what people will say, what they have saying about people in the village, in town, that it's better you have another— if it is cancer, it's better than HIV and came out from almost all the patients that comes here. Until you talk them out of it, they see HIV as the worst sickness ever because of the stigma and the tag they have been giving it in the past. You know even the caption they gave HIV then where they draw skeleton eh ehn, so HIV is very very— mental health is very-very common in people living in HIV but as a counselor we try our best to talk them out from that notion

I: So, have you interacted with a patient that has mental health problems in this facility? Have you interacted with any patient that has mental health challenges
R: Of course,

I: Like how many patients, like you can you give--. (interrupted)
R: I can't count. I have worked here for more than ten years so I can't count

I: But there are lots of them
R: Yes, but with time, with time, constant counselling and all that, they used to drop-drop it

I: What were their complaints?
R: Complaints— Stigma is the major complaint. Stigma, how people will see them. Maybe the husband is positive and the wife is negative. One even now, up till, for fifteen years have not told the wife why? That the wife is a nagging wife so, so, so that he cannot open his mouth and tell the wife. So many of them

I: Apart from stigma, what are the other complaints, like those people you have interacted with, what are the other things that they (interrupted)
R: You know stigma is the major, why I said that stigma is the major problem is, most of them okay, maybe you are doing a bakery and somebody came to know that you are HIV positive and

spread it across, it will stop your business. So, it’s that stigma leads to so many things and it will make them, some people will not want to stand up again and do something for themselves, they will think their life has ended.

I: Okay thank you. As a healthcare worker, how did you address the mental health problems, the ones you interacted with, how were you able solve or address the mental health challenges?
R: As I have said before that counselling, giving them reasons to live, reasons to see HIV as— that HIV is not a barrier to anything that you want to do. It’s your— it’s your brain—what do I say (laughs) It’s how you see HIV but when you drop it and drop whatever the people are saying about you, just live your life. Eat very well, find something doing. The important thing is finding something doing. Don’t be a beggar because when you lean on somebody, the person might stand up anytime and tell you that you don’t have, I don’t have to give you. It’s better you find something doing and equip yourself so that you will stand up to yourself. Tomorrow if anybody comes around you to talk rubbish, you face the person. So, the mental health issue depends on you, how you take it.

I: This one you are mentioning about them finding something to do? Does it mean that (interrupted)
R: (answers phone call and continues) Finding something doing, why I said finding something doing, most of them lost their job during-- some even dropped that they don’t have the strength to continue again since they are now infected that they don’t want to carry on. You know when you meet with such people, counselling—

I: Does it affect their mental health
R: Of course, now. Mental health is it not when you are not stable eh ehn. Something must have led to that, that will make you not to be stable.

I: No, what I want to know is, those people that said they have stopped doing something, their work, and they are not doing anything
R: eh ehn?

I: Does it affect their mental health, not doing anything?
R: As in, their mental— I don’t know what you mean now by the mental— is it somebody that have challenges—In their ways of – their mentality, or their—?

I: No.
R: Eh ehn

I: What I meant is you know, just normal, somebody that maybe being sad, being depressed, being everything. That’s what I’m asking
R: of course! When people are stigmatizing you, maybe you are cooking and they went on and spread that you are HIV positive, people will not stop attending your buffet or bukka, your mama put. It’ll affect your mental health.

I: So let me go back to that question I asked. I asked whether-- you said something about telling them to find something to do. I just to know, if they are not doing anything, from your own perspective, do you think it affect their mental health. You said one of the counselling you give them--
R: eh ehn, eh ehn. Of course, because so many of them, you know most of them, they are not literate. Some that are literate are working, maybe you are a government worker and all that but if you are a business person, your ways of life, when people started stigmatizing you will drop. Like one woman, I have an example, she is living at []. What made her swear not to come here again is because somebody came here and saw here, a patient, and went to their place, she is selling vegetables. The person went to their place and told people that this woman is HIV positive. People started talking it and they stopped patronizing her. Won't it affect her mental health? So, this woman vowed not to come here, I was the one that kept on-- the husband called me, I kept on counselling, doing all sorts of things, even went to her house because we wanted to take the case up. The husband said no that he don't want case. But now she has started coming.

I: I also want to ask apart from this stigma that you said that it's their major complaint, do some of them feel bad, do some of them feel isolated? Do some of them feel isolated? So, I just want to know, what other complaint that ehh with those you've interacted with?
R: Because of their mood it could lead them not to attend meetings like in women, to attend meeting, if men-- like most men that are not-- that are drunkard before. Being HIV-- Mental health will lead you not -- because you've been told not to drink again but going to that place is useless for you so it'll make them to seat at home and be sad because they said, when you drink you drink out their sorrow but since they are not going there, they will be there and be sad because of HIV

I: Okay. Can you remember any other complaint that the patients you've interacted with complain about?
R: Okay, okay. Some patients will tell you that where they are working, they have sent them away because of HIV because they will ask them to go and do screening, HIV screening. Some people will even tell you that they wanted to ermm write a letter to work maybe they want to be employed in some places, they will ask them to go and bring their HIV screening result. Because they are positive, there's no way they can take that positive result to them. It will make them to be sad

I: Okay thank you, thank you so much. I just want to know, in all these complaints that patients are complaining, how were you able to address the mental health issues?
R: It still boils down to counselling because there's nothing you will tell them. You will not take the HIV out from somebody right? But you counsel the person to be alive that tomorrow will be better. Just keep on pushing it, take your drug judiciously, whatever comes up you will see it but be alive first and see if you are taking HIV medication, you will be healthy. You will even be healthier than somebody that is negative. So, it will boils down— it boils down to that counselling

I: Okay thank you. I just want to know, I know you've mentioned some things about some things that can cause mental issues but do you have any other thing you will want to say

that can cause, factors that can lead to mental health issues among our clients in this facility
R: What could lead them to that is maybe—let me just cite an example of coming to the clinic—coming to the clinic. Somebody woke up in the morning very early, came down to this place to access treatment. Coming to this place, maybe the person got here seven, eight, nine, ten, eleven and he or she has somewhere he's rushing to. You've not attended to him or her, that could lead to (inaudible sounds) That could lead to mental health and when you talk to— when staffs talk to patients arrogantly it could lead to— we are human being, it could lead to mental health because the person will just felt it is because of this sickness that you're talking to me in that manner

I: Apart from our health facility issues, is there any other issues outside that (interrupted)
R: Issues outside

I: That can trigger mental health problems
R: In a society, where maybe, but I have not seen anything that will make somebody maybe, where you are selling, you cannot sell because of HIV, maybe you wanted to go for church program because of HIV you will not attend. Only when you are stigmatized if they know. That is reason why they are hiding it. Because when you come into a church nobody--. they can't write on your face unless you are down. But when people know and stigmatize you, it can lead to mental health. The stigma--.

I: Okay thank you. You said stigma, what of in their family, is there anything that (interrupted)
R: Stigma! That stigma. Because one woman now throw her daughter out. The daughter is not sleeping with them inside house because she find out that the daughter is positive. Can such thing--can't it lead to mental health? The mother that gave birth to you or a daughter-- or a daughter that is stigmatizing the mother can't it lead to a mental health. Okay, husband and wife, you find out your husband is positive and you went about telling everybody. Can't the husband be emotionally down? Abi? Eh ehn! We have so many things that-- all I'm saying is that, that stigma is the center of it because they don't see HIV as other sickness, we are still saying. A cancer patient can come out openly and say I am suffering but HIV cannot. Even when you are so healthy, you cannot. Because of the-- I don't know, should I call it awareness is not strong or what in this part of the world

I: Alright thank you, thank you so much
R: or— or— the way they see HIV because unprotected sex is the major 80% of the carrier. So, they see the person as the harlot if you are a woman, man, ashawo

I: Alright thank you so much. Thank you. Are you aware of any program or facility available within this health-- HIV clinic that offer mental health services for our HIV client?
R: This [name of facility]?

I: This facility, yes yes
R: This [name of facility] or this our [] clinic

I: Let's start with our [] clinic. Are you aware of any mental health services in this our HIV clinic
R: Apart from the ones we are giving? We are the counselors

I: Yes, apart from the counselling, are you aware of any mental health services in this HIV clinic
R: I don't-- I don't know. I have not heard of it

I: What of this health facility? Are you aware (interrupted)
R: You mean [name of facility]?

I: Yes, yes [name of facility], are you aware of any program?
R: There could be but I don't know

I: You don't know. Okay, thank you
R: Cos all we need we do it here, we counsel our patients here so we don't know of any other mental health issue or facility-- clinic. But do we have? Can I ask that question. Do we have?

I: I think. I don't know, I'm asking but
R: You don't even know

I: Okay but I want to ask you. Since the one you said you, people do some counselling here. Do you screen people to know whether they have mental health issues?
R: No

I: Do you screen them? With like a questionnaire or something to assess whether they have mental health issues?
R: No, we don't have a questionnaire. It's when you are interacting with the patients you know. What the patient is telling you will make you to know.

I: That the patient—
R: The patient can even come to you to narrate his or her problem. So, you know he or she is passing though mental health issue

I: And then you do what?
R: I will intervene with psychosocial support

I: Please can you tell me some of the support again
R: Support, major one is that counselling, if there's anyway. If it is financial part of it that I can help because most of them can lead to that. If it is financial, if I have the capacity, I can help or if I don't have, we refer them like OVC. Like ones that was telling me that they don't have school fees for their children then you refer them if you don't have

I: Okay thank you. Thank you so much. So, hmmm, I think we are almost rounding up our discussion. So, we are almost at the end of the discussion. From what I gathered, you said you don't really have a program in this facility for mental health services. Okay, thank you

for your time. I just want to know; do you have any question or comment? Anything you want to contribute to our discussion
R: What I want to contribute is creating awareness. We still need strong, strong awareness about HIV. To change how people see HIV communities and all that. We still need strong awareness.

I: And how do you think we can carry out this awareness
R: Eh ehn now we can meet the facilities where they run HIV. NACA, you can-- you people can go to NACA, is it NACA or ENSACA Enugu state eh ehn. Enugu state is Enugu. Then national, eh ehn so you can engage them or we will do it. We do awareness here. We go for vocal hmm OVC--. what do I call it

I: Outreach?
R: Outreaches. We go outreaches so we talk to communities. The ones we can do. Like this august meeting, I know how many we did

I: So, you want us to create awareness program
R: Awareness program yes

I: On what now?
R: On stigma against people living with HIV

I: Okay, do you think bringing mental health services in this clinic will help in reducing this stigma?
R: Of course, uhm no I don't think

I: Why, why don't you think?
R: No-no what it will help is just that our patients stand up to their feet. Nobody will be able to pull you down cos you are --. but that one apart. But when you go to (laughs) the villages, those that are not coming here, maybe he's stigmatized here, maybe he's not attending [name of facility] clinic, they will stigmatize the person there. They will always want—okay one man died last week that what is—that his problem is poison. Actually, it was HIV that killed the man. When you mentioned HIV, they will not agree, they will say it's not their portion, they will rather. If the person is down, they will rather--. if the person is down, they will rather go to a native doctor, the person is passing through poison that he ate poison but the person is positive but when you mention that HIV, they don't want to hear it

I: Alright. Thank you so much for your time. Thank you.

Transcript of key informant interview with healthcare worker 7, Facility Y

I: So, Good morning ma.
R: Good morning

I: My name is [] and I am here on behalf of the Epidemiology research unit of [name of facility] Enugu. We are conducting this research to gain insight into the mental health needs and factors influencing access to mental health care by women caregivers living with HIV in Enugu. Uhm this interview will last for about 20-25 minutes and your participation is voluntarily and all information will remain confidential. I will be using this tape recorder to ensure information is captured accurately. But at any time during our conversation, please feel free to ask me any questions or inform me if you will rather not answer a specific question. So, at this time, do you want to ask me any question about the interview?
R: Continue

I: Alright so may we begin the interview ma? Okay so thank you very much ma for having us. Uhm my first question is; do you or any colleague of yours have any training? Whether it's professional training or on the job training? Or experiences in the course of your work with diagnosing mental health challenges among persons living with HIV that are accessing care at your facility?
R: No special training. But as a doctor I can make diagnosis

I: Alright ma. Uhm so ma'am uhm would you like or will you be willing to be part of any formal mental health training in diagnosing and managing mental health challenges among these group of clients if organized in the future in your facility?
R: Yes of course. I will want to

I: Okay ma. May I know why you will like to be trained in such?
R: To help in the client management. If I have any special training it will help.

I: Alright ma. I will like to know from your experiences, how common do you think mental health challenges are among persons living with HIV?
R: It is common but quite under diagnosed but it's common. It is.

I: Have you interacted with a patient with a mental health challenge in the course of your providing care in your health facility here?
R: Yes, I have

I: Okay, can you give us maybe one or two instances....
R: Hmm

I: That you can recall?
R: The one I can recall is that uhm a client that came in. They're a serodiscordant couple. She positive while the husband is negative. Initially she was hiding, because she was hiding, she didn't want to disclose it. We kept counselling for us to persuade she didn't but later she succumbed and disclosed to the husband and eh the man changed into.... the woman became .... she was so depressed actually from that period. It was so bad and now we don't even see her anymore. We have kept calling, to get her you can't. Even when we tried the trackers, maybe to go her home, the address she gave us was false so we couldn't get her. But after that episode, disclosing to the man, we don't see the woman and because we noticed changes in her. She started even losing weight, a lot of things before she stopped coming and we don't even know anything about her. Sometimes even, usually it is mostly in serodiscordant couples that's where we see them. If both of them are maybe positive, they are all taking their drugs, usually you don't see it. It's common in serodiscordant couples.

I: Okay, thank you very much ma. So uhm in some of the other interactions you've had with this category of clients, with mental health challenges, can you tell us a bit more about some of the complaints that you can remember they presented with, that prompted you to think along the lines of mental health issues?
R: They always complain of crawling sensation all over their body. There was a one.... okay hallucination (silence). Mostly hallucination and that burning sensation. These are the common ones I can remember

I: Okay ma. So can you maybe take us through, just briefly how you addressed some of these challenges in some of the patients or clients that you saw?
R: Some I had to refer to a psychology and psychiatric unit. This is the commonest one that I want to assess

I: Alright. So, ma in your opinion, what factors are contributing to mental health challenges amongst persons living with HIV that are accessing care at this facility?
R: Stigma. Stigma is the commonest thing that causes it. (silence) In short, the commonest. What else can I say causes it? (silence) Stigma part of, maybe the husband, the relations

I: You know earlier on you also mentioned discordant couples
R: eh ehn so it's the same thing. When they're discordant, the man will be treating or the woman will be treating the man somehow. Those are the commonest causes, apart from stigma, especially in discordant. Even concordant couple, they may get it from the relation or even neighbors and everything. Some of our clients, once they come and see somebody they know

around, they will disappear you won't see them again. Stigma... stigma... stigma. Those are the commonest thing. Apart from stigma and knowing that maybe I'm sick oo and I will soon die, maybe, these are the main things.

I: Alright ma
R: When they are talked too and told that this is not death sentence that you can live quality life once you take your drugs. Because there are clients, when I came in... was diagnosed about fifteen.... they are still doing well, more than ten years ago.

I: Thank you very much ma, so that leads me to the next question I will like to get your insights on. Are there any program or resources or facilities that are available you know for preventing, screening, and managing mental health challenges among people living HIV, that are accessing care at this health facility?
R: I didn't get that question very well

I: Okay, are you-- okay let me say, are there program or resources or facilities you know, are there things, health related program or services that are available in this health facilities for the purpose of helping to prevent mental health challenges among people living with HIV, that are coming here to access care or screening them for mental health challenges or helping with their management that you are aware of?
R: There's no special program. The only thing I know is that we have like their support group meetings that help them but not specifically to mental health

I: Okay
R: But they-- even the last, about last month, they even organized funfair but it's for the children, adolescents. These are things but there's no special program for mental health. No.

I: Okay... okay. So, you mentioned about you know support group that helps with you know addressing some of these issues that persons living HIV may have and that uhm the last meeting that they did, they did something like a funfair but for adolescents? Can you remember or recall any such or you know uhm time that that support group also organized something specifically for maybe women living with HIV or women that are caregivers that are living with HIV?
R: It's not only for women, it's all of them.

I: Okay
R: They don't organize program for women.

I: Okay
R: But they have support group meeting every month

I: Okay
R: It's done every month. But on special occasion maybe Christmas, they will organize their Christmas party and the funfair they organize is by Caritas that is IP that helps this one organize it. It's for all people in Enugu, they came. It was done in this hotel what's it called again? In eh Oakland. They organize things like that but not specifically for women but for all. They have their adult and their adolescent group.

I: Alright ma. Thank you very much ma. Uhm are there other programs uhm or resources like the one you mentioned that you think from your experience working with these group of clients can be made available for screening for mental health challenges or preventing it or even managing it when it has already occurred among these group of clients?
R: It will be good if there such program is floated. It will be for them because a lot of them have mental issues, a lot of them.

I: Okay thank you very much ma. So, ma my next question is uhm, have you ever had the need to you know maybe refer your clients to that support group because you think that they benefit from it?
R: All our clients, we refer them to support group. It's either you go to adolescents or adults.

I: Okay, okay. Alright (silence). So ma, are there you know factors that you think make it easier for women living with HIV, particularly those who are care givers to access some of these program? For example, the support group that you mentioned uhm that would be helpful in you know taking care of their mental health need while accessing treatment here?
R: Eh yes. There is this OVC group and they help them. Sometimes they support them with transport fare to and fro. They even visit their groups; some they even help them access drugs and give to them. There-- there are groups that help. There is the OVC, apart from the support group, especially mothers maybe that have children that are positive so they help them. That is the vulnerable children.

I: Okay, alright. Thank you very much ma. Are there-- are there barriers, you know factors that make it challenging or difficult for these mothers or women living with HIV to access some of these programs? Even though they are willing but they are constrained because of some barriers? Do you think there are such barriers?
R: Barriers?

I: You mentioned that they--some of the organizers of the support group meetings sometimes provide them with transportation
R: (cuts in) They do, especially vulnerable children

I: Hmm
R: ...they provide them with support

I: Apparently to help limit the problem of you know cost of transportation
R: (cuts in) Because sometimes you call them, they say they don't have transport money. They give them

I: So are there other barriers that you...
R: (interrupts) they even help the mothers. Some of them, they help them financially. If they want to open maybe a business, they give them. They support them. So--

I: So, apart from that economic barrier that these support groups, or the organizers of the support groups are trying to help them surmount, are there other uhm barriers? I know you mentioned stigma earlier on as an important factor
R: Ehm that's why the meetings are held on Saturdays when there's no clinic day. They won't see people the know and all that

I: Okay
R: That's when we have the meeting and when we have the meetings, we've tried to incorporate other people so that you won't know it's just HIV patients.

I: So, ma apart from that economic transportation issues and uhm stigma, are there any other barriers that may be coming to your minds at this point?
R: That will be prevent them from accessing maybe coming to support group?

I: Or coming for any other mental health services that may be-- may become available?
R: Except if the clinic... if it's clinic. Eh ehn if the... maybe if it's mother, the husband have children accessing drug, they may not be part of the OVC, transport is usually the problem, transport fare because of the economic situation in the country.

I: Yea
R: It's affecting even our... eh we now have a lot of IIT that is interruption in treatment because of that

I: Hmm
R: (Inaudible speech) The vulnerable children, those are the people they have provision for. So as you are helping the children, the mother. But if they don't have any positive child, they don't give them, they don't support them. So those people they have issues to access drugs.

I: Alright thank you very much ma. So, ma how can some of these barriers be addressed in your own opinion? How best do you think some of these barriers can be addressed? So that more people living with HIV can come and access whatever mental health services that are made available for them?
R: One of the barrier I see is training. We need to be trained (inaudible speech). So that you may-- you may-- okay you pick the signs at a very early stage. Because if you are not trained you may not pick it-- ehh one... so training is important. Two, but if I talk of transport (laughs) I...I...I...

I: (Unintelligible speech)
R: Eh ehn their transport that's helping them economically at least so that they'll be able to access. The problem, what they usually complain, most of them when they miss appointment and you call them, it is usually transport fare that is their problem. Usually-- usually, that's why they miss their appointments. And eh well counselling. If they are counselled very well, they will know the importance

I: Alright ma so uhm thank you very much ma'am. At this point, is there any other question you will like to ask me?
(silence)
R: You said this research, it will help you in policy making?

I: Exactly or provide interventions
R: Interventions

I: That can address the gaps regarding mental health
R: (cuts in) the only thing I...I... I want to say is, I hope this will be used to

I: We hope so too
R: Eh ehn that's the only thing.

I: Yea
R: Apart from that, it's a welcome development. At least doing all these researches. I hope it helps us at the long run (laughs)

I: (laughs) we hope so too. Thank you, ma

Transcript of key informant interview with healthcare worker 8, Facility Y

I: The first question is do you or any other colleagues of yours have any training, or professional training, or a high job training or experience with distinguish—with diagnosing medical health challenges among persons living with HIV—among persons living with HIV accessing healthcare in your facility? Have you had any training or anything on mental health before?
R: Hmm No

I: No okay that's question 1. Then question 1b says okay if yes—okay then there's no need since you said you've not had any training. We have to skip that question 2—question 1b. So, we now have to go to question 2. How common do you think mental health challenges are, among persons living with HIV?
R: Very very common. More common than we know cos when you get to interact with them, you find out most of their symptoms, when they come out to tell you—most of their concerns are basically mental health issue and it is a very core and important, vital uhm aspect of uhm care that should be offered as in that should be looked for—looked into—

I: Yes
R: Aspects that uhm has not been addressed properly in the management of persons living with HIV in my healthcare facility

I: Okay
R: Hmm

I: Okay—okay. That's question 2. Question 2b, have you interacted with patients with mental health challenges before?
R: (cuts in) of course

I: ... that come to access health care in your facility?
R: Of course I have

I: So how did you address their mental health challenges?
R: Hmm, it—it was not really an easy one. There's this particular one, like uhm, she came up that there's this symptom, 'I feel there's this crawling, each time I take this medication, there's this crawling sensation I feel. It's as if I'm fainting, it's as if my uhm—my—my tummy is bloated. In fact, I don't get myself, and at night I keep dreaming. And uhm, in fact, after taking the medications, when I come out in the street, I find out my street people laugh at me.' So, it seems like you're talking to somebody that there's a disconnect somewhere, ehn ehn. So, there was no

correlation with our drugs and the symptoms the client is depicting, so—so I knew it was tending towards mental health issues. And in her own case, we had to look into her medication to find out if any of what she eats at that moment had the side effect, like and we visited and uhm, not really, because assuming she was on TLD and we—we basically look out for these kinds of symptoms, like hallucinations and all that for people in TLE, that is efavirenz-based regimen, but the client was on the other one. And we just saw that it was more of the uhm—uhm health uhm, will I say, the health conditions, like the patients being on medication and just tagged HIV is a major problem. It's just giving patient's symptoms that are in line with uhm, mental health. And I think that is it.

I: So how did you people address that issue, like for this lady you are talking about?
R: Yes. We got uhm— in my facility there's something we call client-referral form, cos it's a multi-disciplinary approach we usually do. When we see, we can't get to that point, we call for help, that's bringing in, writing a referral letter to the psychiatric colleague-client, so the patient was actually referred and presently, the patient is on anti-depressants and his condition—her condition presently is fair and she has come back to care and not—she doesn't complain of such symptoms again. That's how we addressed it

I: Okay thank you so much. Question 3. In your opinion what factors are contributing to mental health challenges among persons living with HIV accessing care in your facili— health facility?
R: For me uhm, I think the number one is uhm socio-economic factor. Right now, it's just the high standard of living, a lot of patients are peasants, farmers, to traders that actually their income it's so low and the whole thing about money gets to affect them. So, secondly, you see a client, most of our client suffer from stigmatization, in the sense that, you know being in a teaching hospital, citing of our clinic has uhm, it's like a two-storey building and have other specialties. So maybe the client might be climbing up the staircase, he sees a cousin or a neighbor you know we have seen that severally, so she goes into your office and tell you please is there someone that can bring down my drugs for you. So, you see that stigmatization is a very—so that kind of patient is already like lost to follow up you know, you can't -- has-- already have some interruption in treatment because when that client knows I coming, I might see someone I know, the patient is like I don't think I want to continue with this medication. Another factor could be not having the right—as in not being uhm pre-informed, counselled, thorough counsel— Most times uhm when a client coming in, the client needs counselling at each table, from the first person that sees him to the last person, keep telling the importance of taking his or her antiretroviral therapy. Then another factor I will say is uhm pill burden, but this one is more peculiar to children, having so many drugs to take. And also, adults too, because we have adults that have comorbidities like DM, hypertension, having to combine, drug-drug interaction, all these uhm, it can be very distressing. So, they come up with questions like "doctor when will this – when will I stop taking drugs" "Is there now a cure" "are there

injectables". These are options they want to hear from you, so all that you know that—in one way or the other trying to have some mental health issues concerning their conditions.

I: Okay, thank you so much. Okay let's go to question number 4. Are there any programs, resource or faci— uhm facilities, available for preventing or managing mental health challenges among persons living with HIV accessing care in your health facility? Are there any programs currently going on? In terms of the mental health aspect of persons, patients accessing care in your facility?
R: I don't think there is basically a program I'll say, apart from the – at times there's what we call the national echo that has to do with uhm national echo. It's like a-a-a program is uh like a-a-a topic, I have seen uhm in some cases that mental health issue was addressed like somebody come to talk about mental health but most times uh-uh clients are not eligible to attend such programs. So, basically, for our facility, when you have someone living with HIV, the patient has to also be together with other clients that have mental cases. There is no segregation, they are all treated together. So, there's no— I will say there's no specific program

I: For them
R: For them yes. There's no specific program

I: Okay thank you. Question number 4a. Are you aware of any programs, resource or facilities available for prevention, screening and managing mental health challenges among persons living with HIV accessing care in your health facility?
R: No—no one I know of, no

I: None at all
R: Yea

I: Okay thank you. So, let's go to question 4b. The one we just answered is 4a. Question number 4b. How helpful have these programs, resources or facilities been in preventing, screening and managing mental healthcare problems among women caregivers living with HIV accessing care in your health facility. What I'm trying to say is that if those programs are available, how are—are they really helpful?
R: Like I said I wish the clients too will have opportunity to attend the programs. I'm sure it will help a long way, when they get to listen to experts talk to them about the dangers of uhm—trying to key in themselves into having mental issues. It will go a long way to help manage women—

I**: (cuts in) especially among women caregivers living with HIV**
R: Yes- yes- yes

I: Okay thank you. So, there are none for now but if it's made available it's going to be very helpful?
R: Very—very helpful

I: Okay. Question number 5a. what are the factors that make it easy for women caregivers living with HIV to access this program, resources or facilities in your healthcare facility?
R: Hmm number one I will talk about if uhm they will more educated

I: Okay?
R: So, try to have uhm-uhm they will know the importance of uhm what the issue is all about. So, in all it will help them address such challenges when it comes to them. Then having proper information. Uhm if uhm these mental health uhm issues can be talked more often, you know clients can be encouraged, mothers can be encouraged to always speak out when they feel the burden is too heavy on them and seek the uhm seek to talk to a healthcare worker, be it a nurse, or uhm a doctor. So, in that way I'm sure it's going to help. It's just in all if the literacy level of our mothers would be you know like even if it is average, a bit above normal, you know it's going to help a long way, that when you have educated a mother, you've educated a nation. In that way they could spread to their children, they could spread to their spouse, and it will help them manage their homes properly and adhere to their medications and they don't fall into the dangers of mental health

I: Okay apart from the illiteracy level and all that, is there any other factor? You know you mentioned something about the location of a clinic?
R: Yes—yes

I: Don't you feel it's going to help too
R: (cuts in) Yes—yes –yes

I: If they are near
R: Yes—yes – if they are somehow secluded like you don't get to tag—whenever you know someone is going up there, the person is HIV patient and it will also help to curb the issues of stigmatization to the barest minimum. It is going to help a lot. Then uhm if mothers too, you can organize more programs for mothers like maybe every other day. Just call them together, mothers come together, women of childbearing age, they come together, they pour out their minds. Uhm they are given sort of – they are motivated and you maybe give them some light refreshment or uhm –

I: (cuts in) stipends
R: Yes stipends. It's going help in a long way and it'll motivate them so much and they will feel loved and the dangers of diving into mental health issues will be reduced

I: Thank you so much. So, question number 5b. what are the obstacles or barrier in bracket factors that make it difficult or challenging, limiting women caregivers living with HIV to access this program, resources or facility in your health facility?
R: Hmm for me number one is the government policies. If they can – government can be met to address uhm the health policies then if a bill can be brought up by the higher authorities concerning—centered on women with mental issue, it is going to—if a bill can be brought into. Then second uhm the—

I: (interrupts) We are talking about what will make it difficult for them to access this care, that's barrier
R: Okay barriers okay

I: Yes obstacles
R: uhm—uhm number one most times, like I told you illiteracy is a very key – in this thing. Most of them having to leave uhm what they are doing; they cherish it so much. Some of them are farmers, and it could be time of harvesting so it's going to be a barrier. Whatever time of the year, whether it's rainy season, they are in their farms, dry seasons they are harvesting uhm around this time they are eh ehn so it's going to— Then most of them too, we found out that most of them from the statistics, most of them come from very far areas, coming from [], as far as that. They want somewhere far from where they stay because of sti—the issue of stigmatization and so it's also going to be a barrier. Then high cost of living, transportation, they hardly come to clinic. So, most of them even already with mental health issue, they get to deteriorate because they don't come around to talk, to share and it gets the matter worse. So, all this I feel—poverty, all are still coined around socioeconomic factors in being there. So, it's going to be stumbling block to achieve this.

I: Okay thank so much. So, number 5c, question 5c. How can this barriers or obstacles be addressed? What do you think we should do to able to address this barrier?
R: it's going to be very—uh its going to be very difficult I would say but, in a way, it can be very doable. Uhm for uhm in terms of high transportation cost uhm even if uhm there will be advocacies like people that will – volunteers, that would you know, already in the HIV programs there are things that we call the differentiated service delivery whereby clients with uhm common or peculiar home addresses, they all gather their drugs and take it there and once you are virally suppressed and eligible, they take it to your doorpost and they tell you come out to this place. Maybe before such drugs are given, a sensitization program can be given as – like to address the mothers, tell them, please health talk as part of—you will also talk about mental health in that way too, you can also talk to other mothers and all that. I think it's going to help with that aspect. In the aspect of uhm moving, it's now in the hands of the authority trying to

move the clinic like I said it's going to be very difficult but it is possible. So, we hope that maybe if more offices are made, they could—they could be a decongestion and the uhm the HIV services will just be in a secluded area and issues about stigmatization will be addressed. So, these I think are the ways the barriers could be addressed

I: Okay thank you so very much. We really—really appreciate, I appreciate. Thank you for making out time to participate in this interview. Is there any other question you will like to ask?
R: (pause) it is going to be a very good one if looked properly into because not only our women even the men too, even –in fact in generally, even it has gone out to our children too. They come up with uhm questions, that is people living with HIV, they come up with questions "mummy when will I stop—what's this thing I'm always taking" you know and all that. So, you see the child is already like affecting the child's performance in school, having to come out to pick his/her drugs, having to have his or her viral load taken. So, I really commend the organizers and uhm I wish and I hope after this, more will be driven like if it—there could be an expansion beyond the women also, make it generally for—but it is good to take a small step at a time but I'll say uhm well done and kudos. Thank you

I: Thank you so much. I'm going to end this now. Thanks a lot

Transcript of key informant interview with healthcare worker 9, Facility Y

I: Do you eh, do you or any of your colleague have any training or experience with diagnosing mental health challenges among people living with HIV in your clinic? Do you, do you or any of your colleagues have any experience or training on mental health among eh people living with HIV-HIV, in this clinic?
R: Well, we have not gone to any clinic of mental health, but what we are having here is experience of telopathy of a patient, if the HIV will enter, if there are... the patient did not receive drugs early and the HIV will now go into his... the patient will now be talking coherently, will not be talking normal, will be talking mentally, erm, mentally, something like that

I: Please continue, (We are talking about training or experience on mental health)?
R: (speaks Igbo: So, we have not gone for any training or have any experience on mental health. But concerning HIV, we have not gotten people with mental health. It is just that they become sad unnecessarily, transfer aggression. If spouses having misunderstanding, the person will be angry unnecessarily)

I: okay so you have not gone for any training?
R: Yes

I: and then your colleagues have not even been trained?
R: They have not gone for any training on mental health

I: While in the clinic, (speak Igbo: Do you have any experience on managing any mental health issues)
R: Noo

I: No experience?
R: (If we get anyone, the next thing is that we refer the patient to the doctor, then the doctor will refer the person to psychia)

I: Okay alright thank you. But now that you don't have any training eh, would you like to be part of any training on mental health challenges
R: yes yes

I: Okay
R: Because before I said I will do psychologist in university but unfortunately, I'm still doing my BSc

I: Okay
R: (If I see any opportunity for that I'll like to join)

I: Okay but why will you like... apart from being that you want to be a psychologist, is there another reason you will want to be part of the training

R: Yes. Another reason (I will like to be part of the training is that) I hate seeing people that are mental, so I like to be discussing with them, calming them down (Try to say things that will calm them down). Because a mental person like this, it is not easy, somebody goes mental in a family because totally you are losing the person (if I see such a person I like to talk to them, calm them down). That why I love... especially in a HIV clinic like this. I like talking with them, I like walking with them, I like to give them assurance and then any other thing that can make them better. To have a better life and a better....

I: Okay ma, thank you. I want to ask how common, how common do you think mental issues are, among our people that are living with HIV. How common do you think...You know you have been working with us in this facility. Based on your own interaction with people, you know our people taking this drug. How common do you think mental health issues dey in this facility? At least in this facility?
R: (inaudible speech)

I: As in how frequently, as in like how frequently can you say that you see people that have mental issues?
R: Mental health

I: Yes, in this facility. How frequently?
R: (I don't see patients with mental health but it's high. It is difficult to see them, we rarely see them)

I: So even when you are interacting with your patients, do you... have you noticed that there are some of them that needs like mental health care
R: Well, when you are interacting with the patient, (It depends on the patient. If it's an aggressive patient), what you will do is that you will calm the person to understand that this world is nothing and (he/she should calm down). You will start counselling the person for the person to understand that (he/she has to move forward if not the person can injure herself or himself). What we normally do is that, we engage the person in counselling

I: Okay have you really, now what I want to know... apart from being engaging, you yourself, have you ever interacted with someone that has a mental health issue
R: No, but when I was in antenatal clinic. So puerperal psychosis, after delivery, the person became... a mental this thing. I started interacting with the person, become a friend. I started counselling the person. It gets sometimes I will normally go to his house. I collected the number from the lady, so I go to his house to sometimes he normally come to... you know but he is not a HIV patient... he normally come to .... I'll him appointment he will come we will discuss (inaudible speech)

I: But our patient?
R: Our patient, I have never...

I: I have never seen anybody that maybe that is sad, that is depressed?

R: There is another person that is... I... I... counselled. The person is having issues with her marriage

I: Is she living with HIV?
R: yes, she is a HIV patient. She is having issue with her marriage after five children. She is only the one that is consoling the children., even the husband is negative. The husband started maltreating her. Every time the woman comes, she started being aggressive, she won't even come in time. When we asked her, she started being aggressive. One day, I just take out time to call the woman to ask her a question, what is the problem, you are always being aggressive, (what is wrong with you) calm down. In any situation in this earth, one day someone will close eyes and die and all those things they are doing, you won't remember it again, tell me what is your problem. (She said that her husband threw her out with her children) and things are so hard for her. You know I started counselling the woman but in good time the woman started calming down. Sometimes if I have even transportation money or any money, I'll give the lady. So, she started calming down.

I: Thank you. Apart from this person, have you ever had any encounter again with maybe somebody else that is going through a mental challenge due to one thing or the other and you did one thing for the person
R: Eh Ehn! There's a lady that normally come here. One lady, she registered last year with us, she is a very mental person. Anytime she is coming for appointment, she will come always around two, three when the workers are already dismissed. So (if I call her, we will go to normal store or [] and I asked her why she always coming, she said aunty I went here and there) but it's a mental.....sometimes when we have started giving her drugs, before the appointment the drugs will finish, she will come back for more and say she needs drugs that her drugs are finished. I'll say ah ahn, these drugs are once a month, once a day. How come you always come.... with lesser... when the appointment is not due, you will come back that your drugs are finished, what is the problem? (She said that it has finished). Now, I now tell her to give me the number of her mother, her mother's number. I now called the mother told her please this your daughter always come before the appointment what is the problem, (she said aunty please let me tell you the truth) she always takes the drugs two-two a day. I told her no... are you not the one giving the lady the drug? (she told me no) she always refuse her not to give her drugs. I said okay, I just cut the phone. I said to the lady that this drugs you are taking two-two, it'll affect your kidney or your liver. It's meant for taking once a day at the same time. You don't have to take it twice a day, that if you continue to take it like that, your kidney will fail, your liver will fail and now you will be battling with kidney and liver no more HIV, do you want to die (she told me aunty no-no I don't want to die). She's a young lady. What you will do now is that whenever I give you appointment you will come that day and bring the remaining drugs. That the remaining drugs is supposed to.... if I give you three months, three drugs are supposed to remain inside drug and you will drink it. Don't come before your appointment date and don't shake the drug too. I said do you want to die? (she said no). After the counselling and interaction, she stopped taking the drugs, because whenever I.... immediately I mentioned kidney and liver she just stopped it

I: But does she have any mental health issue
R: Yes, it's mental... yes

I: what mental health issue does she have
R: uhm we now ask the mother. The mother didn't give us the actual... what happens to the girl (she said she was in school when it happened even though she talks sensibly oo, but if you and her interact you will know she's not okay, even with the makeup she does)

I: Okay she dresses abnormally?
R: She dress well but the makeup is off point

I: Off point. Okay. Thank you, ma. Thank you, ma. I just want to know ma eh in your own opinion, what factors do you think contributes (background noise). I just want to know ma eh in your own opinion, what are the things that you feel contribute to mental health issues among persons living with HIV in this clinic? Did you get my question?
R: yes

I: As in what are the things, what are the factors, what are the issues, what are things that can cause mental health issues, that you know can predispose us. I don't know whether you are getting what I'm saying?
R: Well, I don't think that HIV contributes to mental health issues. Maybe in mental health issues I think it's from the hereditary, or from the smoke Indian hemp, or from taking mkpuru mmiri or all those things but I don't think that HIV can cause mental health but apart from the (inaudible sound) that if the patients can't start, they will be talking somehow but I have never seen that HIV causes mental health

I: okay, what we are looking at eh is not what are the causes of mental health. I'm looking at... you know in our situation, now in our environment, there are a lot of things that are factors, there are things that can.... problems and situations that we face that contributes to our mental health you know. I'm looking at... I'm asking in terms of our patients that are accessing care and you being a healthcare worker now, what.... in your opinion what are the things based on maybe
R: that can happen, that can cause

I: Yes, that can exactly
Together: contribute to their mental health issue

I: Yes
R: Well, one, what can cause is that uhm let me say, let me say, it can be maybe... it can be.... let me say (background noise)

I: You can pause it
R: (continues) What can lead that thing to the mental issue (it can be family issue or attitude to the nurses and other health workers that can cause) It may be.... yes, the main thing is attitude to the health workers. (Maybe that person has problems in the family, or the person doesn't have transport money. For example, a man that we scolded recently about missing his appointment, he told us that he didn't have transportation money and we were shouting at him, the man got angry. If he faces the situation at home and comes to face health workers). So, it's money issues and attitude of health workers that can lead a man to that

I: Thank you (Thank you). Are you aware of any program or activities that are available for screening, that's for preventing and managing mental health challenges among persons living with HIV in this facility?
R: I don't know

I: okay there's no....
R: Eh yes, I don't know

I: Of any mental....
R: Any mental prevention, yes, activities

I: Okay. Then, (silence) Then have you ever, what of you, have you ever used any mental health tool to screen for mental health issue?
R: No

I: Okay. Then if there are no mental health service, in your own opinion eh what are the things you feel should be available for people living with HIV to access mental health services in your facility? What are the thing you think we should put in place? I don't know whether you get what I'm trying to say?
R: I know

I: What are the things can we put in place? In this place, that you feel that if we put it in place at least mental health improve or mental health of the people will improve
R: If you bring... If there is (what's it called now) money.... this issue of financial support. If we can support them with the financial support and the... the major thing is financial

I: Is there any program you feel we should bring here or training or anything that you feel we can do here. I'm just asking?
R: Yea if you bring training it helps a lot for the patient and for the health workers

I: Is there any other thing apart from these two we mentioned? The financial support and training? Is there another thing that's in your mind that you feel (silence) that can improve the mental health of people living with HIV?
R: Training, (I have said) training

I: Yes, you've said training
R: Training and financial support

I: Okay, alright. Thank you, thank you for your time. Thank you so much. Do you have any question for us or comment or anything?
R: Hmmm (I like this interview you conducted) I so much love it because all these issues that we are having in HIV clinic. (It's much to the patients) so if all these you people are doing, if you people can come and support us and put training, it will be... the patients will benefit it more

even the health workers will benefit it. So that we will be able to reduce any mental or any challenges that the patient is having in our clinic. So, it’s very nice

I: Thank you very much. Thank you
I: God bless you

Transcript of key informant interview with healthcare worker 10, Facility Y

I: Let me now start with the question. Anyone you are comfortable with English, Igbo pidgin. So, the first question is do you know of any training or experience; sorry do you have any training or experience with mental health challenges among person living with HIV? Do you have any training? Have you undergone any training on mental health among people living with HIV in the facility?
R: NO

I: What of your colleague, do you know anybody that have gone through any training?
R: On mental health?

I: On mental health
R: No

I: Or experiences like maybe through this work, have you any experiences on mental health training or something?
R: No mental health training but maybe from this incidence now, you can see how we handle it, it's still ongoing anyway

I: So, but no training?
R: No formal training

I: Okay
R: Except the one we had when we were in school

I: Okay, but has anybody gone through training newly like given you people a stepdown training?
R: No

I: Okay, so there's no training
R: No

I: So, but if there's a training would you like to be part of it?
R: Sure yes

I: Okay why would you like to be part of it?
R: Anytime the training will start

I: Reason now, why will you be part of it?
R: Okay why?

I: Yes ma, why will you want to be part of the training?

R: It will help me to actually be involved in the patient's psychological care and mental support knowing that they are challenged in their health

I: Okay ma, Okay ma. How common do you think mental health challenges are among persons living with HIV? How common, how frequent do you think?
R: As soon as the person is diagnosed HIV positive, you see mental health, or their mental health will be challenged. Once they are identified as positive, on their own you will see the person manifesting with mental challenges like how am I the one that tested positive, why me, I think that's the right word, why me, why must it be me, so I will no more be get married again, I cannot give birth to children. That's usually the challenge they used to have once they are diagnosed positive.

I: So, is it common or it's not common?
R: Very very common amongst them

I: Okay thank you ma. Have you interacted like as a healthcare worker in this facility, have you had that any interaction with a patient with mental health issues?
R: Sure, some of our patients that usually present with eh eh encephalopathy you see them talking irrationally. Sometimes like the incidence was just observed, out of frustration, maybe economical challenge, in regards to the Nigerian economic situation now, some of them might not even have money to transport themselves to the clinic. You will see the person trying to show aggression, any slight question, instead of the person to comprehend what you asked the person, the person will want to react negatively. Until you try to explain further eh in the course of trying to explore the client, you will now see the person trying to understand what you are saying but ordinarily they usually present with aggression

I: Apart from aggression is there another symptom that you notice or patient usually present or complain about?
R: Some of them, you see them coming with eh, presenting with palpitation, you see them being unnecessarily tensed up. Some of them, when they have appointment, if you see them come to the clinic you will see the blood pressure going high, when you ask the person, the person will now tell you that if I remember that I have appointment that very day, you will see the person not being comfortable any longer but some of them will tell you that once they leave this clinic you will see them calming down, their BP will now come normal but if they remember that they are coming to the hospital their BP will start rising. You will see the person physically presenting with palpitation.

I: **Okay thank you ma. Then in all this presentation, how were you able to address them? Like you said about depression, you said about being tensed unnecessarily... what... as a healthcare worker, how were you able to ... what and what did you do to address these complaints?**
R: Ehm because I [was in] HCS [section], before now I enter into PMTCT. First of all, any client that test positive, you know in HIV here we usually do counselling on them. That counselling doesn't end on that day the patients tested positive, it's still ongoing. So, at every clinic visit, we try to enforce or emphasize the counselling as well, try to rephrase it, encourage them on the aspect of whatever challenge the person is presenting or complaint the person came with that

day. We still try to counsel, if it is in a way the person needs encouragement we also encourage as well.

I: Okay thank you ma. So, more of, you do more of counselling?
R: Counselling, mental support.

I: Alright ma, thank you ma. So, I want to know, in your opinion, what are the things you feel contribute to mental health problems among people living with HIV? I don't know whether you got what I said. The issue, what are the factors, things that you know that, these are things that contribute or causes this mental health issues among our client in this facility?
R: Well in that regard, some of them stigmatize themselves, they have self-stigma first of all. Then you will now talk about their social economic conditions' status. So, when you talk about that self-stigma, sometimes just like what I mentioned initially, they'll tell you that they.... they.... they tested positive which means there is no more hope in their life, everything has to end because they were diagnosed HIV positive but as counselling is ongoing, they will realize that so people can actually test positive and get married, bear children that are negative. So, at every clinic visit, we may try to enforce the counselling as well, then encourage them using other client that came to the clinic, have you seen this person, like this guy that came today that came on a PMTC clinic day, I have told him oh that see all these women that are coming here with children, they are also living HIV and they are having children. We also discharge some of their children that are negative which means... He was asking me which means he can get married and have children that are negative, I told him yes if you are actually adhering to what you are told to do. If your adherence is 100% you can live your normal live like any other person and be what God wants you to be and achieve what you want to achieve

I: Okay ma, so apart from this stigmatization and social economic status, is there anything that, other factors or other things that you feel that are things that can cause this mental health issues among our clients in this facility?
R: I think for now that's the two main causes

I: Okay ma, thank you ma. I want to ask, are you aware of any program or activities available for preventing and managing of mental health issues or challenges among our clients in this facility
R: I don't know but all I know is that if we identify such patients, we usually refer the person to go and see the doctor, our clinic, the clinician here. So, doctor will now assess and refer the person to see a psychiatrist. You know [name of facility] have psychiatric unit

I: Okay?
R: The one at []. It's either the....

I: At []?
R: Yes

I: There's none... they don't .... they don't have one here, any psychiatrist in this facility
R: They have but they usually consult at medical out patient in [] now

I: Okay Okay
R: But if the person after review at [], if the person needs admission, they will now refer the person to []

I: Okay... okay but in this ehm health facility, there is no mental health services, as in, in this our HIV clinic
R: Here?

I: Yes?
R: No

I: Okay
R: We use referral ... referral system

I: Okay
R: Cos this a teaching hospital

I: Okay. So, uhhm, so have you ever referred someone or have you ever used this to kind of manage? Have you ever referred someone for this errm [] hospital or...
R: Emene

I: Emene yes
R: Yes, from what I said usually if you identify such patients or if the person is talking irrationally like last... that was last month, one of our clients that came, the woman started behaving abnormal so the patient was asked to go and see the doctor. So, after seeing clinic doctor, doctor now referred her to see a psychiatrist. They went to that [], that our branch at []. I heard yesterday, someone was telling me that the patient was admitted at []

I: Okay. These patients, do they usually come back to you people after this...
R: Yes, when they are stable, they will now come back for their normal appointment visit

I: For their normal...
R: Yes

I: But do you people really, most times do you people know what they were treated of and how they were treated?
R: When the folder comes back?

I: Yes
R: Of course.

I: okay for that psychiatric issue
R: yes, they usually come

I: So, I want to ask how helpful has this program being in preventing mental health problem among people living with HIV. This referral, has it helped, has it been helpful, how helpful has it been?
R: Cos some of them that was identified when they were in acute stage, cos I know those ones that usually come, some of their nurses used to come here to collect their drugs from here, for their patients. But those patients...

I: Sorry, the nurses will collect the drug
R: They will come here and collect drugs for their patients

I: The psychiatrist nurses?
R: Yes, yes. They usually come here to collect drugs for them cos they cannot be coming on their own to this place. They usually come here and collect their drugs for them and we don't usually wait for those biometrics. Once they come, we usually give them like a month or 5 weeks drugs that they can take to go and give them there.

I: So, this will lead me, this question you answered will lead me to the next question now. I just want to know what are the factors that makes it easy for this our clients to access... what are the factors that prevent them from accessing this mental health services that you just talked about? I don't know whether you understood what I just said
R: Factors that?

I: Either they affect it. You know you just told me that the nurses come all the way, from all the way from [] to collect these drugs for these patients. So, what I want to know, what are the challenges that they now face in accessing this mental health service and also getting their drugs here. I don't know whether you understand what I'm trying to say?
R: For me the challenge...

I: Okay you understood it
R: Yes

I: Okay
R: For me the challenge that I observed is challenge of transportation fare. Then, they usually come with their hospital vehicle, they usually bring them but for some time now we've not seen them coming to pick drugs for their patient. I don't know whether it's because of this increment in fuel price. I have not seen them come for some time now but usually, they usually come, their driver used to bring them here. They will collect their drugs. Sometime they will come and collect for three, four, five patients that they have there. But you know there they also have side lab there at that [], so anybody that test positive, they will come here for enrollment. Once they enroll, they can now collect their drugs and be sending to them but since this hike in fuel pump price I have not send them come for a very long time.

I: Okay thank you. Apart from transportation, is there any other barriers or factors that you feel that affect this services, mental health services mostly from our patients accessing

their services apart from transportation. Is there any other factors or problems you feel affect their accessing their services there?
R: Anyway, the hike in transportation. I think that's the only challenge

I: Alright thank you so much, thank you but I don't know how to ask you this question (laughs) but let me just ask. This thing you just raised now, the factors, things of fuel, transportation. Do you think there's any way we can address this issue that's why I said.... (laughs)
R: (laughs) The challenge, because this is a government established hospital, let me call it erm government facility, I don't know how they are going to handle it cos if it would have been a private or faith-based facility, I will say since it is a private establishment they can fund it but I know protocol, following normal due process might take time. By the time they will wait for the protocol, they will follow the due process, patient might be missing appointment, they will be missing their drugs thereby leading to all this resistance

I: Alright ma, thank you so much for your time, thank you so much and then before we will stop, I want to know, do you have any question, or any comment or anything you want to ask us?
R: The question I want to ask, let me... anyway first of all I want to thank you for your time. You have been here since morning, waiting patiently to have direct chat with us. It is not easy, knowing the cost of transport now in the country and all the challenges everybody is facing and even our patients too but the question I have or my concern is here actually we really need training on mental and psychological management of patients. I don't know if there will be any training so far so that we can get more knowledge on how to handle all these patients that have mental challenges. If there will be any?

I: Yes, thank you. That's what we are trying to do you know. That's the essence of trying to....

I2: (overlapping) That's the essence of this research

I: Essence of this interview cos we want to know...

I2: (overlapping) Have evidence

I: Yes, on what are really the issues so that when we are doing the implementation of this mental health or training, we will now know what and what we are looking out for and the training, who and who will be trained and what they should be trained on

R: Thank you very much

I: Thank you

Transcript of key informant interview with healthcare worker 11, Facility Y

I: Okay so, good morning, Ma
R: Good morning

I: My-my name is []and I am here on behalf of the epidemiology research unit of [name of facility]. We are here to understand from your insights about mental health needs and factors that have eh, influenced access to mental care by women care givers living with HIV, who come to your facility to access care. Eh, so this interview will last for maybe about 30, 35, 40 minutes, or even less. Uhm your participation is voluntary, and all information will remain confidential. I will be using a tape recorder just to ensure information is captured accurately. Uhm, at this-at any time during the conversation feel free to ask any questions, or to inform me if there is any question you wouldn't want to give specific answer to. Okay? So, at this time, do you have any questions for us?
R: Hmmm, no, not really

I: Okay, okay
R: At least not for now, let's just be going

I: Okay, okay, alright, thank you very much. So, may we begin the interview now?
R: Okay

I: Alright. So, Ma, my first question uhm, is do you or any other colleague of yours have any training, whether its professional training, or on-the-job training or any experiences with diagnosing mental health challenges among persons living with HIV that are accessing care at your health facility?
R: Well, uhm, I cannot tell if they have gone for any training like that, but I can speak for myself

I: Good
R: I have not gone any training of such, that eh, mental health challenge, no, I haven't, so I can't speak for others, maybe they have. Eh, so when I was at the community there, we had so many trainings but not mental health challenge in particular

I: Okay
R: So, I can't tell

I: Okay, alright. Thank you very much Ma. So eh, my next question will be, will you be willing or will you like to be part of any mental health training in the future that will equip you with maybe the skills to be able to diagnose and manage mental health challenges among this group of clients?
R: Yes, yes
I: Okay, why would you like to be part of that kind of training?
R: Yes, because eh I am meeting-I meet-I'm meeting them every day, from Monday to Friday, I do meet them, so, many-some with their challenges, others with their own challenges, so-so that

if I'm being trained in future, I can-I will know how to manage any—ehen any situation or any challenge that comes around, yes.

I: Okay, alright, thank you very much Ma. Uhm, I will like to know, how common do you think mental health challenges are, among persons living with HIV?
R: Uhm, well, just as I said before, people with their challenges, with their problems anyways, like 1, I met 1 the other day, so I was talking with her, so I was asking her questions, I noticed that she was not even there, I was busy talking with her, asking her questions, she wasn't answering. So, I called her Ma, I'm talking to you, are you there, she said sorry, sorry, so many things on my mind. So, then I noticed that, that woman she has been going a lot of eh-going through a lot of problems, ehen, so, I don't know

I: Yeah, so-so-so yeah, so that's-that's very good, no very good Ma, uhm your responses are in order. So, I-I- you've just told us about one particular case that you encountered
R: Yes

I: Are there other such cases that you have encountered in the course of your work in this facility, or even before, eh, working in this health facility?
R: Yes, yes, I have, many of them. Okay, let me not use this word many, some of them, I have encountered. Like eh, sometime ago, I was eh asking one woman eh, okay I wanted to enroll the woman on OVC program. So, she was like please you people should not disturb me, stop disturbing me in this uhm, blah blah blah blah blah, you people are not going to help. I was like Ma please can you calm down, calm down please, hear me out, let me finish. Then if you are not interested you can still tell me that you're not interested. So, I noticed that, like she does not even want anything, since you mentioned HIV, since HIV is involved, she doesn't want anything, she doesn't want anybody to come around her, she doesn't want nobody, even her family friends, I noticed that she does not associate because of this eh HIV. So the other one, the other one said eh that because of this HIV, if I got the market to sell, I won't be able to sell it, that people, that she doesn't know whether people knows that she has eh HIV or not, but that she does not want anything that she-sorry, that when she goes to market she won't be able to sell her vegetables that-that the reason is because that she's taking these drugs, she's HIV positive, ehen, that's the reason people doesn't eh patronize her market, ehn so, so many eh-eh-eh-eh problems, different problems, so, I don't know.

I: Okay, no problem Ma, thank you very much Ma for that response. I would like to know, for some of the cases that you have seen and you know believe that they may have one mental health challenge or the other, can you tell us you know, how you tried to address that mental health challenge that you observed in that-in those patients, or in 1 or 2 of those patients?
R: Okay, ehh, 1, eh I enrolled them in uhm support group

I: Okay
R: Ehen, so when they-when they attend meetings at least they will-they will hear from other positive-positive patients, what they have been passing through, how they managed to calm the challenges they're having, how they managed to solve their problems, ehen, so you enroll them

to support groups like eh, we have one here, then we have at eh community, support group then, ehen then we enroll them on savings group-savings, we call it eh VLSA savings group, that is eh Village Savings and Loan Association ehen. We link them there, they'll be coming out every week with their colleagues-with their colleagues, when they-I mean when they come out, they will learn from others how they manage to solve their own problems, so we link them to so many, eh support groups, yes.

I: Okay, alright, thank you very much Ma. So, in your-in your own opinion you know, uhm what are those things or factors that you think are contributing to mental health challenges amongst persons living with HIV that are coming to your facility to access care?
R: I didn't get it

I: Okay. Like what do you think is causing these mental health challenges uhm for people living with HIV, what are the things happening either in the society or in their personal lives that you think is contributing to or you know making them come down with mental health issues or challenges?
R: Stigma, stigma one. Discrimination two. Poverty three.

I: Okay thank you very much Ma. So, you have mentioned stigma, discrimination, poverty. Are there any other ones that you can think of that comes to your mind, from your experience so far?
R: No, these three things. One, I wanted to enroll one on OVC program, so I was like eh, telling her about OVC, I noticed that she's really-she's interested. So, when I was discussing with-I was discussing with another person anyways so, she was there listening. So, when I finally went to her, I was telling her about the OVC, she said, she asked me if I'm the one that will be visiting her, I told her No, that I will give you somebody that will handle your household. She said No o, ah please o, if you're not the one coming, don't bother sending anybody to my house. I said No, just wait, the person I will give you is a professional, someone who is trained on that particular job. She said No. Then I now asked her why. She said what if I know the person, what if the person knows me. So, I told her even if the person knows you, the person is not going to tell anybody that you're taking these drugs, if you hear it anywhere, just-just know, have it in mind that you-you are the person that told them that you're taking this drug. So, she said no, no, no, no, okay, okay, can the person come to my shop, I said yes. Just tell the person where you want him or her to be meeting you, that the person will gladly come there. She said what if the person comes to my shop, and she saw another person that she knows, that she might-that the person might give eh, okay at first, she said she doesn't want a man to come to her shop, that her husband will not understand. I said okay, no problems, I'm not going to send a man to you, I'll send a woman. She said okay, what if the person came to my shop and saw another person that she knows, she can equally just give the person sign. I was like okay, if the person gives sign, okay the sign means that you're taking drugs? She said no, you'll not understand-you'll not understand. So, this kind of a person, haa, I don't know.

I: So, you mean like the fear of stigma is the major contributing factor for some of these people?
R: Yes, yes, yes

I: Alright, thank you very much Ma for your responses. So, my next question is, uhm, are you aware of any programs, of resources, facilities that are available for preventing or screening or managing mental health challenges amongst, uhm, HIV positive people coming to receive care at your facility? I know you've mentioned support groups, eh you've mentioned uhm-you've mentioned the-the voluntary savings
R: Savings

I: Village savings and loan association, so in addition to these two, are there any other programs that you're aware of that is available to help in alleviating or minimizing mental health challenges for this group of clients?
R: No, I, I've-I've, no.

I: Okay, okay, Ma. So-so, maybe let's be a bit more specific, uhm, so, in addition to these two eh, uhm what other programs or resources do you think, can be made available to help with preventing or managing mental health challenges for this group of clients?
R: Okay, like you can-you can as well enroll them on skills acquisition, those that doesn't have anything doing. You can enroll them there, ehen, so when they are going our everyday, I believe their problems will be solved, if not solved entirely, but at least to an extent.

I: Alright Ma. So, uhm, let's come down to the available ones you mentioned, the support groups, the village savings and loans association. Eh, are these programs, are they tailored specifically to also help women caregivers living with HIV. That's women who are positive and also have children that they're taking care of. Are some of these programs designed in such a way that it is beneficial specifically to this group of clients?
R: Yes. Yes now

I: Can you, you know, like uhm explain a little bit more for us?
R: Please can you-can you take that question again?

I: Okay, so I'm asking, you know earlier on, you mentioned that there are-the programs you're aware of include: support groups and also village savings and loans associations.
R: Hmmm

I: So, but these programs are available to almost everybody
R: Yes

I: So, I wanted to know, are there measures put in place for-in these programs that cater for specifically women, and not just even women, women who are caregivers, and what I mean by caregivers is, women who are HIV positive and also providing care, maybe they have children under their care that they are also providing care to?
R: Yes. When they join eh-when they VLSA, from there they will be meeting other women, like the one we have here, I know that VLSA, has been going on here, that eh support group, I don't-I

don't know, on that Saturday, if you notice eh, they-they brought a box, metal box, I don't know if you saw something like that. Okay, that's the box that they using for that VLSA, for that their savings, they contribute, ehen they have a particular amount that they contribute, at the end of the cycle, they will share it, ehen. So, I believe that when you're saving-when you're saving, when you come out, you see other women, even if you believe that your case that your case is worst, if you join them, you will see those people that you're better-you're better than. Eh, so you'll be saving, no matter how little it's something, at the end of the cycle when they will share it, you carry your own savings. Ehen, then it will help you, even your school eh, your children's school fees, it will help you, their clothing, food, so many of them, so many of them that you can use your savings to achieve. Then, the other way round, the other eh, support group, when you're coming out, the thing is they say that idle mind is the devil's workshop. I believe that if you're coming out, you'll be learning so many things, you-you will see people that you're better off, but they're happy, even if they did not eat, they are happy, even if they're sick, they are happy, because, for me, HIV is not sickness o, for me, it's not sickness. Just that the person has virus, it's not sickness. But when you come there, you'll see people with sickness but they are happy, because they are meeting eh, they are meeting people that-that people that can teach them, people that can talk to them, make them to ease their-their pains. Yes, so I believe that, that social eh, support group helps a lot.

I: Alright, thank you very much Ma. So, uhm my next question is, uhm have you used any of these services or programs that are available to prevent or even manage mental health disorders, for patients that you have seen eh, in your health facility?
R: Here?

I: Yes
R: Yes. Yes. At least I have 3, 3 of them. We, from our, from community, from CBO, they gave them, anyways Caritas gave them IGA, Income Generating Activities, supported them with Income Generating Activities, 3 of them, yes.

I: Okay, alright, Ma. So, what kind of mental health problems or challenges did you think that, you know, linking them up with to these services helped you to prevent or manage for them. Uhm, what mental health problems did you-were you able to prevent tor manage for these 3 patients, just by the fact that you were able to link them up to some of these support groups that you mentioned?
R: Okay, okay. one, I met one, I met her husband at [name of facility]. So, he took me to their house, when I got there, I saw another thing. The house that they're leaving, in fact they're almost, in fact they're almost living outside without shelter. So, I met our wife, so I-you know, women to women, so I discussed with her, she has nothing doing, the husband has nothing doing, the children are not going to school, so, so many things, so many stress on them. And eh, she has a baby there, so I asked her, Ma how are you people managing? She said that they are begging, whoever that cares to support, supports them. I said okay, so I took her name to Caritas, to my CBO then, eh so that's how they supported her with IGA.

I: Okay, okay, alright, thank you very much Ma. Uhm, my next question is how helpful do you think these programs that you've mentioned are to these women?

R: Hmmm, more than o, more than. This one-this one that I just mentioned, if you go to her-if you go to her house now, at least her husband has something doing now, she—she has some-she has shop, their children is now in school, so it-it helps them very very well

I: Okay, thank you very much. So, we are almost at the end of our interview session, but I just want to also know, uhm, what are the factors that you think, make it easy for these patients, to come and make use of these programs that you've talked about?
R: What are?

I: What makes it easy, you know, you mentioned that, a lot-for-for- like you gave an example where you were trying to enroll one into a support group but she was so scared about stigmatizing and discrimination from people knowing about her status. So, that's like stigma is making her not, you know, not to want to be part of that program, or benefit from that program?
R: But I later enrolled her anyway

I: Fine, so what I now want to know is, what makes it easy, what is it that makes it easy for women to agree or to participate in these support groups or any similar, eh services that helps them with their mental health challenges?
R: Well, uh for me, I cannot just tell, I will just-my own is that I will talk to you, I will tell you what and what you will benefit, though I will not promise you that we are going to give you money, no, we don't do that, eh but I will tell you, I will convince you, I will make you to see reasons why, you want to join the support group, ehen, so if the person agrees, I will enroll.

I: Okay, alright Ma.
R: But the person has every right to decline.

I: Okay, alright, alright Ma. So, but are there-are there other challenges or barriers, you know that limits women caregivers from accessing mental health programs in your facility. You've mentioned stigma, as an important barrier or challenge. Are there others?
R: Hmm, some will tell you that we are coming from a very far place, that we won't be, that they won't be making it because of eh transportation, at least you know what is going in Nigeria, so.

I: Okay, any other one apart from you know, high cost of transportation?
R: Maybe, sickness

I: Okay, alright, thank you very much Ma. So, how can these barriers be you know, addressed or minimized? You know you've talked about problems with stigma, cost of transportation, ill health.
R: Ill health, yeah

I: What ideas do you think can be explored to minimize these barriers so women caregivers who are living with HIV can surmount them and come and utilize any programs that are available to them, to help with their mental health challenges?

R: Okay, like eh, maybe setting up eh this eh, support group in their localities

I: Taking it closer to them
R: Closer to them, yes

I: Okay, any-any other, any other ideas?
R: Maybe if you can help them financially, but you know, if you help this month, you're going to help next month, because the support group meeting is every month, though it's once a month, ehen, so, just support in any way you can financially, if the person really wants to join, I believe he or she-

I: Will join
R: Yes

I: Okay, alright Ma. So, thank you very much Ma'am. At this point we have asked you all the questions we have for you, but I want to know, is there any question that you will like to ask me at this point?
R: Yes, I want to ask

I: Okay Ma, go ahead.
R: So, with all these things that you've answered, that you've asked and I've given you eh, what I feel is their problem, so is there any way that you people are going to help?

I: Yes, so that is why we are asking these questions from professionals like you. We want to understand what the challenges are, with regards to accessing mental health solu-you know, accessing mental health services for this category of clients, so that we can now know how to come in, or where to make recommendations for people who want to come and support the mental health programs that are already in existence or even start up new mental health programs or provide mental health services to this category of clients. So, that's why we want to get your own professional inputs from your experience working in this sector to know how best those programs can be designed so that the women caregivers who are HIV positive can benefit maximally from those programs.
R: So how do you intend doing that, because at least these people that we are talking about, you don't know one on one, so how do you intend doing that, how do you intend getting them?

I: So-so-so, we'll also go through the health facilities and professionals like you, so this is still the first stage of what we plan to do, which is to, get ideas from your experiences working with these people, to understand the problems and possible solutions to them, then by the time, we articulate all the responses we are getting all the professionals that-healthcare workers that we're asking eh, interviewing, we can then pick out the solutions that we think are practical and feasible, and then, we'll also come back to you to say, these and these and these, are the solutions that we have and we think we can do, we now need your assistance in helping us make sure that it gets to these people, whether it will be at the facility level or at the community level.
R: Okay

I: Alright, so thank you very much Ma, for your time, and for participating in this interview, we appreciate.

Transcript of key informant interview with healthcare worker 12, Facility Y

I: Alright, so good morning, ma.
R: Good morning

I: My name is [], I am here on behalf of the epidemiology research unit of [name of facility], Enugu. And we are conducting this research to gain insights into the mental health needs and factors that affect access to mental health care by women givers living with HIV in Enugu state, Nigeria. Ehm, your participation in this interview is voluntary, and all information will remain confidential, this interview will last for maybe about 20 minutes, 25 minutes maximum. Eh, I will be using a tape recorder just to ensure that all information is captured accurately. Eh, at any time during the conversation, please feel free to ask me any questions, or to inform me if you would rather not answer a specific question, eh at this time, do you want to ask any questions about the interview?
R: You have already explained yourself, that you are a doctor and you are from [name of facility].

I: Yes, thank you very much Ma. So, may we begin the interview now?
R: Hmm, let's begin, anyhow, any-anyhow, any one I know I will say now

I: No problem Ma. Thank you very much Ma. So, our first question is ehm do you or any other colleague of yours have any training whether it's professional training or on the job training or any experiences at all, with diagnosing mental health challenges among persons living HIV that come here to access treatment in your facility?
R: No, but as a nurse, eh you come across mental health challenges

I: Alright
R: But we have not trained based on that specifically

I: Okay, alright Ma. Thank you very much Ma. So, I would like to know, would you be willing or would you like to be part of any such training, any future training, eh, on mental health for diagnosing and managing mental health challenges among this group of clients?
R: Yes, it's very-very important, because when you come across such people even as a nurse, you'll be able to know how to manage that person and do that person so that the person will live a normal live, even with HIV

I: Okay, okay Ma, thank you very much Ma. So, I want to know from your professional experience, how common do you think mental challenges are, among persons living with HIV?
R: Hmmm, yeah, it's very often, very common because especially newly diagnosed HIV patients, first of all when the thing is announced to them, that they are positive, their reaction, they will start to think. Is this not HIV that usually kill, unless after the counselling, that you will now tell the person it's not the way that they know HIV before, that's is it still existing now, that

they can live their normal lives with HIV drugs. Then after counselling the person, some that are educated will understand it, some, it will still-they will feel depressed, and if you don't counsel the person and even call the person on phone to know how the person is feeling, with that depression, the person might not live long, because she will feel eh, that eh stigma, there will be a set of stigma, yeah. Will have stigma and discrimination, seeing-cannot associate with people maybe because hei I'm now different, if I go now, these people will now-they will-they will find out that I'm posi-I'm HIV positive, and there will be a sort of discrimination. So, she will be withdrawn from organization.

I: Okay, alright, thank you very much Ma. So, I would like to know, have you in the course of your work, interacted with a patient or patients with mental health challenges, and what we mean by mental health challenges, you know you already mentioned that some of them may feel depressed especially when you-when their status is disclosed to them after they've-after they are just newly tested. So, we are asking about those kind of scenarios, maybe anxiety-
R: Whether I have-I have come across

I: Whether you've had any such encounters
R: Yeah, yeah. I have come across such, and the person did not accept it, so, after, I-I followed the person up, so he was telling how he has even, that the master wants to help him to travel abroad, and he just came because he's having diarrhea that doesn't stop. He just decided to come and check, have a general checkup, he never know that he will-that this will come up, how will-how is he going to cope. Is it when somebody-when God wants to bless somebody that the person will start having problem? I started counselling this man, after series of counselling-after series of counselling, eh he went and told me that he doesn't have money even for, you know, there are some like some tests that people, somebody will do before commencing the drug like ALT and Creatinine with PCV, that he don't have money for such and even obtaining the folder, so I now I encouraged him that-, he did not come until when the sickness started, he started-the thing persisted, so when the master now came and saw he's down and gave him money for that. So, he did not accept it, because he said I cannot tell my people about this, if she-if he inform the mother, the mother will die before him. That it's rather-it's better for him to die than exposing this, he cannot disclose it to anybody. So, but after much encouragement, he was happy with me and now he's coping cause he's coming to take drug and he's better now.
I: Okay, alright, thank you very much Ma. So, Ma, I would like to know, you know, how did you try to address this patient's problems around denial, fear of stigma that you mentioned to us? How did you eh try to address this concern of his that could have potentially led to serious mental health challenges for that patient?
R: For that particular patient?

I: Yes
R: Ah ah, I told him that this is not the end of the world, that HIV does not kill again, unless somebody, if-if anybody that die of HIV now, it's the person that cause it because everything is being provided, and they make sure that the drugs are free, and this HIV is even more better than somebody that is suffering of cancer, liver problem, heart failure, diabetes. I mentioned all these things to him, and their drugs are expensive and this one, everything is free. And you don't even eh-eh-eh, only-only-only things that contain eh caffeine that he doesn't eat, that you can eat any

other type of food and you live normal life without anybody knowing. So, that, I tried my best to make sure that he understood what I said.

I: Alright, so in-so in essence, your counselling and providing health education to that patient helped to address the person's mental health challenges at that point in time.
R: Yes, yes

I: Alright, thank you very much ma. So, ma, in your own opinion, working as a professional, and health care worker in this facility so far, what factors are contributing to mental health challenges among persons living with HIV that come here to access care?
R: What?

I: What are those things that you think are responsible or that lead to patients who come here to receive care to have mental health challenges, whether it's depression, whether it is anxiety, or fear of stigma?
R: Yeah, yes, yes. Anxiety is one of it, depression, stigma and discrimination, death, fear of death, eh and what again?

I: Alright, thank-thank you very much ma.
R: And even eh, lack of education and resources. Some, no-they would not have time-money, they don't even understand it, they are not educated on that and even resources, in terms of money for transportation. You know everything is free, but some will tell you that they don't have money for transportation. So, these are the challenges, cos there are people you-you will diagnose of this HIV, and you-you find out that they are very-very poor, they can't even feed very well, so I think.

I: Alright, thank you, thank you very much ma. So, ma, you know you mentioned lack of resources as one of the challenges or factors that may be leading to mental health challenges for this category of clients. So, I wanted to know, ehm are you aware of any programs or resources that are available in your health facility for helping to prevent, or screen, or manage mental health problems among persons who come here to receive care?
R: In our facility?

I: Yes, young HIV positive persons who come here to receive care
R: I didn't get this question

I: Okay, so are there any health programs or resources that the health facility provides or that is provided in this health facility to help in preventing or managing mental health problems for persons living with HIV?
R: Not really, unless eh if that person is admitted and we find out that the person have a problem, he doesn't have any resources, from that point in time, we inform the social-the social workers, the-is that social workers? So, they will come and take, eh they find out the person that have nobody to help, ehen, they will now inform the management, so if the person, all the treatment that the person is receiving, the management might write to take care of the patient and write everything inside the folder, after which they will ask the patient to go home without payment.

I: Okay Ma, okay Ma. So, but I wanted to know, are there mental health programs you know-you know, that are available to help, for example if a patient comes and there's a program in place that helps detect whether that person has any mental health problem. Maybe depression, maybe anxiety, maybe any of the other mental health problems. Are there any structured program available in the health facility that helps with detecting that? And then, are there programs that when it's detected that also helps with managing those kind of mental health problems?
R: I have not, I am not sure of that one.

I: Okay, alright, okay, but do you-do you think, what kind of programs or resources do you think can be made available to help with either preventing mental health problems or managing mental health problems for persons living with HIV that come to the health facility for treatment?
R: For the facility?

I: Yes, either providing it here in facility or you know, or even in the community. You know, whichever one you think is, best addresses the mental health problems of patients
R: Hmm, just like, no it's supposed to, maybe they map our some people in the clinic to detect-when they detect such people, they should inform the management that this is the challenges, we are having, and eh, so that management will now, I don't know how I will put it.

I: No problem, I-I-I think get what-where you're going to, you know if persons who come here to receive care for their HIV status, are found out to also mental health problems, there should be people in the health facility that should be able to detect that, and then relate to the next authority
R: Exactly

I: Whether it's management or any other department that can provide care for that mental health problem
R: Hmm, you know we have psychiatrics here but it's in []. Ehen, when it is like that, we can refer, you know, when we send the patient to the doctor, the doctor will now-after assessing the patient, and needs to refer to the psychiatrist, doctor will write a referral note to psychiatric hospital in-at [].

I: Okay, alright, okay, okay Ma. So, Ma I wanted to also know, ehm ehm, have you ever had the need in the course of your work to you know, try to detect or screen for mental health challenges among patients that come to-to your health facility?
R: Hmm, since I came in here, I'm new here, I've not gotten any

I: Okay

R: Yeah, I've not gotten any. The only thing I found out is IPV, this eh, eh intimate-

I: Partner Violence
R: Partner Violence, that I heard. The woman was so agitating what the husband was doing because she's positive and the husband is negative, ehen, so that was, and how we managed that one, and there was no money also, we managed that one by informing the Caritas, the-GBV, what do they call those people?

I: Gender Based Violence
R: No, eh we informed the Caritas who now said we should send to, refer them to another facility that does not require some payment, they don't require test and other thing before commencement of eh-

I: Treatment
R: Drugs, yeah, which we did, because the two children later came out positive also, ehen and that- the woman pleaded that we should not inform the husband that the two children are also positive, which we informed the facility that we referred them to, what the woman pleaded for, because of she is afraid of what he is going to do to her, so, that was only the one I have comes across since I came in here.

I: Okay Ma. Thank you very much Ma. We are almost at the end of our interview, but I want to know, ehm, are there anything that make it easy for women living with HIV to have access to mental health programs that are available, whether in this facility or even in a nearby facility?
R: Hmm, all I know, like here, I think they used to-we used to tell them, they used to have meeting once every year-every month, I think last-last Saturday of every month, where we educate them, so that-what they supposed to know about this HIV, because if anyone-someone is very much aware of this HIV, I don't think mental health problem will arise. So, we do educate them, based on such. Once every month, so, there is a program that is done but it's not everybody that used to come.

I: Okay Ma, alright. So, are there challenges, are there barriers that make it difficult for women to come for these kind of programs that you talked about, like the support groups that you just mentioned?
R: Hmm, some of them are saying, that's the resources as I said, that they don't have transportation fare for it, that is it. Some because like some-some women maybe they've not disclosed it to their parents or to the partner. So, they are afraid of telling them where they are going. Do you get?

I: Yeah
R: So, this is the two thing I can say is the challenge now

I: Alright Ma, so how can these challenges or barriers be addressed, how best can they be addressed so that it doesn't pose a problem for persons living with HIV from coming to access these mental health services?
R: Hmm, I believe the-the-they should-they should eh, maybe find a-those people that are having those challenges, you look at the area that they are living, you can map out outreach for that place, where we go and do some health talk, invite everybody including that person. Ehen so, and the person will now receive those-even the treatment and the health care he's supposed to hear which will help her. But you have already known the person, but you are doing it general, so that you will not eh-the people there will not know whom you are talking, but the person you are saying-telling that thing will know it. You have accessed the health care in her or hi-her own doorstep.

I: Okay, okay. Alright, thank you very much Ma. Eh, is there any questions that you would like to ask me at this point?
R: Hmm, the question I would like to ask is this, like there are some challenges we having here, not only women, but I know your research is based on women, some that are being diagnosed of this HIV and they will not, they will never come back even to lead to care. Reason, some if you call them they will say they don't have money because eh they cannot access the care without having folder, they cannot access the care because we need to check about their the-the condition of their kidney and liver by doing ALT and Creatinine. So, if there is any, a way, as they have made sure that their drugs are-are free, is there a way that they should liaise with management even to help these people, so that when this is being detect, maybe those tests will include being free of charge for them, so that whenever we get it, everything we just start

I: Alright Ma, thank you very much for this question, so it's part of why we are looking at this, trying to interact with health care workers, and to understand the challenges that these clients face, so that if we are designing any interventions to help them address their mental health challenges, it will be an all-encompassing intervention that can address whatever factors that may be contributing to their mental health problems, you know, you've mentioned the issues around socio-economic problems, about not having transport, not having money to access some basic things that are paid for before even getting the drugs that are free, you know, so all these are likely to contribute to their mental health problems, so that's why this our interview is important. And eh we thank you so much for your responses and we will look into these responses and then see how best we can design any interventions that we have for this group of clients.
R: It's okay

I: Yeah, thank you
R: Thank you

I: Thank you so very much Ma, I truly appreciate.

Transcript of FGD 1 with women caregivers living with HIV, Facility X

INTERVIEWER	RESPONDENT
**(Some doctors who work here are responsible for this gathering because we need mental health experts to come here. That's why I said Government. I didn't want to talk too much because we have started recording)**	(speaks first)<br>R9: (Who are those that organized this gathering?)
**The first question I want to ask you is that since you became aware of your HIV status, how did it affect your mental health well-being? From the very first time you knew; how did you feel?)**	R8: for me I get angrily easily, I get angry easily and I'm depressed because not everywhere I go to I'll take my drugs and it's giving me stress because of my work and everything just stress. I can just get pissed off, like this morning when you called, I was so angry because I have to say not less than... I'll say my going and coming will be about sixty thousand just to come is thirty thousand and I have to lodge. I lodge six thousand, me coming here, me coming here, transport and I'm still going back so I get pissed easily and if you don't understand... I don't know what to say. It's a welcome development for me. They should bring them
**Yea thank you (Thank you. Is there any other person? Number two wants to talk)**	R2: I am number two. Thank you for this organization. I am really happy. Because for me (this really affected me so much. When I got HIV, I felt like dying but to God be the glory I got myself back. I didn't get married to the person I was engaged to but I eventually got married and gave birth to children. My challenge is that I don't go to places I want to go, I can't eat what I want to eat like coconut, as long I'm taking my drugs I can't eat it. I can't meet up with appointment because of the nature of my job. The little salary I get, I spend it on test and transport. I am very disorganized) I really want them to come to help us

**Thank you. (Is there any other person? Number five)**	R5: (I didn't know when it started. It started as a stomach pain and running stomach. I drank all kinds of medicine. I went to park lane and I was referred here to UNTH where I was given medicine)
**(How did it affect your mental health?)**	R5: (I couldn't eat anything until the money and drugs I was given before I was able to eat)
**(Mummy please try to speak up. Let's ask number one. Let number one contribute)**	R1: (Please I thank the people that organized the meeting. Nobody is truly happy when they are ill. The condition of the country isn't helping either, my children and husband are treating me badly and giving me headache and life is not being easy, so we are pleading with the government to help us to reduce the stress. I greet you all)
**Number 4... Number 4**	R 4: (I greet everyone. The first I got the infection was when I gave birth recently. My husband first contracted it and didn't tell me. What you people should do is that, when you ask people to come and collect drugs, if they are married then their spouses have to be present when they're collecting their drugs. My husband has been collecting his drugs for years when I hadn't contracted it. I gave birth to my first child who is infected now. It was when I gave birth to my second child that I found out my status, luckily, he was just four months old. I took my first child to the hospital for test and found out, he was positive. I began to ask questions, that's when he brought me here. My second child didn't contract HIV. The reverend took care of my child well. Each time I remember the incidence, I get angry.
**(Thank you. Number nine)**	R9: (Number 9. I want to thank the people that organized this meeting. God bless you all, it's not easy. When I first found out about my status, it felt like my world was over, but I was able to pull myself together. Anytime I remember that I need to take my

	drugs or I need to travel, I get so uncomfortable. I think of how to handle the drug without anybody recognizing me. Even at my place of work, I don't tell anyone where I am going and people are dying in silence. If there is a way you people can do it.... most of us are not happy taking the drugs. So, I thank you all, if there's a way you people can help us, please do.
**(Thank you. Number seven)**	R7: Number seven. I greet everyone. When this thing started. I thought I was going to die but I thank God for today. Anytime I come to collect drugs, the doctors always encourage me but I want to plead with you, the price of the lab test, 5500, it is difficult for some of us. Please find a way to help us. I thank God for the doctors and nurses that attend to us, they give us helping hands.
**(There's no problem. Thank you. Is there anyone that want to talk. Pause this recording... I want to ask. There go to the next question. We are about rounding up, there isn't much to ask. I want to ask, when you people come here, do the doctors or nurses or counsellors that attend to you, ask about your mental health? I'm not talking about the other side of health, how many of them ask you)**	R1: (They have mentioned and they also ask us other about things)
**(Thank you. Number seven)**	R7: (They asked me today for the first time)
**Thank you, number nine... number eight**	R8: First time, first time
**Okay than you (Thank you. Let's move forward) Based on experience (what is experience? You people know what is experience) (laughs) (Based on your experience, what are the things, factors that affect our mental health)**	(inaudible speech)

**Eh ehn exactly yes. Number two**	R2: Number two (our experience... whenever we remember that we are taking our drugs, and we have HIV, we get angry. For example, when I gave birth recently, my family got to find out and they were not happy and I also delivered through CS. So, it caused problem between me and my family. Each time I remember it, I find it too difficult to eat, go out, even my neighbors found out and tagged me HIV people. Whenever I remember all these things, it makes me unhappy)
**Number one**	R1: (Another thing is that if your husband got it first and hid it from you and you found out later. Whenever you see your husband, you feel like hitting him but you calm down when you see people around, you begin to have headache)
**(Thank you. Number six)**	R6: (My experience is from some of your doctors and nurses). I could remember that when I so first of all discovered it in me. Instead of to hide it and guide me, she went and told her siblings. They now made it known to people. When I'm passing, they will say you have HIV, each time I see that nurse I get upset. That is all.
**(Thank you) Number eight**	(silence)
**(Pause it)**	R8: Each time I remembered my ex that gave me this thing, I just hate men for no reason. Like I don't have boyfriend and... I don't trust people because of that, I don't trust. I lost trust on people because of it. The stress alone, you know I'm still young, ah for... I'll say this thing now for the past twelve years I have been dealing with this and it's not easy for me. I'm still young. How long will I go? That's the thought I have been having.

**Thank you. (Thank you. Is there any other person. Is there anyone here experiencing family issues coupled with your status... Does family factors that contribute to your mental health?)**	All: Yes<br><br>R1: Number one
**Number one**	R1: If you want to run test in the lab, lab fee is five thousand five hundred naira. You will buy what your family will eat. Instead of running for test, you use the money to eat and be happy
**(Thank you. Is there any other person? Number nine do you want to talk?)**	R9: (when you remember ... just like when you come. They will tell you that it's time to run the test and you don't have money. You will become confused and disorganized.
**(Thank you. Number two)**	R2: (This is very important; it's concerning the lab issue. When you go to talk to people in the lab)<br>The way they will talk to you, you might... that is slumped immediately...<br>(madam please go if you don't have money now, you can come when you have the money). They will not even.... that's... pet you, you know this our condition needs petting. The way they will talk to you; you will say let you go and hang yourself immediately. Please you people should do something about it
**(Thank you. Is there other person that has anything to say about family issues contributing to this? Anyone?)**	No response
**(Thank you. Another question I will like to ask is that, have you heard of any program in this facility that addresses mental health issues amongst you. Are you aware of any activity or program or anything?)**	R2: Yes, Number two.

**Okay**	R2: (Yes, I remember, there has been a program about mental health by … nurse []).<br>So, she keep doing it even on WhatsApp, on Facebook. She's the best counsellor ever here
**Alright thank you (Any other person? Number... eight)**	R8: I have not heard... It's only nurse [] that said something about dating site or something
**: Okay. But you have not... (respondents cut in)**	R8: No, I have not heard about that one<br>R9: On radio she used to say it on radio (Both respond simultaneously)
**Apart from... (background noise) (Is there any other place or activity or program that concerns mental health amongst people living with HIV)**	All respondents: No. At all
**Okay. Thank you.<br>(Since you all responded no, I want to know, what do you think in your opinion, is there anything you think we can put in place or do to improve mental health amongst people with HIV. I don't know if you people understand me? Is there structure you think we can put up here to make the mental health better. I don't know if you understand. Do you understand?)**	R7: Number seven
**(Laughs) Number seven**	R7: You can look into the lab fee, five thousand five. It will help our mental health to improve
**(Laughs) to improve**	Silence

**Number nine**	R9: (Another thing is that, you should tell people to stop isolating us. They see us as people that will die soon)
**(who are those that are isolating you?)**	R9: (Ehh, those that feel that they are not infected with the sickness, in this place)
**Here or ....**	R9: (Here, here. Some of the doctors and nurses talk to us and treat as though we are not human beings. It affects our mental health)
**Number two. Thank you so much. Number one (laughs) sorry number two**	R2: (I want to thank the people that organized this meeting, this meeting made me happy. What I want to say is that, you people should try and get a department here and get kindhearted people to attend to us. For example, during the covid 19 period, there was a day I forgot my nose mask. The nurses shouted and spoke to me in a demeaning manner. Please it will be better for all of us if mental health became a department here)
**(Thank you) Number one**	R1: (Number one. Just like what number nine said, a solution is needed. Most of the doctors and nurses avoid us. Some of us got HIV while giving birth or taking injection. It's not only contracted through sex. Two, that's why I am not in any support group. There is something that government gives.... Old people came from far to take the drugs and run test. The leaders of the support group embezzled the drugs and when the older people came, they heard that the drugs had finished. If only they will help as they should, they should also contribute with food and little token)<br><br>(everyone claps)

**(What I want to ask about the support group is that, is there anyone here that attends this support group)**	R1: (There was man that came to look for patients that will join the support group. He said that the government will shortlist me for free drugs. I told him, I don't want any support group and that I bought my drugs myself without waiting for any support group)
**(The person wanted you to join the support group?)**	R1: (He came to me asking if I was aware of the support group. They do this and that)<br><br>(background noise)
**(Does the person work here in this facility)**	R1: (I don't know where he came from)
**Okay**	R6: Number six. One thing I want to talk about is time. Some of us are public servants and civil servants. Like me, I told my officer I want to rush somewhere and come back and I have been here since eight o'clock. If you can help us, to adjust the time to favor us in our works. Thank you
**Thank you. Thank you**	(cuts in) R1: Finally
**Number one. (Background noise)**	R1: (If you come early, you are meant to go early whether you work in the office or you sell goods. But we will come here, and the nurses and doctors waste our time. If we come early, let the health workers start work on time. Eight o'clock is the time for resumption)
**What time do you want them to resume?**	R1: (They should start by eight o'clock. When we come, let them attend to us so we can go)
**(Thank you. Is there anybody that has anything to say about structures we need to put in place to improve mental health amongst people with HIV)**	(Silence)

**(Or have we finished all we want to say? Thank you, thank you, thank you)**	(laughs in the background)
**(Since we have finished the questions, we want to ask you, does anyone have a question or comment for us?)**	R1: (inaudible speech) (pause)
	R8: Me, I go say for like this mental issue now. Why una no.... because of the stigma, una go fit still create awareness at least, you know say no be everybody know about HIV. Some people know but they dem no know say the thing no dey fit like...<br>I fit siddon with you, e no go fit pass through, so they fit say if I siddon with you, I don give you am or maybe a cough (coughs) I don give you am.<br>At least una go still create awareness because, I no fit dey like this now, just siddon freely. Immediately that person don see thing for my hand, he don run. So una go still create awareness too
**Thank you, thank you. Number nine**	R9: There's something, I have been thinking about. There's a family I know, the woman doesn't have HIV but the husband does. They have children, still, the woman and children don't have HIV. How did that happen?
**(I will answer your question but the one that concerns mental health is over.)**	

Transcript of FGD 2 with women caregivers living with HIV, Facility X

INTERVIEWER	RESPONDENT
**(So, I can record right). So, the first question is eh (since we got to know of our status and started coming here to collect drugs, how has it affected our mental health) since we started taking... since we were told our status in this hospital, how has it affected your mental health? Did you get it?**	R1: Okay
**How has it affected your mental health?**	R1: Okay since I noticed my status and ehh since I started taking my medication, I noticed that whenever I took the drug, my brain will be... it's affecting my brain. I will be reasoning abnormally. So, that's the way it's affecting me
**How does it... ehm I just want to know, how do you reason... what do you mean by saying you reason abnormally?**	R1: That's uhmm once I take the drugs, my head will be like, is it compacting or what? I don't know the English I'll use. It will be doing me somehow. Then I complained to the doctor and he said the medicine is a hard drug, that's why it's doing me like that. That I have to change my drug, that I have to change the medicine. Which they have already changed for me
**So ehh apart from the drug that you're taking hmm, let's leave the one that the drug is doing you**.	R1: Okay?
**How has your mental health been since you knew that, you are positive?**	R1: Okay. At times I get angry easily, maybe a little thing I will get upset. You understand?
**Okay?**	R1: That's what I noticed
**So, who else has something else to tell us (anybody else)? Who else has something else to tell us. You know why I'm speaking Igbo... English, because one of us is not Igbo. But if you still want to talk to us using Igbo, please you are free.**	Silence
**Number? Number 6. Okay number 6.**	No response
**(So does anyone have any question or contribution). I asked since you knew about your status, how has it affected mentally, emotionally, how has it**	R2: It's not affecting me anything, because it's only first time wey I take that drug, I feel somehow that day. I reach like that three days I cannot get up. It's only the first time

**affected you? See eh, this thing is not... we need your (interrupted)**	
**I'm not really asking about the drug, what the drug is doing to you. I'm asking your own, when you knew that your status now, you are positive. How has it affected you since you knew it... from the first time you knew your status?**	R2: Only the first time
**What did it do it you? That's what I want to know**	R2: I vex
**That what?**	R2: I vex that time, but all everything, doctor later... I'm not happy that time. Doctor call me back, talk to me. Talk normal thing for me before I get myself
**Who else? Number 6**	R6: I was like my life is ending
**Number 6 abi?**	R6: Yes. I was like my life is ending. I was like my life is no more... there's nothing, no value again
**Okay?**	R6: I was not happy
**But now, how are you feeling?**	R6: That's I'm still feeling like that, but not much
**Okay, okay. Who else has something to say? Number two, do you have anything to say?**	R2: No, no
**Okay. So, then I want to ask? (inaudible speech) So, I want to ask eh, apart from these things that we have discussed, is there another person that want to share something with us? Since you knew about your status, since you knew about your status, how has it affected you? Apart from being angry and the vex, is there another thing it does to you? Because these are the things we need to know.**	R6: I don't want anybody to notice that I have such a like that. Because anytime, any day I don't people to notice...
**You don't want people to know what?**	R6: My status
**Okay, so it makes you to hide or what?**	R6: To hide, to feel somehow

**To feel somehow. What do you mean by feeling somehow?**	R6: Like people knowing my status
**So, what else, what else (is there no other person?) So, are you saying that since you know your status, apart from these ones you've said, there's nothing else that you've experienced?**	R1: Another thing is that... (Interviewer cuts in)
**Number 1**	R1: Okay I'm number one. Once I stopped taking my medication, I stop... I start emaciating. And ehh, you know some people used to hide, even to come and take their drugs they will be hiding. Me I doesn't hide because I don't see as a bad thing. It's a normal thing that can happen to anyone. You understand. So, I used to come around to take my drugs, my medication and that's it.
**So, okay thank you. So, there's nobody else? (there's no other person that has...) It mustn't be English like I said (If you don't know how to speak English) you can put it in Igbo, we will all translate it (you understand). Because I'm speaking English, it's because of our friend here. So (if there's anything you want to tell us) and you want to speak it in Igbo, feel free to tell me, so that we will speak it in Igbo. (I speak Igbo, I'm an Igbo person). The most important is let's discuss cause (this discussion is very important to us, so there won't be any complaint from anyone). In short, I have discussed this with other people. So, it's a group of people that I'm doing this thing so we understand what is the issue. So, number 2 (what do you have to tell us) since you were told about your status. The first you were told, you didn't feel anything, even till now?**	R2: No, I didn't feel anything
**You are, okay?**	R2: yes
**Number 4, okay**	R4: (I didn't feel anything. Just that sometimes I feel guilty)
**Okay**	R4: So that's it. I don't feel anything

**Sometimes you feel guilty. Why do you feel guilty? (what makes you feel guilty)**	R4: Because this kin thing as in (I wasn't expecting it) eh ehn. So, and again sometimes (I feel like my husband is always angry with me so I was like if I had known I wouldn't)
**Marry him?**	R4: Yes, yes
**Okay, alright. Thank you. (please does anyone have anything to say?)**	R6: what I have to say is that.... (interviewer cuts in)
**Number what?**	R6: Number 6.... is that, I always feel like my life will depend on taking drugs every day, that when will this thing, taking drugs end. That I don't want to continue my life by taking drugs all the time
**Okay**	R6: So that's how I always feel
**Okay you always feel?**	R6: When will I end taking drugs
**Okay**	R5: I'm number five
**Number five, okay.**	R5: The first time I heard about my status. I was not happy. After while, I saw a doctor and he gave me some advice. Since then, I was calm and taking my drugs normally
**But since then, you were calm. Sometime do you feel that emotional (respondent interrupts)**	R5: I don't feel it because I know it has happened; it has happened so there's nothing I can do.
**Alright. Thank you, okay. Do you want to say something?**	R: No, I don't have anything (faint voice)
**Okay, okay so I want to ask, in our facility here, how frequently do our healthcare providers make enquiries about your mental health?**	R: How what?
**How frequent? How? when you come? Do you we?... every time you come, hmm, do we make enquiry.... do we ask you people about your mental health well-being, when you come to us, when you come to see the healthcare providers in this health**	All: Not at all (in a faint voice)

**facility? Not about your BP or fever or anything?**	
**Do we?**	R: No
**Okay, Number?**	R6: Number six. Not at all
**Which other number that wants to say? Number?**	R2: Yes, yes
**Eh? please be audible**	R2: Yes
**Who asked you?**	R2: Who asked me?
**Eh yes have they ever asked you about your mental health. Like this type of this thing... yes that we are talking about**	R2: It's one of my sister
**No, here?**	R2: yes, yes, I know. One of my sister asked me. She's nurse
**The healthcare provider?**	R2: Yes
**So, what did you people discuss. Let's know, share with us**	R2: We discussed about normal thing like that. We just.... (sighs)
**Say now, you are here to help us to get better**	R2: No problem
**Eh?**	R2: (laughs) it is well
**Which one is well, nne. So, let's continue, I asked a question, you people said what again? Eh ehn you said that you people did not discuss... what did you people discuss? Just one or two**	R2: she just advised me how I will take live my life. How I will take.. so that I'll live long.
**Okay**	R2: It's the only thing me and she just....
**But did you complain about anything before she asked you?**	R2: Yes, because the first time wey I enter here, it's only her me and she meet. So, we sit down that day and discuss, discuss. I talk that day, I really shed tears before she now put me for normal (interrupted)

**Is it okay to just tell us one or two things that you felt that made you shed tears?**	R2: Because I don't believe say I go get this kind sickness
**Thank you thank you. So, you people said no, that most times they don't ask you about your mental health?**	R5: Five. Nobody has asked me such question.
**Please if we are talking, let's raise our voice so this thing will be able to hear us well. Please oh. So, let's ask now. Based on our experience now, hmm, based on our experiences now, I want to know what are the things, what are the factors? (Pause)**<br>**So, I want to ask, this is our experience. It may not be your own, it may be that you have been discussing with your fellow women. We want to know, what are the things that you feel, factors, what are the problem, what are the issues that can affect our mental health. Did you get? She's a doctor, Dr []**	Dr. []: Good afternoon
**Hmm. So, what are the issues, what are the things that happen in our life. I will be speaking English and Igbo (What are the things we go through that can affect our mental health?)**	R1: A divorce can
**Eh?**	R1: Divorce
**Divorce?**	R1: Yes, can affect someone's mental issue
**Divorce (How?) how?**	R1: Marriage. When you are into marriage. Divorce can affect mental issue. When you are thinking what

	you don’t supposed to think, it can your mental issue as well.
**Okay. Apart from that, what else do you feel as young mothers, are the things that can .... we are living in Nigeria, we are you know, everything, not about drugs. We are looking at it from our health facility, we are looking at it from our family. Do you get what I’m trying to say?**	No response
**We are looking at it from everything. What are the things that you feel you are facing, or your friends that are taking these drugs, that are also your agemate, that you interact with. Based on their experience that you found out that these are the things that are affecting their emotional health or mental health? Do you get?**	(background noise)
**Number?**	R6: Number six. When there is no money to take care of yourself enough. You are (interrupted)
**When there’s no money for you to do what?**	R6: To take care of yourself
**Okay?**	R6: And feed the family
**Thank you**	R6: It affects mentally
**Okay. Thank you, thank you (Please any other person?). Who else? You wanted to say something?**	R1: I have said it already
**Please, we are all in Nigeria, we are all here. We are not asking about... I’m just asking generally. What are the things? Everybody goes through mental health issues, one day or the other. It’s not that it’s meant for some people. So, I’m asking this generally.**	R1: Another thing, I’m number six

**Number one**	R1: Number... okay, number one. Another thing that can affect our mental health issue is lack of communication. When you don't communicate with your loved ones, maybe you feel lonely. It can as well affect the... the health
**Okay**	R1: Yea, the mental health
**Thank you, thank you. Number one. So, in our heath facility, is there anything that, when you come here, that will affect your mental health? Let's start from health facility. Is there any health facility thing you know in our clinic? Hmm? Number?**	R6: I'm number 6. Like as we came now and our folder have not been found since when we came. So, it affects us mentally
**Thank you. Number six, thank you. Who else?**	R1: You mean in this clinic?
**Yes, is there anything that affects your mental health?**	R1: Okay. Like here as we are now, there's no light. Somebody like me, I feel disturbed when I stay in a place where there's no fan or AC (background noise) Yes. I feel disturbed
**Okay, okay. Number 2. (There's something) that you know, once this thing happens, you are not yourself again. You just feel no... no... no... this thing has really affected... this has really affected my mental health. It makes you worried, it makes you sad, it makes you something, do you get?**	(No response)
**What are the things? These are way to... you know, so we know and help. (If you want to speak in Igbo you speak, okay?)**	R2: I don't want to talk anything
**(Even the child you are taking care of), is there not anything that you know that, (that can affect your mental health)? Hmm? (you don't have?)**	R2: (No, I don't have)
**What of you, number 3?**	R3: Number three. Now I'm feeling bad. The first-time wey I start for here, they tell me to do antenatal,

	do everything. I do everything. Later on, they come tell me say it's not by force to do antenatal here, you can do antenatal for anywhere wey you live. So later the first one, the second one, I no come do antenatal here, I come do antenatal for another place. The day I wan come collect this viral medicine wey I will give my baby, they give me. But this one now, last time I suppose to collect that thing, they say no, say till I go where, register where, register where. I don't know... I don't know
**Okay, so the stress of you (background noise) of you getting drugs (respondents interrupts)**	R3: I suppose to collect that thing. I no suppose to enter motor and come here again but they still make me enter motor and come here again up to still
**Still, they've not given you. Thank you, thank you so much.**	
**Do you have anything to say?**	R4: Hmm nothing
**Even if it is family, anything?**	R4: It's just the stress is much
**Stress in terms of where now?**	R4: In terms of file. They will ask you go there, go this place. Before they will now take you to doctor
**Okay, thank you, number four**	R4: Ehh number four
**(Do you have any family issues, outside this health facility. We have heard of health facility now). I'm now asking outside here, is there anything outside that affects your mental health, that you want to share with us and you feel we can help you from there. Because the idea is to share. Did you get what I asked? Is there any mental health issue... family issue that you feel you can share with us?**	R: No, I don't have anyone
**Hmm?**	R: I don't have any<br><br>R6: Like at times when my mother will start talking about my status
**Number what?**	R6: Number six

**Okay six. Thank you**	R6: Talking about my status
**Your who?**	R6: my status... my mother
**Okay, okay alright**	R6: It affects me
**So, it affects your mental health? Alright thank you. So, let's go.... so, what else now? Are we...? Is there anybody that has anything... anything to say in this number? In this question?**	(muttering sounds)
**Is there anybody that have any other thing to say? Are we okay with this one? So, okay, I want to ask. We are almost... we are through with all these things. So, do you know of any program? Or will I say, activity here that we do, that takes care of your mental health? That.... number what?**	R4: Number four
**Number four. Okay what do you want to say? You said something, I didn't hear you**	R4: No, I said no
**Is there another person that wants to say?**	R1: I didn't understand your question
**I said eh, do you know of any program or any activity that is ongoing in our facility here? That addresses your mental health issues? That takes care of your mental health issues**	R1: no
**Say number one?**	R1: I'm number one. No
**Number?**	R3: Number three. Like the first time wey I start, they used to discuss, ask question, but now
**They don't do that thing**	R3: They don't do that thing

**They don't do that thing again. Do you why they don't do that?**	R3: I don't know oo
**Okay, thank you. Alright, so do you know of any program here that takes care of your mental health issue? Number what?**	R5: Number five. I don't know of any program
**Okay, you don't know of any program. Okay thank you. What of number six**	R6: Number six. I did not... I did not know
**Okay so, do you?... what... what... The final one. Since there's no program here. I just want to ask, do you...? What are the things that you feel, that if we put in place here, it will improve your mental health? This... this one now, I need everyone to talk. You said there's nothing going on now, but I now want to ask, what are the things that if you put in place here eh, it will improve your mental health well-being? Number three**	R3: You know say sometime.... (Interviewer cuts in)
**Please increase your voice**	R3: .... person will feel somehow from him house but when you reach here, when they discuss everything. Before you know, you get yourself before you go back
**What are the things you mean by discuss everything? What are things you want us to discuss?**	R3: You know say like before, if people normal to averse. When we reach here, you people will talk to us, do everything before we collect drugs
**What are those things? Do you know them? The ones you listen to? What are those things? So, we know**	R3: (laughs) Like before you people used to preach for us. Talk to us, do this, do this before people collect drugs but now, they don't used to do it again.
**Okay. Thank you. thank you, number three. Please number two, what do you want us to do to this place? Or are you okay with the way you are seeing us here?**	R2: (No response)

**I2: Feel free to air your views**<br><br>**I: I have told her oo. I don't know how else to tell her**<br><br>**I2: Feel free**	(silence)
**You don't have?**	R2: I don't have anything
**Okay number one**	R1: I'm number one. The things I want you people to put in place, if they can start the teaching again. Eh ehn the teaching, then the advice. You people should be advising us on what to do, on how to go. Especially we, the mothers, how to take care of the kids, the newborn eh ehn. I think it will help us
**Thank you, number one. What of number six? Is there anything you want us to put in place in this our health facility that will improve or address any mental health issues that our women face?**	R1: What I want to put in place is that, anytime we come, let them attend to us.
**Okay, you want us to attend to you?**	R1: To us quickly as we came. Anytime we come
**Okay thank you. You want us to attend to you?**	R1: Quickly
**As you come. Okay thank you. So, number?**	R5: I' m number five
**Okay**	R5: What I want to put in place is that, if we come here to collect our drugs. At least let's have people that will be giving us word of encouragement
**Okay?**	R5: Eh ehn. So that at least it will make our health to be more stronger. Thanks
**Thank you. I think we have come to the end of this. Does anybody have any problem, any comment, any question to ask us?**	(silence)
**To ask me**	R1: I need to ask you question? My question is this, someone that have this issue? Is there any diet that

	the person will be placed on? Or can the person be eating anything?
**Diet?**	R1: Like food
**Like what do you mean by if someone has the issue? Which issue? Is it? Let's understand**	R1: This our status now
**Okay this your status**	R1: Yes. So, is there any diet or food that the person will be placed on? That will be helping to... is it to neutralize the issue?
**Okay, thank you. I will answer it. I'm coming. (Does anybody else have any question?) No. Okay, thank you so much.**	

Transcript of FGD 1 with women caregivers living with HIV, Facility Y

INTERVIEWER	RESPONDENTS
**(I want ask you. Since you got know of your status, how does it affect mental health? Since you got to know, how does it affect you mentally? I don't know if you understand what I asked you?)**	All: No
**(You didn't understand? I will repeat the question. Since you knew about your status, and you started collecting drugs from here, how does it affect you mentally? Have you understood now?) Should I ask it again?**	(Inaudible response)
**(Inaudible speech)**	(silence)
**(Do you want to say something mummy). Number nine. Go ahead**	R9: (I have been coming here since 1991. It's not been easy on me mentally. I thank God that I started coming here, if anyone tells me to leave park lane I won't do it. Since I started taking my drugs, I have been doing okay. Nothing is really wrong with me. The reason I came here is that I was told to come and speak up. I like what is going on here. Well done)
**(I heard you, but I'm not asking about this meeting is doing for you. What I'm asking is that since you started coming here, how has your mental health been? What I am asking is since you started collecting drugs, even before the support group, how have you been mentally? It's not about the support group)**	R9: (Is it whether I feel sick or if I'm feeling alright?)<br><br>(background noise)
**(We mean within you; how do you feel?)**	R9: I still don't understand
**Number?**	R3: Number three. When I got to know of my status, I felt very angry. Before now, there are some kinds of job I am able to do that are strength consuming, but since I started taking these drugs, I get tired easily. Now, I am not able to do those kinds of jobs and they are the ones that pay more money. Also, I am not able to go to places I want to go because my last son is not old enough to take care of

	himself. I can't be open in places about my status for fear that people might laugh at me, or run away from me. If I can be free of this illness, I will be able to open up to people, achieve a lot of things. That's what disturbs me. Thirdly, my husband who was very supportive is late. The loneliness is too much. If my husband was still alive, he would have helped. Now, I do everything alone. Whenever I think of the fact that I am not feeling well or I am taking drugs or my husband is no more, I feel sad at times. The drug helps but there are sometimes I feel overwhelmed. That's all I have to say)
**Thank you, thank you.**<br><br>**I2: (Does anyone have anything to say concerning the question we just asked)**	(no response)
**(Anyone? You know how it affects you. Like we said, this is a personal experience. There are no right or wrong answers. We explained this to you when we started. Please feel free to speak to us)**<br><br>**I2: (Anyone that what's to speak in English should please speak, if you can speak in Igbo, it's still okay. If you want to speak in both... Interviewer one cuts in)**<br><br>**I: (Everything)**<br><br>**I2: (Fine, we understand everything)**	(silence)
**(Does anyone want to talk)**	R1: (What I observed since I started taking this drug is that, I feel weak in my body all the time. I ask myself if it is the drug that is causing it. I started taking this drug since 2008. When I look at myself, I conclude that it is the drug that causes the weakness. This is something I haven't experience before)

**(Thank you. Does anyone have anything to say? Does anyone have anything to say? Nobody?**	(No response)
**Mummy do you have anything to tell us? Tell us. Number what are you?)**	R5: I am number five
**Number five**	R5: I have known my status for a long time. I got really angry. I felt like my life was over. Everyone, including my children were wondering why I was always angry.<br>I thank God. It was when I started coming here to take my drugs that I started seeing people like me, I started going out. Then I calmed down, started doing things as instructed. My husband and children have been very supportive. That was my experience. I got rid of all that anger and issues I was having and became very active)
**(Thank you, thank you. Does anyone have anything to say. Number seven)**	R7: When it started, I didn't know. I kept falling ill. In my place of work, a doctor asked me what was wrong with me and I told him I didn't know. Finally, I got to know. The doctor there told me that this illness doesn't kill anybody but that I should take my drugs as instructed. Since I started taking these drugs, it doesn't affect that much anymore
**(We want to go to the next question so does anyone want to say anything? Okay. Let's go to the second question. This question is, do the doctors and nurses ask you about your mental health? Do you understand what I asked?)**	(silence)
**(I said since you started coming to this facility to drugs, how often do the healthcare providers ask about your mental health?)**	(No response)
**How frequently... how frequently (how many times do the healthcare workers ask you about your mental health? It is not about your drugs, it's not about other things, it's about your mental health. I don't know if you understand what I am asking you?)**	R4: Number four

**Number four**	R4: Anytime I have an appointment to see the doctor, the doctor asks me how I am faring, if I am having fever. If I have, I will tell him and he will instruct me on what to do before I go. If I treat myself, I will recover
**I: (Thank you)**<br><br>**I2: (What we are asking, is if the doctors, nurses or counselors that you see here, take time to ask you about mental health or ask you if you are having suicide thought, or angry or thinking too much. It's not just about fever or cough and so on. That what we want to find out from you)**	R4: (Number four. They have not asked me about me mental health but they ask if I feel pain in my body and if I do, I tell them where I feel it)
**(Thank you. Thank you)**	R3: (The only time I meet with the doctor is if I have a problem. We don't always have the opportunity to see them. It's only when we have a problem that we see them, they ask what our problem is and we tell them.)
**Thank you. (Does anyone want to say anything?**	(No response)
**(Does anyone want to say something?)**	(No response)
**Number nine**	R9: I'm number nine. I see them when I run test, to know my viral load. They tell it's okay and I am doing well. They also ask if I drink alcohol and I tell them no)
**(Thank you. Thank you. Thank you. Does anyone want to say something)**	R3: (I am number three. There are people that work in this hospital that take care of us. They call us on phone to ask us about our health care. They call me once in two weeks, sometimes they visit me in my home to check up on me. You know the doctors don't really have time to come to our support group to ask us questions. It's only when we feel sick that we see them and they tell us what to do.)<br>Thank you
**(Thank you. Let's go to the next question. We are almost done. Please I want to plead**	

**with you especially those who have not said anything. I would like us to contribute to this discussion. This information will help us)**	
**Based on your experience (background noise) (Based on your experience, what do you think are the things that contribute to your mental health issue? I don't know if you understand my question)**	R: Yes
**(What are your thoughts? The way you understand it. What do you think can contribute.... It may not be you. It may be your friend, or anybody. We want to know what contributes to the mental issues of the patients in this facility. Mummy did you hear my question?)**	R: Yes
**Number one**	R1: Number one. From what I understand, what causes it, is when things aren't going the way they're supposed to. You are not happy and you don't have peace of mind. You become very disturbed mentally. But if you are mentally okay, you can do things on your own)
**Thank you, thank you. There's someone that raised her hand)**	R4: Number four
**Number four**	R4: What can affect someone mentally is overthinking. The problem we go through can affect someone mentally
**(Thank you. Number three. You want to say something)**	R3: When it comes to mental health problem, just like what my sister said, when things are not going well for you, When I think of the drug, I take all the time, it makes my heart beat fast and it gets me angry.<br>When things are not going the way, they are meant to and you are not physically fit to do thing you want to do, it makes someone very upset.)
**(Thank you. Number two, is there anything you want to tell us?)**	R2: (What I want to say is that), in a situation whereby you are looking for a job, you reach

	there and they ask you for your status and all those tests and it happens that you come out positive. It happens that they will not take you because, they find it difficult to take people on such status.
**Thank you. Thank you. Is there anyone that wants to say anything? Number six**	R6: Number six. (What I understand is that, for example in your family, your husband doesn't have HIV, he isolates you and your family is not financially stable. When you remember all these things, you remember how your husband treats you badly, all of these things can affect your mental health. The things you are meant to do for your children, you can't do it. You aren't strong enough to fight for yourself more or less your children. Then you remember your condition, your husband, the condition of your children. It can affect you mentally.
**Thank you, thank you. Does anybody else have anything to say? So, we can move to the next question**	(silence)
**Does anybody else have anything to say? Number eight, do you want to say something?**	(No response)
**Number ten, do you want to say something**	(silence)
**Okay. The next question is, do you know of any program or activity that helps to address and improve mental health among HIV patients in this facility**	R6: Number six. There's a meeting that holds every Saturday in a month. When you come to the meeting, they address things like this. Instead of overthinking, the things they talk about in the meeting, helps to encourage us down. We also get to meet people like us which is comforting including the counselling too.
**I: Thank you**<br><br>**I2: Can you tell us the name of the meeting?**	R6: We do... (second interviewer cuts in)
**I2: I mean the name. What is the name of the meeting that holds every Saturday?**	R6: (sighs)

**(Both respond) Hope support group. Thank you**	R6: Yes, hope support group
**Thank you**	R6: Another thing I want to say. Anybody that attends the meeting will not be overburdened because we learn a lot from the meeting and we get encouraged too. We benefit a lot from the meeting more than people that don't attend. Anybody that attends the meeting will not have much problem
**Thank you. Is there any other activity apart from this one that was just mentioned? Is there another one you can remember that helps? Or is there anyone in this facility that helps to improve our mental health?)**	(no response)
**(Thank you, thank you. You said the hope support group helps to improve your mental health. There's something we will like to ask you. What can you do to get people to attend this support group that helped you to improve your mental health? We are all women. What do you think makes women to not attend support groups?)**<br><br>**12: What hinders them from coming? (first interviewer cuts in)**<br><br>**I: ... hinders them**	R3: (I am number three. I think there are two things that hinders people from attending this meeting. There are some people that are shy. They wouldn't want to run into people that know them. The second one could be that the person doesn't have transport money to attend or come for the meeting. Like me, I do well in the support group. I attend with my child but I haven't been able to attend this year because I relocated from [mentions location] to a farther place. I take four drops before I get to this place. I told them they should fix the time so it can be convenient for me and my child to attend. These are things can hinder someone from coming to the support group. Thank you)
**I2: (Thank you. Does anyone want to say anything concerning the things that can hinder someone from coming to the support group? There are other things, like number three has told us about stigma or shame and cost of transportation. That is transporting ourselves from our houses to the hospital or support group meeting. Are there other things we can remember that**	R7: (Number seven. There are some people that get prepared to attend but won't have transport money while some may not have chance to attend. There was a woman I reminded about the meeting, but she said she wouldn't have time because she was going to the market to buy foodstuff for her family)

**can hinder people or hinder you from coming? That is what we want to know apart from the two points that number three has told us)**	
**(Thank you. Does anybody want to say anything? Number two. Mummy is there anything you want to tell us?)**	R2: (It's the same issue about transportation)
**(Transport money)**	R9: There's somebody I told to join us... (Interviewer cuts in)
**Number nine**	R9: (Number nine. There's somebody I told to join us in our meeting. She asked what kind of meeting it was. I told her it is a meeting for our health and we all have folders here. She said she won't have chance and transport money is much for her. So, we thank you for receiving us)
**(Thank you. Thank you)**<br>**(Background noise)**<br><br>**I2: (Is there anything to remember?)**	(silence)
**I2: Number?**	R3: Number three. I want to contribute to the conversation. I came from [mentions location] before coming to register here. The way they do it in [mentions location] is that if you don't come to the support group, you will not be given drugs. There's a paper you will be given in the support group that will be used to collect drugs in the pharmacy, without that paper you will not be given drugs. So, that makes a lot of people to attend the support group. I don't know if you people can do something like that so that people can begin to attend.<br>Another thing is that everyone that come to the support group goes home with transport money. The amount is you get depends on your where you are coming from. It motivates them and people are always happy to come to the support group

**I2: (You have started answering our next question. What do you think is the solution to the challenges that hinders people from coming to the support group meeting or coming to the hospital to take things that can help with their mental health. We have heard from number three even though she's welcome to contribute more but we will like to hear from others. What do you think we can do to encourage people to come to take treatment or attend the support group meetings. We will like to hear from other people too)**	R7: Number seven
**I2: Alright**	R7: (Before, people used to attend. Sometimes they give us rice or make abacha for us to eat. Since they stopped doing those things, people stopped coming)
**Thank you. Number ten**	R10: (What I think can encourage us to come, is to give transport fare or give us something to eat. When people remember that they'll be given transport money, it will encourage them to come)
**I2: (Does anyone want to say anything?)**	R7: (When some people think of what is involved, they prefer to look for what to do to help take care of themselves.)
**(Alright thank you. Now that we are done asking you questions, does anyone have any question for us to answer. Any contribution?)**	R4: (Number four. Please we need you people to assist in any way you can, so as to enable us attend these meetings)
**Any other person? Number three**	R3: (Number three. I support what my sister said. If there's a way, we can get assistance just like I said before. There are some jobs we apply for, once they hear of our status, we get disqualified. We need your assistance in securing jobs that we want, even with our status. You can also assist us financially, especially people who run businesses. This can encourage people to start coming)
**(Does anyone have anything to say? We are rounding up)**	(silence)

**(Nobody? Okay. Thank you everyone.)**	

Transcript of FGD 2 with women caregivers living with HIV, Facility Y

INTERVIEWER	RESPONDENTS
**(Since you got to know about your status, how has it affected your mental health wellbeing? I don't know if you understand what I'm asking you. Since you got you know that you are HIV positive, how did it affect your mental health well-being? Did you understand?)**	(Inaudible response)<br><br>R5: Can I speak English?
**I2: Number five**	R5: Can I speak English?
**Number five. (Any language you want to speak, feel free)**	R5: Actually, at first it was a real depressing emotional something. So, but with time I got to realize I have to live for myself cos my mum wants me to live. I have to live cos I have a child to take care of. Then at that period, doctors were able to talk to me, telling me that as long as I am taking my drugs, I can be whatever I want to be. So that's how it started and that's how I have been going since.
**Okay. So how has ... how did it... you said that when you started it affected your emotional... (respondent cuts in)**	R5: Yes
**So, what were the things that you noticed then?**	R5: I keep asking myself. Sometime I seat on myself, I was asking myself, how do I...? how did this thing happen? How do I get to...? like how did I find myself in this situation? Sometimes I lock myself in the house like three days, I am not out. My mum keeps getting worried and was like telling me "It's not your fault", because then we got to find out that my

	parents doesn't even have it. Then, when we got to find out what... I found out when I was fifteen years. Then, my mum had to realize that it was the blood transfusion I had when I was five years. Because, it really affected me in a point that I have to start vomiting out blood, stooling blood, urinating blood. So, that was the kind of depressing action that this thing got to have me on. So, my mum told me that it's not your fault, you don't have to blame yourself, you don't have to blame anybody that this actually happened. So, words of encouragement from my mum and from the doctors, I get to find myself out of the depressing character I found myself in
**I: Thank you. (Does anyone have anything to say? We can speak Igbo) Number two**	R2: Number one
**Number two (laughs)**	R2: (I am number two, okay. Since I started drinking this drug, it didn't affect me so much, I do things that I want to do, I go to anywhere I want to go, as far I take my drugs.)
**(Okay. I am not asking about the drugs, what I am asking is, when you got to know you were HIV positive, how did it affect it you? How did you feel mentally?)**	R2: (I didn't feel bad so to speak because I have heard about the drugs and if you take the drugs, you will be able to live longer. So, I began to drink my drugs.)
**Thank you. Number one**	R1: Number one. When I first find out that I was positive, it wasn't easy. I thought of killing myself. It got to a point; my HIV status was all I was thinking about. I found out about my status when I was pregnant. It was so bad

	that I lost the pregnancy. It affected me badly, I won't lie. It wasn't easy. Whenever I remember those times, I get so upset. I haven't been able to recover till now. Though, I have been taking the drugs for a long time now.
**I2: Like how many years?**	R1: about six to seven years now. (It's not been easy for me. Whenever I remember I take drugs every day, I get sad. It's not easy. Thank you)
**Thank you. (Does anyone want to say something)**<br><br>**I2: Number six**	R6: When I found out, I cried so much. I went through a lot then. I didn't expect that something like this would happen to me. I didn't recover even after a month. My relationship didn't work out. I get panic attacks up till now. I suffered low BP two months ago and I nearly died. I thank God that I am alive today.
**Thank you. Number three**	R3: I have been taking this drug for eight years. It started out as headache, then fever. When I got to the hospital, I was told that, I was positive. I felt like dying, but when I saw other people like me, I became strong. Now, it doesn't affect me that much again. I have given birth to three children safely.)
**Thank you, thank you. Let's go the second question. How many times do the healthcare workers in this facility ask you about mental health? I don't know if you understood the question**<br><br>**I2: How many of them ask about your mental health well being**	

**(When you come to then clinic, or you bring your child, apart from collecting drugs, how many times do the healthcare workers ask you about your mental health?)**	(silence)
**(Do you understand me the question I asked?)**	R4: Number four
**Number four**	R4: (Since I started coming here to take my drugs, since I got married and gave birth, nobody has asked me about my mental health. I don't know about others.)
**(Thank you. Any other person? Number six)**	R6: (They do ask me how I feel in my body, if I react to the drugs but I tell them I don't react to it. When I started taking it newly, around seven in the morning, it affected for the rest of the day. I complained to the doctors and I was told to eat before taking the drugs)
**I2: (What of your mental health?)**	R6: (They do ask me)
**(What do they ask you?)**	R6: (They ask me how I feel in my body especially as a woman. I ran test but I can't remember the test. Thank you)
**(Does anyone have anything to say? There's something....) (respondent cuts in)**	R2: (Number two. They do ask us. They have asked me if I take my drugs, how I feel, and I tell them that everything is okay. That is, it.)
**(But they have never asked you about your mental health?)**	R2: (Hmm. If they ask you and you have any complaint, you tell them)
**(Okay thank you. Another question I want to ask, based on our experience,**	R2: (What can cause it is....) (second interviewer cuts in)

**what do you think contributes to the mental health problem among women)**	
**I2: Number two**	R2: (Number two... is if the person thinks too much about her condition. That can cause it. But if the person accepts the situation and focuses on her health and take her drug, it won't worsen it.)
**Number four**	R4: (Number four. Overthinking can cause it, when you don't have what to eat. If you are able to eat and take your drugs you will be feel strong and healthy. When someone thinks about all these things, it can contribute to one's mental issue)
**(Thank you. Mummy do you want to say something? We are asking what can worsen the mental health problem)**	(silence)
**(Mummy what you have anything?)**	(I don't have)
**(We are all women. What we are asking... (second interviewer cuts in)**<br><br>**I2: (There's no right or wrong answer)**<br><br>**I: (Yes, we are asking about the issues you encounter. For example, coming here to take your drugs, you have children, the economic situation in this country. What can you say affects your mental health. We need to hear the experiences you've had)**	R1: (I am number one. What I think contribute to mental issues is stress. Stress can bring someone down even someone that isn't HIV positive. Stress can kill someone. Only if we can reduce the stress. The condition in the country isn't good but we will still our best as human beings. But we need to reduce stress and also, overthinking can cause it. Once you start thinking a lot, it can lead to stress and it is not good for the body. Your body doesn't need it for now until... in fact there's nothing like until, your system doesn't need stress or overthinking. When it comes to family issues, what you can do is concentrate on yourself. I feel like if we stop thinking

	about these things, it can reduce stress level and everything will be fine)
**(Thank you. Based on the fact that we are taking drugs, does it affect our mental health? As a woman taking these drugs, is there anything you think can damage our mental health?)**	R1: (What I think can damage our mental health is exactly what I said now, overthinking and stress.)
**(Okay thank you. Does someone want to say something? Number?)**	R2: (Number two. Number two. Another strong point is the situation of the country we live in now. It is serious. If we can get financial support, it will help a lot and make things a bit easy. That's one of things that affects our mental health)
**Okay, thank you**	R4: Number four. Another thing that can worsen it, is the condition of the country. Sometimes you think, with the way things are going, they might stop bringing these drugs into the country. What are we going to do? That means we will all die. So, it is one of the things that causes it
**(Thank you thank you. Let's go to the last question. I want to ask; is there any program or activity we know in our facility that takes care of our mental health? Do you know of any one?)**	(I don't know of anyone)
**(Is there anyone you know of?)**	(I don't know of anyone)
**(Is there anyone you know of?)**	(Hmm there was one that aired on radio but it has been long. In 2020, they were talking about people who take the HIV drugs. They said that there was a drug that they were going to release, that will

	cure the HIV completely. Since then, I have not heard about it again)
**(Is there anyone that talks about your mental health? They teach about....) (respondent cuts in)**	(There's none)
**(.... happiness. There's none? Have you heard of anyone?)**	(No, I have not heard)
**(Since you said you don't know. Are there things you feel we can put in place to improve your mental health. Please we need to hear your thoughts on this. I don't know if you understand me? Since there are no programs in place what do you think we can put in place to monitor your mental health closely.)**	(No response)
**(This is the last question. This is very important because it will help us to know what to do, to enable us help you. Do you understand?)**<br><br>**(background noise)**	R4: Number four. What I suggest is that, you have people that will call us to check up on us, speak words of encouragement to us. It will help us a lot. That's what I feel.
**(Thank you, thank you. Does anyone have anything to contribute? Apart from phone calling, are there things we can do in this facility? I'm asking anything else. Mummy thank you for your contributions. Is there anyone else?)**	(silence)
**Thank you please. It's only you that can tell us how it is, so we know how to help. Number four thank you)**	(silence)

**Number one**	R1: Just like what I said, we don't need stress. If we can get help financially, it will be encouraging, even if it's once a month. It's difficult for some people. Some people come from far distance to the hospital. So, I think that if we get support with the drugs and material things we need, if you people can do that for us, I'm sure this mental stress will reduce
**(Thank you. Anybody else?)**	(No response)
**(Anybody else?)**	(No response)
**(Anybody else?)**	(No response)
**I2: (Do you want to say something? Say something.)**<br><br>**I: (she wants to say something but she doesn't know how to say it)**	(No response)
**I2: (Is there anything we do to reduce the mental stress. That what we want to know?)**<br><br>**I: (So, it will be good for us and good for you too. Is there anybody? Or is there someone that wants to say)**	R: My sister has said everything. We need support<br><br>R: If we can get transport money. For me, I come from very far place. I take like three drops to get here<br><br>R: And we don't see what to eat. At least when we come to collect drugs, we see what to eat<br><br>R: they said we should eat vegetable and fruits. Where did we see the money to buy vegetable and fruits

	(Simultaneous responses)
**Does anyone have anything to ask question? We are done with this session.**	(unintelligible words)
**No question? Does anyone have any question? No question**	(No response)
**I thank you very much for your time**	

Demographic information of FGD participants

Participant ID	Health Facility	FGD Session	Age (years)
13	X	1	24
14	X	1	32
15	X	1	19
16	X	1	24
17	X	1	20
18	X	1	24
19	X	1	22
20	X	1	42
21	X	1	54
22	X	1	22
23	X	2	28
24	X	2	26
25	X	2	21
26	X	2	52
27	X	2	51
28	X	2	21
29	X	2	23
30	X	2	30
31	X	2	26
32	X	2	34
33	Y	1	32
34	Y	1	24
35	Y	1	22
36	Y	1	31
37	Y	1	26
38	Y	1	30
39	Y	1	23
40	Y	1	30
41	Y	1	26
42	Y	1	22
43	Y	2	30
44	Y	2	27
45	Y	2	39
46	Y	2	28

47	Y	2	34
48	Y	2	23
49	Y	2	41
50	Y	2	18
51	Y	2	22
52	Y	2	52
