# Supplementary material for: Exploring barriers to and opportunities for improving mental healthcare access for women caregivers living with HIV in Nigeria: A qualitative study
Source: PLOS Glob Public Health. 2026 Aug 14;6(8):e0005834. doi: 10.1371/journal.pgph.0005834 (PMC13475909; doi:10.1371/journal.pgph.0005834)
Supplement: Supplementary file 6 [file pgph.0005834.s007.docx]

22^nd^ December, 2025.

Dear Editor in Chief,

Please find below the summary of comments and suggestions made by reviewers on the manuscript titled “**Exploring barriers to and opportunities for improving** **mental healthcare access for women caregivers living with HIV in Nigeria: A qualitative study”** and the authors’ responses. All punctuation, typographical and grammatical errors in the manuscript have been corrected.

The corrected manuscript is uploaded together with this rebuttal on the online manuscript submission system.

The comments and recommendations are highly appreciated.

Thank you.

Ugochukwu Uzoechina Nwokoro.

**REVIEWER 1’s COMMENTS**

| **S/No** | **Comments** | **Response/corrections done** | **Reference (page and line)** |
| --- | --- | --- | --- |
|  | GENERAL/INTRODUCTION |  |  |
| 1 | Check the grammar and writing of the whole article. Generally, the writing is good but there is room for improvement. For example, in the abstract (p.2, line 30) the sentence should start “The study….” Rather than just “Study….” | All grammatical errors have been corrected, and the specifically referenced sentence has been revised to now read: *This study was conducted and reported in accordance with the Consolidated Criteria for Reporting Qualitative Research (COREQ).* | Page 2, line 31 |
| 2 | In the introduction on page 4 (lines 81-85) the sentence starting “Recognizing the rising need to integrate mental health…….” is extremely long. It should be shortened or divided into 2-3 sentences. | This change has been implemented, with the sentence broken down into 3 and now reads: *National guidelines for the treatment and management of HIV in many Low- and Middle-Income Countries (LMICs), including Nigeria, recommend routine screening for common mental health disorders such as depression and anxiety among PLWH. This is in recognition of the rising need to integrate mental healthcare services into routine HIV services. However, evidence shows that routine screening for common mental health disorders is not regularly done by healthcare workers.* | Pages 4 and 5, lines 87 to 93 |
| 3 | Introduction: page 4, lines 72-76 discusses “high income countries”. Are these factors not relevant to low resource contexts? You need to link these findings to the context of this paper; for example say if/why they are relevant to low-resource settings, or replace the sentence with more relevant findings. Alternatively, you could remove the sentence altogether as it feels a bit out of place. | Thank you for this important observation. This section has been modified to show the relevance of these factors in low-income contexts. | Page 4, lines 83 to 84 |
|  | METHODS |  |  |
| 4 | I suggest having a paragraph/section on Levesque’s framework (and the table) under study design – it is currently detailed in the section on analysis, so it can be removed from there. The framework informs the whole study so would be better under study design. It can be referred to briefly in the analysis. | Thank you for this suggestion. The paragraph on Levesque’s framework (and the table) has been moved to the study design section as recommended. | Pages 6,7, and 8, lines 125 to 141 |
| 5 | Were FGDs conducted in English? If so state they are (it only says interviews were conducted in English – line 151, page 7). If not, say the language/s and about the process of translation | Thank you for this observation. This change has been implemented, and the sentence now reads: *Interviews and FGDs were conducted in English and recorded using a digital recorder with the consent of the study participants.* | Page 10, lines 181 to 182 |
| 6 | Analysis – there needs to be more details on the process of analysis. For example, how were the data coded? Was software used to help organise and code? I recommend outlining briefly the steps taken for analysis. | The data management and analysis section has been modified to outline the process and steps taken in coding and analysis. | Pages 10 and 11, lines 195 to 202 |
| 7 | Reflexivity is mentioned (which is good), by saying researchers “tried to be aware and transparent”. However, no details are given on how this is done, steps taken to mitigate bias or examine influence. Examples could be regular discussions, debriefs, reflexive notes etc. Please add some details. | Thank you for this observation. Additional details on reflexivity have been included as recommended. | Page 11, lines 208 to 211 |
|  | RESULTS |  |  |
| 8 | “Ignorance and low mental health literacy” theme: please consider dropping the term “ignorance” from the theme title as it has quite negative connotations. You could simply have “Low mental health literacy” or “Poor knowledge and low mental health literacy | Thank you for this suggestion.  This change has been implemented, and the phrase now reads: *Lack of awareness and limited mental health literacy* | Page 15, lines 278 and 279 |
| 9 | “Ignorance and low mental health literacy” theme: this theme appears to be based only on one participant’s opinion (a nurse). Normally for a theme to emerge there should be some level of consensus, or it to be referred to several times. Was it identified by others? Did others comment on this as a barrier? It is important to say if so – and also if not. | Thank you for this observation. However, we decided to bring out some important concepts which are known from literature to be important barriers or opportunities even when they were referenced by only a few participants. However, in those instances, we clearly stated the number/ proportion of participants adducing such reasons. | Line 278 - 287 |
| 10 | “Economic empowerment schemes” theme: again, this seems only based on one healthcare person’s opinion. If no-one else mentioned this, this should be highlighted. Also comment on whether there appeared to be uptake. | As stated above, we decided to bring out some important concepts which are known from literature to be important barriers or opportunities, even when they were referenced by only a few participants. However, in those instances, we clearly stated the number/ proportion of participants adducing such reasons. | Lines 373 - 381 |
|  | DISCUSSION/CONCLUSION |  |  |
| 11 | The discussion needs to be much tighter. Everything is there, but it is repetitive and too long. I suggest cutting it down significantly and structuring as follows:   - Succinctly summarising the results (2-5 sentences). You could briefly place them in the context of Nigeria in this 1^st^ paragraph, for example highlight that national guidelines recommend routine screen but it is rarely done. - Highlight aspects that are similar/different to other studies – you do not need to go through each result and compare. Rather pick certain ones, ones that are most interesting/relevant/have lots of evidence or don’t. (2 paragraphs) - Explain what this study adds to the literature/evidence (1 paragraph). | Thank you for this recommendation. The discussion section has been modified and shortened accordingly. | Pages 20 to 24 |
| 12 | Conclusion: this can also be shortened. Don’t repeat the results again in the conclusion. The first and last sentence are most relevant to the conclusion. | Thank you for this observation. The conclusion section has been modified and shortened as recommended. | Pages 26 and 27, lines 529 to 539 |

**REVIEWER 2’s COMMENTS**

| **S/No** | **Comments** | **Response/corrections done** | **Reference (page and line)** |
| --- | --- | --- | --- |
| 1 | Line 46 and throughout - please try to avoid use of ‘infections’ (see eg https://www.youtube.com/watch?v=tbx2tbFYSCU) by rearranging the sentence. Language can be very stigmatising for people with HIV. It can increase and perpetuate mental health issues. The word ‘infections’ is unnecessary here. eg ‘1.3 million more people acquired HIV globally in 2024…’ | Thank you for this observation.  This change has been implemented, and the sentence now reads: *…and 1.3 million more people acquired HIV globally in 2024…* | Page 3, line 48 |
| 2 | Line 47 *and throughout* - please avoid using letters to describe people (see above). Instead please spell these out. | This change has been implemented throughout the manuscript. PLWH now replaced with *People living with HIV.* |  |
| 3 | Line 51 and throughout - please try to avoid militaristic language to describe a health condition. | This change has been implemented, and the sentence now reads: *“Over the last two decades, significant gains have been recorded in the global effort to end the HIV pandemic.”* | Page 3, line 53 |
| 4 | Line 62 - please avoid this abbreviation also. How about spelling it all out the first time then say ‘hence described here as caregivers’? | This change has been implemented as recommended. | Page 3, line 64 |
| 5 | Line 65 - I am curious to know why there is no further discussion of trauma or PTSD throughout the paper. Since an HIV diagnosis, especially for women in the context of perinatal health, can often start or increase violence against them and affect their mental health, it seems strange that this is not mentioned any further. | Thank you for this important observation. The close link between trauma, HIV and the resultant post-traumatic stress disorder is widely recognized in published literature. However, this angle was not thoroughly explored in the present study, as it was not part of the study objectives. While we acknowledge that this may be an important limitation of this study, the study participants may not have perceived it as a barrier to accessing mental healthcare services in the study setting. This may explain the reason why it did not emerge as a theme in the present study. |  |
| 6 | Line 67 - So what is going on in Nigeria for other people living with HIV? It would be good to clarify how services differ... | In Nigeria, efforts to improve mental healthcare access for people living with HIV have largely focused on key populations such as adolescents and young people because of emerging mental health challenges that they face as they transition from childhood to adolescence and young adulthood and also due to the high tendency to disengage from HIV care. Mental healthcare services for other key populations, such as men who have sex with men, female sex workers and people who inject drugs, as well as the general population of people living with HIV, are scanty. Little or no attention has been given to women caregivers living with HIV, despite the high prevalence of common mental health disorders among women living with HIV and the peculiar challenges they face in accessing the needed mental healthcare. |  |
| 7 | Line 109 - Were people openly living with HIV involved in the study design, implementation, analysis or write-up at all? If not, why not. See eg WHO 2017, section 6, especially 6.2.1. NB mental health forms an integral part of SRHR. https://iris.who.int/server/api/core/bitstreams/611f9d21-8170-495c-89b2-903886a83aee/content | Thank you for sharing the additional resource material. Yes, three of the counsellors who supported the implementation of this study were living with HIV, though not openly. This helped provide nuanced contexts to the issues elicited from the study participants. However, this information was not provided in the manuscript due to confidentiality purposes and to avoid stigma. |  |
| 8 | Line 122 - I am curious to know why you used this narrow definition of caregiving? Why did you decide, for instance, not to include women living with HIV who are providing care to other family members, including their (sick) partners? See for example, a report from COWHLA in Malawi, which describes the mental health issues faced by women living with HIV who were caring for sick husbands who were in denial about their own HIV status and were refusing to let the women attend clinic to access tx themselves; or who were just abandoned them (https://cowlha.org/cowlhas-impact-through-the-stepping-stones-program-challenging-gender-norms-and-achieving-srhr-for-women-through-the-hiv-response/). | Thank you for this observation.  This has been corrected to reflect accurately the definition of caregivers included in the study which now reads: *A caregiver was defined as a participant who provides unpaid physical support, such as helping in activities of daily living, shopping, food preparation, helping in administering medication, overseeing medical appointments, and providing financial and emotional support to the child/children or other family members.* | Page 8, lines 148 to 151 |
| 9 | Line 128 - what was the gender and age breakdown of these key informants? And were any of them living openly with HIV? | Table 2 in the results section has been updated to provide a breakdown of the gender and age of the key informants. Three of the key informants (counsellors) who supported the implementation of this study were living with HIV, though not openly. | Table 2, Pages 12, lines 227 to 228 |
| 10 | Line 137 - were any of the researchers living openly with HIV? If not, why not? (see comment above about WHO Guideline 2017). | None of the researchers was known to be living with HIV openly at the time of conducting this study. However, one of the research assistants who supported the conduct of the FGDs was living with HIV but not openly. This information was not provided in the manuscript due to confidentiality purposes and to avoid stigma. |  |
| 11 | Line 141 - The experts in their own lived experiences are surely the women living with HIV themselves? Were they consulted? If not, please explain why not? | Involving the women caregivers living with HIV in the design, implementation and analysis of the current research would have added valuable, nuanced insights to the findings of this study. We acknowledge this as an important limitation of this study. The limitations section has been modified to incorporate this. | Pages 26, lines 511 to 528 |
| 12 | Line 151 - What were the education levels reached of all those interviewed? Was English a barrier to anyone taking part? | All key informant interview participants had a tertiary level of education as they were doctors, nurses and counsellors who had a Bachelor's degree in psychology. Information on the level of education of the FGD participants was not obtained in the present study. However, English was not a barrier to anyone taking part in the FGDs. |  |
| 13 | Line 199 - FGD participants - please provide education level (and, if relevant, level of English). Would also be good to know if they had partners at home or not. | Thank you for the suggestion. The study did not obtain data on the FGD participants’ level of education. While all FGD participants were caregivers to at least one child or other family members, the study, however, did not obtain data on whether they were currently living with their partners or spouses. |  |
| 14 | Line 199 - KII participants - please provide gender and age breakdown by job title/level. And if any are openly living with HIV. | Table 2 in the results section has been updated to provide a breakdown of the gender and age of the key informants. Three of the key informants (counsellors) who supported the implementation of this study were living with HIV, though not openly. | Table 2, Page 12, lines 227 and 228 |
| 15 | Line 207 - ‘Ignorance’ is a loaded word - suggest instead 'lack of awareness' or 'limited awareness’. | Thank you for this observation.  This change has been implemented, and the sentence now reads: *Lack of awareness among people living with HIV…* | Page 15, line 278 |
| 16 | Line 207 - ‘low mental health literacy’ - I suggest instead ‘limited mental health literacy’. | Thank you for this observation.  This change has been implemented, and the sentence now reads: *Limited mental health literacy among people living with HIV…* | Page 15, lines 278 to 279 |
| 17 | Line 214. I suggest ‘or’ might be a clearer word to use here, rather than ‘and’. | This change has been implemented, and the sentence now reads: *Many of the healthcare workers reported that mental healthcare services for people living with HIV were either not available or integrated with ART services at the clinic…* | Pages 13 and 14, lines 241 to 243 |
| 18 | Line 215. I suggest a semi-colon after the word ‘clinic’ here, instead of a comma, for clarity. | This change has been implemented as recommended. | Page 14, line 243 |
| 19 | Line 248 - as above. I suggest ‘Lack of awareness and limited mental health literacy’ as more appropriate words, to avoid stigma. | Thank you for this observation.  This change has been implemented, and the phrase now reads: *Lack of awareness and limited mental health literacy* | Page 15, lines 278 to 279 |
| 20 | Line 310-311 - some have described themselves as civil or public servants? | While the FGD participants comprised a mix of women caregivers living with HIV from diverse occupational settings, a major barrier identified by many of them, including the civil or public servants, was the high cost of transportation to the clinic. At the time of conducting this study, the Government of Nigeria had recently removed subsidies on petrol and electricity. This resulted in high fuel costs and inflation. The high transportation cost is a part of a general rise in the cost of living in all Nigerian cities as a result of these government policies. |  |
| 21 | You may wish to look at and refer to the following articles also, to enhance this section. These have all be created by and/or with women living with HIV: eg Orza et al 2015 b. https://onlinelibrary.wiley.com/doi/full/10.7448/IAS.18.6.20289; Orza et al 2015 a. https://onlinelibrary.wiley.com/doi/full/10.7448/IAS.18.6.20285; Orza et al 2017. https://www.hhrjournal.org/2017/12/04/in-womens-eyes-key-barriers-to-womens-access-to-hiv-treatment-and-a-rights-based-approach-to-their-sustained-well-being/#_edn27; Dunaway et al 2022. https://journals.sagepub.com/doi/full/10.1177/17455057221080361#table2-17455057221080361; National Standards for Peer Support for HIV UK 2022. Beres et al 2017. https://positivelyuk.org/wp-content/uploads/2022/06/national_standards_final_web.pdf; https://www.tandfonline.com/doi/full/10.1080/09540121.2017.1317324 | Thank you for this recommendation. Some of the additional resources shared have helped enhance this discussion section. | Page 25, lines 495 to 497 |
| 22 | Line 358 ‘ignorance/low’ - see comments above. | Thank you for this observation.  This change has been implemented, and the sentence now reads: …*Lack of awareness/limited mental health literacy...* | Page 20, line 394 |
| 23 | Line 358 - Was there no mention of VAWG?? I'm really surprised at this - if there wasn’t, then it would seem that working more closely with caregivers as co-researchers might have brought this out... | You are right that VAWG commonly affect women living with HIV; however, the respondents may not have perceived it as a barrier to their accessing mental healthcare services, which was the focus of this research, explaining why it did not come out in their responses. |  |
| 24 | Line 382. ‘Abuse’ is an inappropriate word here. How about ‘harmful substance use' instead? This fits in with a focus on ‘harm reduction’ to support people living with HIV and others. | This change has been implemented, and the sentence now reads: *This has implications for integrating mental health care with routine ART services by HCWs, which has been shown to be effective in achieving viral suppression and reducing depression and other mental health disorders, such as harmful substance use among people living with HIV* | Page 21, line 417 |
| 25 | Line 389 onwards. Again, I am surprised that there was no mention of VAWG when it is so widespread among women living with HIV. | As already explained above, the respondents may not have perceived VAWG as a barrier to their accessing mental healthcare services, which was the research focus, and this could be the reason why it did not reflect in their responses. |  |
| 26 | Line 412 - please change wording again. | This change has been implemented, and the sentence now reads: …*Lack of awareness/limited mental health literacy...* | Page 23, lines 451 to 452 |
| 27 | Line 453 - please see UK National Peer Support standards, ref. above. | Thank you for this recommendation. The resource material shared has helped enhance this discussion section with relevant evidence. | Pages 25, lines 495 to 497 |
| 28 | Line 458 - I suggest there is also the need to talk about the knock-on consequences of mental health of caregivers to the children in their care also - certainly the caregivers have their own intrinsic rights to support - and it’s great that you focus on this here. But there are also instrumental issues here, in terms of adverse childhood experiences and the long-term health of the children… see for example https://journals.sagepub.com/doi/full/10.1177/17455057221080361#table2-17455057221080361 | Thank you for this suggestion. This section has been modified to incorporate this. | Page 22, lines 436 to 438 |
| 29 | Line 458 - I suggest that you also identify the other limitations of the study - eg no mention of VAWG (which is very strange); seeming lack of inclusion of caregivers in study design, implementation or analysis? You could also turn these into suggested points for inclusion in further study… | Thank you for this suggestion. The limitations section has been modified to incorporate this. We acknowledge that co-creating future research in this area with women caregivers living with HIV, especially involving them during the study design, and implementation, will add valuable, nuanced insights. This may help explore in more detail the critical role VAWG at the family, community and health facility levels could play in hindering mental healthcare access for women caregivers living with HIV. | Page 26, lines 521 to 528 |
| 30 | Line 485 - ‘ignorance and low…’ language again. And ‘WCLIHV’… | Thank you for this observation. The conclusion section has been modified to address this. | Page 27, lines 530 to 539 |
