# Supplementary material for: Exploring barriers to and opportunities for improving mental healthcare access for women caregivers living with HIV in Nigeria: A qualitative study
Source: PLOS Glob Public Health. 2026 Aug 14;6(8):e0005834. doi: 10.1371/journal.pgph.0005834 (PMC13475909; doi:10.1371/journal.pgph.0005834)
Supplement: Supplementary file 7 [file pgph.0005834.s005.docx]

This is an interesting paper. The research is important and relevant.

I do have some queries, suggestions and questions. By addressing them I believe the paper will be considerably strengthened. Specifically:

General/Introduction:

1. Check the grammar and writing of the whole article. Generally, the writing is good but there room for improvement. For example, in the abstract (p.2, line 30) the sentence should start “The study….” Rather than just “Study….” In the introduction on page 4 (lines 81-85) the sentence starting “Recognizing the rising need to integrate mental health…….” is extremely long. It should be shortened or divided into 2-3 sentences.
2. Introduction: page 4, lines 72-76 discusses “high income countries”. Are these factors not relevant to low resource contexts? You need to link these findings to the context of this paper; for example say if/why they are relevant to low-resource settings, or replace the sentence with more relevant findings. Alternatively, you could remove the sentence altogether as it feels a bit out of place.

Methods:

3. I suggest having a paragraph/section on Levesque’s framework (and the table) under study design – it is currently detailed in the section on analysis, so it can be removed from there. The framework informs the whole study so would be better under study design. It can be referred to briefly in the analysis.
4. Were FGDs conducted in English? If so state they are (it only says interviews were conducted in English – line 151, page 7). If not, say the language/s and about the process of translation.
5. Analysis – there needs to be more details on the process of analysis. For example, how were the data coded? Was software used to help organise and code? I recommend outlining briefly the steps taken for analysis.
6. Reflexivity is mentioned (which is good), by saying researchers “tried to be aware and transparent”. However, no details are given on how this is done, steps taken to mitigate bias or examine influence. Examples could be regular discussions, debriefs, reflexive notes etc. Please add some details.

Results:

7. “Ignorance and low mental health literacy” theme: please consider dropping the term “ignorance” from the theme title as it has quite negative connotations. You could simply have “Low mental health literacy” or “Poor knowledge and low mental health literacy.
8. “Ignorance and low mental health literacy” theme: this theme appears to be based only on one participant’s opinion (a nurse). Normally for a theme to emerge there should be some level of consensus, or it to be referred to several times. Was it identified by others? Did others comment on this as a barrier? It is important to say if so – and also if not.
9. “Economic empowerment schemes” theme: again, this seems only based on one healthcare person’s opinion. If no-one else mentioned this, this should be highlighted. Also comment on whether there appeared to be uptake.

Discussion/Conclusion:

10. The discussion needs to be much tighter. Everything is there, but it is repetitive and too long. I suggest cutting it down significantly and structuring as follows:

- Succinctly summarising the results (2-5 sentences). You could briefly place them in the context of Nigeria in this 1^st^ paragraph, for example highlight that national guidelines recommend routine screen but it is rarely done.
- Highlight aspects that are similar/different to other studies – you do not need to go through each result and compare. Rather pick certain ones, ones that are most interesting/relevant/have lots of evidence or don’t. (2 paragraphs)
- Explain what this study adds to the literature/evidence (1 paragraph).

11. Conclusion: this can also be shortened. Don’t repeat the results again in the conclusion. The first and last sentence are most relevant to the conclusion.
