# Supplementary material for: Exploring barriers to and opportunities for improving mental healthcare access for women caregivers living with HIV in Nigeria: A qualitative study
Source: PLOS Glob Public Health. 2026 Aug 14;6(8):e0005834. doi: 10.1371/journal.pgph.0005834 (PMC13475909; doi:10.1371/journal.pgph.0005834)
Supplement: Supplementary file 8 [file pgph.0005834.s008.docx]

**Peer Review Report (Revision 2)**

**PLOS Global Public Health**

Name of Reviewer: Mohammed Abu Sayeed, Associate Professor, Department of Pharmacy, International Islamic University Chittagong, Chittagong, Bangladesh

Manuscript title: *Exploring barriers to and opportunities for improving mental healthcare access for women caregivers living with HIV in Nigeria: A qualitative study*
Manuscript number: PGPH-D-25-03123_R2

**Overall Assessment**

**Recommendation:** **Minor Revision / Accept after minor editorial corrections**

The R2 manuscript is considerably stronger than the previous version in terms of methodological rigor, reporting transparency, reflexivity, and discussion. Most substantive reviewer concerns appear to have been adequately addressed.

**Reviewer Comments vs R2 Evaluation**

| **Major Reviewer Concern** | **Status in R2** | **Evaluation** |
| --- | --- | --- |
| Clarify conceptual framework | Levesque framework fully described and contextualized with a summary table and figure. | **Fully addressed** |
| Improve methodology details | Sampling, recruitment, interview procedures, and participant selection clearly explained. | **Fully addressed** |
| Explain data saturation | Saturation process explicitly described, including two-phase confirmation approach. | **Fully addressed** |
| Reflexivity and researcher role | Reflexivity statement added with description of reflexive notes and collaborative interpretation. | **Fully addressed** |
| COREQ reporting | Authors explicitly state adherence to COREQ guidelines. | **Fully addressed** |
| Expand Discussion with literature | Discussion substantially expanded with numerous recent citations and comparisons with prior studies. | **Fully addressed** |
| Add Strengths and Limitations | Dedicated section added. | **Fully addressed** |
| Improve implications and recommendations | Practical recommendations including digital referral systems included. | **Fully addressed** |

**Remaining Minor Issues**

**1. Inconsistency in number of themes**

The Abstract states:

"Seven themes..."

However, Table 4 presents:

- six barrier themes
- at line 37, 38, it is mentioned 8 themes
- two opportunity themes

This indeed totals eight themes, but the wording throughout the manuscript occasionally refers to "seven themes". Please ensure consistency throughout all sections.

**2. Minor grammatical polishing still needed**

A few sentences require language editing.

Examples:

- "with resultant increase in mental health needs" → should read **"resulting in increased mental health needs"**.
- "Women and girls bear a substantial burden of the HIV pandemic accounting..." → consider:

**"Women and girls bear a substantial burden of the HIV pandemic, accounting for..."**

- "This means that HIV is no longer a fatal and terminal disease..." could be simplified for readability.

**3. Data analysis description**

The manuscript simultaneously mentions:

- thematic analysis
- content analysis
- directed content analysis

The authors should explicitly state that they used:

"Directed qualitative content analysis informed by Levesque's framework"

to avoid methodological ambiguity.

**4. Participant involvement statement**

The limitation section mentions:

"some who took part in conducting the research are living with HIV..."

This statement is important but somewhat unclear. The authors should clarify whether these individuals were:

- co-researchers,
- peer researchers,
- patient representatives, or
- research assistants.

**Final Recommendation to Editor**

The authors have adequately addressed the substantive concerns raised during previous rounds of review. The manuscript has been substantially strengthened, particularly regarding methodological transparency, analytical rigor, reflexivity, and contextualization of findings. Only minor editorial revisions relating to language clarity and methodological consistency are recommended before acceptance.

**Probability of acceptance:**

**High (>90%) following minor editorial revision.**
