# Supplementary material for: Exploring barriers to and opportunities for improving mental healthcare access for women caregivers living with HIV in Nigeria: A qualitative study
Source: PLOS Glob Public Health. 2026 Aug 14;6(8):e0005834. doi: 10.1371/journal.pgph.0005834 (PMC13475909; doi:10.1371/journal.pgph.0005834)
Supplement: Supplementary file 9 [file pgph.0005834.s009.docx]

Ugochukwu Uzoechina Nwokoro

Department of Community Medicine

Federal Teaching Hospital, Owerri, Nigeria

[ugo_nwokoro@yahoo.com](mailto:ugo_nwokoro@yahoo.com)

26^th^ June, 2026

Dr. Katherine Demi Kokkinias

Staff Editor

PLOS Global Public Health

**RE: PGPH-D-25-03123R1 - Exploring barriers to and opportunities for improving mental healthcare access for women caregivers living with HIV in Nigeria: A qualitative study**

Dear Dr. Kokkinias,

Thank you for the opportunity to submit a revised version of our manuscript, “**Exploring barriers to and opportunities for improving** **mental healthcare access for women caregivers living with HIV in Nigeria: A qualitative study,”** for consideration for publication in PLOS Global Public Health. We sincerely appreciate the constructive feedback provided by you and all the reviewers. Their insightful comments have allowed us to substantially improve the quality, clarity, and impact of our study.

We have carefully addressed all of the reviewers' comments and have revised the manuscript accordingly. All punctuation, typographical and grammatical errors in the manuscript have been corrected. In the point-by-point response provided below, we detail the changes made to the manuscript. All modifications in the revised munuscript have been highlighted using track changes in a separate file labelled 'Revised Manuscript with Track Changes' uploaded in the online manuscript submission system. An unmarked version of the revised manuscript, without tracked changes, labelled ‘Manuscript’, has also been uploaded to the online manuscript submission system along with this rebuttal, as recommended.

Please find below a point-by-point response to each reviewer's comments.

We hope that the revisions and our accompanying responses thoroughly address the concerns raised and that our manuscript is now suitable for publication in PLOS Global Public Health. We look forward to hearing from you regarding your decision.

Sincerely,

Ugochukwu Uzoechina Nwokoro.

**REVIEWER 2’s COMMENTS**

| **S/No** | **Comments** | **Response/corrections done** | **Reference (page and line)** |
| --- | --- | --- | --- |
|  | ABSTRACT |  |  |
| 1 | line 21. Thank you for removing the word 'infection' in one place. Please check also here and the rest of the article and remove 'infection' everywhere unless it is in the references. It is unnecessary and can further stigma. | We agree with this observation.  This change has been implemented, and the word ‘infection’ has been removed throughout the manuscript as recommended. |  |
| 2 | Line 26 and throughout the article. Thank you for changing this throughout. However I suggest that for better flow, once you have described the group, you could just call them 'these caregivers' in most places, just using the full phrase again, to remind the reader who they are, when you mention them for the first time in each new section of the article. | We appreciate this recommendation. This change has been implemented in most places throughout the manuscript, as recommended. |  |
|  | INTRODUCTION | | |
| 3 | line 69. Thank you for now mentioning the lack of mention of VAWG in the limitations section. I think you may very well be right that they don't see it as a barrier to accessing mental health care. Unfortunately, VAWG can be so normalised (especially if it's in the form of coercive control or marital rape, rather than physical violence), that it may not be recognised by those on the receiving end as a stressor. It can also be a(nother) great source of shame and women may also feel that the VAWG is justified because they have HIV - and they may also fear that their children may be taken away from them. In either situation, it can of great help to the individual concerned if the possibility of VAWG (and, in this case, its links to mental health) is raised by the practitioner, to enable the woman to recognise that VAWG is never justified - and that it is absolutely ok to talk about it - and have it stopped, and its effects recognised and mitigated. I suggest you might consider this in future research. | Thank you for highlighting this important point about VAWG and for bringing these contexts and valid perspectives to the fore. We would be considering this in our future research efforts. |  |
| 4 | Line 67 (now 71). Thank you for the helpful information you shared about mental health provision for others with HIV in Nigeria. This is very informative as background context for the reader to know. Could you add it to the article please. | Thank you for this recommendation. This has been included as additional background context for readers in the introduction section. | Page 5, lines 96 to 104 |
|  | RESULTS |  |  |
| 5 | line 246. Table 2. Part 1 - FGD participants. Could this be presented as a box, with 2 columns and 2 rows (ie 4 cells for data), in order to see the no. of participants who are eg 18-24 who have been on ART for <5 years, etc? This would provide further clarity to the reader. | Thank you for this recommendation. The characteristics of the FGD participants have been presented as recommended and now show clearly the number of participants in each age category, disaggregated by duration on ART care. | Page 13, Table 2 |
| 7 | Table 2 part 2. KII participants. Thank you for the disaggregation by gender. Could this be presented as a box too, so that we can see the breakdown by age and years experiencing care (x and y axes), with each of these 5 boxes subdivided by gender (so 12 data boxes in total). Again this would be helpful to the reader. | Thank you for this recommendation. The characteristics of the KII participants have been presented as recommended and now show clearly the number of participants in each age category and years of experience providing ART care, disaggregated by gender. | Page 13, Table 3 |
|  | DISCUSSION | | |
| 8 | line 463. ACEs affect boys and men as well as girls and women. Maybe better to state this here. | Thank you for this suggestion. This has been modified to indicate that the barrier posed by stigma and discrimination to accessing mental healthcare is heightened for all individuals with a background of ACEs. However, this is particularly so for women and girls living with HIV who have experienced ACEs. | Pages 23 and 24, lines 462 to 465 |
| 9 | line 550. About limited inclusion. Thank you for adding this here. I would suggest that you also add something here like: 'Lastly, *although some who took part in conducting the research are living with HIV, this has not been documented here, owing to ongoing stigma and discrimination that can arise. Despite their involvement,* ....the limited inclusion... ' | Thank you for this recommendation.  This change has been implemented. | Page 27, lines 551 to 553 |
| 10 | Line 552. about the critical role: Suggest you amend wording to eg "... to explore certain key areas facing participants. For example, fear of.... could play a critical role... | Thank you for this recommendation.  This change has been implemented. | Page 28, lines 555 to 557 |

**REVIEWER 3’s COMMENTS**

| **S/No** | **Comments** | **Response/corrections done** | **Reference (page and line)** |
| --- | --- | --- | --- |
| 1 | No major concerns were identified regarding research ethics and publication ethics.  One minor comment and issues related to clarity and readability, including repetition of key terms and long sentences that could be simplified. | Thank you for pointing this out. These changes have been implemented throughout the manuscript as recommended. Sentences have been shortened to improve clarity and readability. |  |

**REVIEWER 4’s COMMENTS**

| **S/No** | **Comments** | **Response/corrections done** | **Reference (page and line)** |
| --- | --- | --- | --- |
|  | ABSTRACT | | |
| 1 | In general, the guideline of the journal is not described. This is seen on the abstract since it lacks sub sections. It also have consistencies for example it presented accommodation as a six component of access but missed in the others sections. Again, sven themes in the discussion and five themes were presented in the methods section and why? | Thank you for this observation. We respectfully clarify that the abstract is written according to the PLOS Global Public Health journal guidelines, which require that abstracts be conceptually structured into background, methods, results and conclusion, but without distinctly stating the sub-sections.  Table 4 (previously Table 3) reports the identification of existing support groups and economic empowerment schemes by study participants as opportunities for improving access to mental health care, and these themes were categorized as belonging to the availability and accommodation dimension of the Levesque and colleagues’ framework because they facilitate the ability to seek mental health care services among the study population.  Eight broad themes were identified in the study and elaborated in the discussion section of the manuscript. This has been corrected to ensure consistency across the manuscript. Of the eight themes, **six** were barriers (1. unavailability of mental healthcare services integrated with HIV services, 2. lack of training for healthcare workers on mental healthcare, 3. lack of awareness/limited mental health literacy, 4. stigmatization, 5. long clinic waiting time and 6. transportation costs/productivity losses) and **two** were opportunities (1. existing support groups and 2. economic empowerment schemes).  The themes align with the five dimensions of health care access based on the Levesques and colleagues’ framework for health care access. The five dimensions and how they relate to mental healthcare access are summarized in the methods section (Table 1) and not the themes identified in the present study. | Pages 2 and 3, lines 21 to 48  Page 14, Table 4  Page 21, lines 413 to 416 and Pages 22 to 26, lines 417 to 528  Page 7 and 8, Table 1 |
| 2 | Specifically, the abstract is weak. This was evidenced by the weak knowledge gap and definition of HIV. The type of the study design is also not mentioned. How many FGDs were conducted? The result needs more brief presentation and the conclusion is not focused. | Thank you for pointing this out. The abstract section has been strengthened by including the research gap, the study design and the number of FGDs conducted. We respectfully clarify that the results subsection of the abstract presents the eight broad themes (six of which are barriers to access and two of which are opportunities for improving access) that were identified by study participants and that these themes cut across the five dimensions of access to healthcare based on the Levesques and colleagues’ framework for access to healthcare.  Based on the findings of our study, the conclusion subsection of the abstract focuses on highlighting that the barriers to access to mental healthcare are multi-level, relating to health system-level and patient-level factors and that efforts to integrate mental healthcare into routine HIV need to address these barriers while leveraging existing opportunities for improving mental healthcare access | Pages 2 and 3, lines 21 to 48 |
|  | INTRODUCTION | | |
| 3 | In the introduction section the number, severity and size as well as distribution of HIV, its complications and the initiatives and the vivid gaps and how to fill the gaps were not presented. For instance, what is the magnitude of HIV in Nigeria? What type of mental disorders and is this mental disorders related to the HIV itself [unexplained neuro-psychiatric disorders] or due to giving care? Some of the sentences lack references, why E.g. line 85-90? The paragraphs are weakly constructed and linked. Who are these vulnerable groups? What are the packages of this mental health services? | Thank you for this observation. We respectfully clarify that while the focus of the present study was not on HIV itself but on identifying barriers to and opportunities for improving mental healthcare access for women caregivers living with HIV, who are a vulnerable group among people living with HIV, we reported the prevalence of HIV when describing the study area and setting in the methodology section. The introduction section highlights the research gap (Page 5, lines 90 to 109), the common mental health disorders reported among the women caregivers living with HIV (Page 4, lines 70 to 74). References 14, 15 and 16 are provided for statements in lines 85 to 90. Women caregivers living with HIV who are the focus of the present study are specifically mentioned to be a vulnerable group among people living with HIV (Page 4, lines 68 to 70). Nigeria’s national guidelines for the treatment of HIV recommend that people living with HIV should receive baseline and ongoing assessments for mental health disorders as part of integrated HIV/mental health care through routine screening for common mental health disorders such as depression and anxiety as part of comprehensive HIV care (Page 5, lines 90 to 93). | Pages 3 to 6, lines 63 to 112 and lines 118 to 122 |
|  | METHODS | | |
| 4 | In the methods section, what is the type of qualitative study design you have employed? Why FGDs? Why KIIs? What other parameters have you used in addition to “…in a secure and convenient location within the study sites” line 169. Were the interviews recorded? Most importantly, the quality and experience of the data collectors and how many data collectors were involved in the FGDs? Moreover, the trustworthiness of the data is NOT PRESENTED. WHY? Psycho-therapy is part of ART and why you focus on mental health and what is their difference? It didnt seem you have used soft-ware for the analysis? | Thank you for this observation. We have clarified this in the manuscript to show that we conducted a qualitative phenomenological study involving the use of semi-structured interviews in the form of focus group discussions (FGDs) and key informant interviews.  The data collection section has been modified to show that, in addition to conducting the interviews in secure, convenient locations, participants’ privacy and research integrity were also ensured and prioritized.  FGDs and KIIs were conducted to obtain information from different stakeholders on their lived experiences with mental healthcare access for women caregivers living with HIV and to ensure data triangulation. Specifically, FGDs were conducted among women caregivers to explore their experiences with barriers to and opportunities for improving access to mental health care while accessing their routine HIV care at the selected health facilities. We also conducted key informant interviews (KIIs) to obtain information from healthcare workers providing clinical care to people living with HIV, to obtain information on their experiences with mental health care availability and access for women caregivers living with HIV at the study sites.  Interviews and FGDs were conducted in English and recorded using a digital recorder with the consent of the study participants.  A total of five experienced data collectors (three physicians and two research assistants (one resident doctor and a clinical counsellor), all of whom work in the study sites supporting the provision of HIV care served as the data collectors and conducted the interviews.  The approaches adopted to ensure the trustworthiness of the data are described in details in the data management and analysis section of the manuscript.  Mental health is the overarching state of one's psychological, emotional, and social well-being, while psychotherapy refers to one of the many medical and clinical treatments used to improve mental health. Our study focused on identifying barriers to accessing mental healthcare (including psychotherapy and other forms of mental healthcare where they may be available) for an important subgroup of people living with HIV and the opportunities for improving access.  We have updated the data management and analysis subsection to clarify that, to ensure analytical depth and researcher immersion, all qualitative data collected during this study were analyzed manually without the use of computer-assisted qualitative data analysis software (CAQDAS). Analysis rigorously followed the inductive thematic analysis framework: data familiarization, generating initial codes, searching for themes, reviewing themes, defining and naming the themes and ensuring trustworthiness. | Page 7, lines 131 to 136  Page 10, lines 180 to 181  Page 9 and 10, lines 156 to 176  Pages 10 and 11, lines 194 to 196  Page 10, line 179 to 181  Pages 11 and 12, lines 203 to 237  Page 11, lines 204 to 208 |
|  | RESULTS | | |
| 8 | In the result section, the whole section needs rewriting despite its coherence. This was manifested by lack of presenting both the negative and positive findings and its detail. For example lack of training of mental health integration with ART and why is that? Similarly, the approachability is for example given less detail presentation. IN addition, the identifier [E.g. age] of the study participants and the selection of appropriate and illustrative quote is doubtable. Some of the findings are questionable. For example, long clinic waiting time if there is no service how do they wait for a longer period of time? Why you fail to present the figure that contain the themes, categories, codes and quotations. On to top of that, if you have you used framework that contain five components why you presented seven components among which two is out of the framework? | Thank you for pointing this out. To provide a bit more context, we respectfully clarify that the results of the study are presented in line with the Levesque and colleagues’ framework for access to healthcare, which guided the analysis of the study transcripts.  Specifically, one of the critical barriers to access to mental healthcare services for women caregivers living with HIV identified by some healthcare workers was the lack of training for healthcare workers already providing routine HIV care to build their capacity to recognize and screen for mental health disorders among people living with HIV while providing routine HIV care in the clinic. This may be because the training for these healthcare workers focuses only on building their capacity to provide HIV care, with little or no attention paid to building their capacity to also screen for and provide mental healthcare to those who need it at the time of accessing their routine HIV care.  Lack of awareness and limited mental health literacy was identified as a theme under the approachability dimension in the study. In this instance, we decided to highlight this concept, known from the literature as a key barrier, even though it was referenced by only a few participants. Furthermore, to ensure transparency, in instances like this, we clearly stated the number/ proportion of participants adducing such reasons.  We did not include personal identifiers such as the age of participants in the quotes provided within the manuscript because they may potentially identify the healthcare workers who participated in the study and compromise confidentiality. We only indicated the participants’ ID numbers and their designations for quotes in the manuscript as including other identifiers may compromise the confidentiality and anonymity of the study participants and promote stigma against the study participants.  Some participants identified that long clinic waiting time experienced by people living with HIV when accessing their routine HIV care discourages them from seeking additional mental healthcare when they need it because that would mean spending additional time in the clinic after receiving routine HIV care to access mental healthcare, where it is provided.  Thank you for the observation regarding not including a figure containing the themes, categories, codes and quotes in the manuscript. We respectfully clarify that because our qualitative transcripts were analyzed manually, we used an iterative, interpretive approach rather than software-based code extraction. Consequently, we considered that a single, comprehensive figure encompassing the extensive codebook, categories, and numerous supporting quotes would lack the visual clarity needed for the manuscript. Instead, to provide a transparent and accessible presentation of our findings, we detailed the development from raw data to thematic constructs directly in the Methods section to illustrate the analytical process clearly. The main areas, themes and sub-themes are supported by embedded participant quotations provided in the Results section. To fully address your comment and enhance readability, we have clarified this in the data management and analysis section, and we have now provided an Appendix (Supplementary File 3) that explicitly outlines the relationship between our key themes, sub-themes, and the foundational codes, as suggested.  The Levesque and colleagues’ framework groups barriers to access to healthcare into five dimensions. The present study identified eight themes which cut across the five dimensions of health care access based on the framework. Six of the themes were barriers to access, while two of the themes posed opportunities for improving mental healthcare access. | Page 7, lines 138 to 147 and Page 8, Table 1  Page 16, lines 297 to 303  Pages 16 and 17, lines 305 to 314  Pages 15 to 21, lines 274 to 406  Pages 17 and 18, lines 326 to 345  Page 11, lines 208 to 211  S3 File: Themes identified, categories, subthemes, codes, descriptions and quotations. |
|  | DISCUSSION | | |
| 10 | Likewise, the discussion is relatively good. It fails to firmly present the main findings. It was poorly organized. This was evidenced by too long paragraphs. Avoid discrimination [line 435] which is out of the study objective. Similarly, it is not well organized. E.g. the opportunities were presented before the barriers. Furthermore, summarize well and present shortly. Explain well, justify it and use appropriate up to date references with important implications. Write the conclusion and the recommendation in consistent with the recommendation. Check some of the references. E.g. in reference Information sheet should be changed to fact sheet. | Thank you for pointing this out. The term discrimination has been removed. The discussion section has been modified with shortening of some paragraphs to improve clarity and readability.  We respectfully clarify that opportunities were presented after the barriers in the discussion section. We highlighted in the conclusion section that our study findings suggest that the barriers to access to mental healthcare are multi-level, relating to health system-level and patient-level factors and that efforts to integrate mental healthcare into routine HIV care need to address these barriers while leveraging existing opportunities for improving mental healthcare access for the caregivers.  The references section has been updated as recommended. | Pages 21 to 26  Pages 21 to 25, lines 408 to 503 (barriers) and Pages 25 to 26, lines 504 to 528 (opportunities)  Page 29, line 583 and Page 32, line 655 |

**REVIEWER 6’s COMMENTS**

| **S/No** | **Comments** | **Response/corrections done** | **Reference (page and line)** |
| --- | --- | --- | --- |
| 1 | The authors should briefly clarify if any saturation point was formally identified during the content analysis process to further justify the sample size | Thank you for this important recommendation.  This has been elaborated further in the data collection, management and analysis sections of the manuscript. | Page 11, lines 196 to 201  Page 12, lines 225 to 226 |
| 2 | The authors mention that Nigerian national guidelines recommend routine mental health screening, yet it is rarely done. The discussion should more aggressively address why the gap exists between policy and practice, specifically regarding the Task-Shifting and Task-Sharing (TSTS) policy in Nigeria. | Thank you for this important recommendation. The discussion section has been updated to address why the gap exists. The section now reads “*The reason for this gap between policy and practice may be that the training for these healthcare workers focuses only on building their capacity to provide HIV care, with little or no attention paid to building their capacity to simultaneously screen for and provide mental healthcare to those who need it while accessing their routine HIV care.*” | Page 23, lines 443 to 446 |
| 3 | In the limitations section, the authors candidly note that Violence Against Women and Girls (VAWG) was not deeply explored due to the study design. Given the known link between HIV, caregiving stress, and domestic violence, the authors should suggest this as a primary avenue for future longitudinal research | Thank you for this recommendation. We recognize that co-creating future research on this with the caregivers themselves will provide the opportunity to deeply explore their lived experiences, including VAWG and how this shapes access to mental healthcare for this important sub-population. This is suggested as an area for future research in the concluding sentence of the limitations section. | Page 28, lines 558 to 560 |
| 4 | The study notes the lack of a "two-way referral system” on page 15, line 264.The authors should provide a specific recommendation on how digital health or "referral trackers" could mitigate this systemic failure. | Thank you for this suggestion. The conclusion section has been modified to include this specific recommendation. The section now reads “*To address systemic failures such as the absence of a functional two-way referral system between ART clinics and specialized mental health clinics, implementing digital health solutions and referral trackers is highly recommended. These tools help bridge communication gaps, track patient linkage to mental health specialists, monitor treatment continuity, and enhance care coordination.*” | Pages 28 and 29, lines 572 to 576 |

**REVIEWER 7’s COMMENTS**

| **S/No** | **Comments** | **Response/corrections done** | **Reference (page and line)** |
| --- | --- | --- | --- |
| 1 | Consider refining some sentences for improved readability and conciseness. Ensure consistency in formatting (e.g., units, abbreviations, referencing style). A brief elaboration on limitations would strengthen the discussion section. | Thank you for these recommendations. Some sentences have been refined and shortened throughout the manuscript to improve clarity and readability. The limitations section has also been further elaborated on. | Pages 21 and 22, lines 411, 422 to 425  Pages 27 and 28, lines 551 to 557 |

**REVIEWER 8’s COMMENTS**

| **S/No** | **Comments** | **Response/corrections done** | **Reference (page and line)** |
| --- | --- | --- | --- |
| 1 | For the fact that the study focuses on women caregivers living with HIV, the use of the expression "people living with HIV" is not inconsistent. It is suggested that "women caregivers living with HIV" for consistency | Thank you for pointing this out. We respectfully clarify that, although our study focuses on women caregivers living with HIV, we used the expression “people living with HIV” in the introduction section to demonstrate that they are the broader population among whom women caregivers living with HIV are an important subset and that they may experience some shared challenges to access to mental healthcare. |  |
| 2 | There was no mention of saturation with the purposive sampling used. There should be a statement that indicated that the collection continued until thematic saturation was reached | Thank you for this observation. The data collection, management and analysis sections have been updated to include a statement on saturation as recommended. | Page 11, lines 196 to 201  Page 12, lines 225 to 226 |
| 3 | Specify in the methodology section what the participants were caring for, may be ageing parents or children (or both) | Thank you for this recommendation. We respectfully clarify that the methodology section specifies that *a caregiver is defined as a participant who provides unpaid physical support, such as helping in activities of daily living, shopping, food preparation, helping in administering medication, overseeing* *medical appointments, and providing financial and emotional support to the child/children or other family members* | Page 9, lines 160 to 163 |
| 4 | Long clinic waiting time was quoted as a barrier, but no mention of the average wait time. If available, add quantitative context. For example, you may present the result as: "Patients reported waiting 2-4 hours on average." | Thank you for this suggestion. We acknowledge that indicating the average waiting time will provide a quantitative context for the long clinic waiting time adduced by the study participants; however, our study did not obtain data on the actual waiting time spent by the participants when accessing care. |  |
