# Supplementary material for: Exploring barriers to and opportunities for improving mental healthcare access for women caregivers living with HIV in Nigeria: A qualitative study
Source: PLOS Glob Public Health. 2026 Aug 14;6(8):e0005834. doi: 10.1371/journal.pgph.0005834 (PMC13475909; doi:10.1371/journal.pgph.0005834)
Supplement: Supplementary file 10 [file pgph.0005834.s010.docx]

Ugochukwu Uzoechina Nwokoro

Department of Community Medicine

Federal Teaching Hospital, Owerri, Nigeria

[ugo_nwokoro@yahoo.com](mailto:ugo_nwokoro@yahoo.com)

24^th^ July, 2026

Dr. Andrés F. Henao-Martínez

Academic Editor

PLOS Global Public Health

**RE: PGPH-D-25-03123R2 - Exploring barriers to and opportunities for improving mental healthcare access for women caregivers living with HIV in Nigeria: A qualitative study**

Dear Dr. Henao-Martínez,

Thank you for your time and the opportunity to submit a revised version of our manuscript, “**Exploring barriers to and opportunities for improving** **mental healthcare access for women caregivers living with HIV in Nigeria: A qualitative study,”** for consideration for publication in PLOS Global Public Health. We also wish to express our profound gratitude to the reviewers for their careful reading of our work and for providing insightful and constructive feedback.

We have carefully considered all the comments and suggestions and have revised the manuscript accordingly to improve its clarity, rigor, and impact. All punctuation, typographical and grammatical errors in the manuscript have been corrected. In the point-by-point response provided below, we detail the changes made to the manuscript. All modifications in the revised manuscript have been highlighted using track changes in a separate file labelled 'Revised Manuscript with Track Changes' uploaded in the online manuscript submission system. An unmarked version of the revised manuscript, without tracked changes, labelled ‘Manuscript’, has also been uploaded to the online manuscript submission system along with this rebuttal, as recommended.

Please find below a point-by-point response to each reviewer's comments.

We believe these revisions have significantly strengthened the quality of our manuscript and hope that it is now suitable for publication in PLOS Global Public Health.

Thank you again for your time and guidance during this peer-review process. We look forward to hearing from you regarding your decision.

Sincerely,

Ugochukwu Uzoechina Nwokoro.

**REVIEWER 4’s COMMENTS**

| **S/No** | **Comments** | **Response/corrections done** | **Reference (page and line)** |
| --- | --- | --- | --- |
|  | ABSTRACT | | |
| 1 | We thank the authors for addressing the comments given on the previous versions. The abstract didn’t follow the guideline and format of the journal.For example the sub sections are missing and the background is wide. | Thank you for this observation. We respectfully clarify that the abstract follows the PLOS Global Public Health guidelines, which require that abstracts be conceptually structured into background, methods, results and conclusion, but without distinctly stating the sub-sections (kindly see PLOS Global Public Health abstract guidelines: <https://journals.plos.org/globalpublichealth/s/submission-guidelines#loc-abstract>)  We appreciate the constructive feedback regarding the length of the abstract’s background section.  We completely agree that a concise background is vital for readability. However, we respectfully clarify that the background section of the abstract was expanded in the previous version of the manuscript as an earlier reviewers’ recommendation advised us to explicitly include the research gap. We believe that the research gap is critical for establishing the manuscript’s foundational purpose.  To satisfy both requirements while adhering to the abstract’s word limit, we have retained the research gap as previously suggested, but have shortened the background information for conciseness. | Pages 2 and 3, lines 21 to 47  Page 2, lines 21 to 27 |
|  | INTRODUCTION | | |
| 2 | The introduction fails to completely present the scientific contents of it.The efforts made by the government and its partnersa in the continum of HIV care services and its gap were not well described. Try to summerize it well. Try to refrence it accordingly and entertain the gap with appropriate problem statement. | Thank you for this constructive feedback. We appreciate the opportunity to respectfully clarify that the introduction section did outline the scientific contents and gaps regarding mental healthcare access for women caregivers living with HIV.  The introduction section specifically outlines the efforts made by the government and partners in the continuum of HIV care including improving mental healthcare access and the gaps that persist despite these efforts.  These efforts include ensuring national guidelines for treatment and management of HIV recommend routine screening for common mental health disorders among people living with HIV. However, this routine screening is not being done by health care workers. Additional gaps relate to these efforts focusing largely on adolescents and young people living with HIV, neglecting other key populations such as women caregivers living with HIV, who are the focus of this study. | Pages 4 and 5, lines 64 to 86  Pages 5 and 6, lines 86 to 105 |
|  | METHODS | | |
| 3 | The methods section is not comphrensive. E.g. Where is the type of the data collection tool and where the data was collected where is the trustworthiness of the collected data.When and how was the analysis initiated. | Thank you for pointing these out.  We respectfully clarify that the data collection section of the manuscript describes in details the type of data collection tools used for the study (FGD guide and KII guide). These tools have been provided as supplementary files (S1 File) and (S2 File) together with the manuscript.  Additionally, we respectfully clarify that data was collected at the study sites (ART clinics of the selected health facilities) on clinic attendance days through FGDs and KIIs.  Thank you for the insightful comment on trustworthiness of our study’s data. We fully agree that establishing trustworthiness is paramount. In qualitative research, trustworthiness is traditionally evaluated through the lenses of credibility, transferability, dependability, and confirmability.  To ensure our findings are robust and transparent, we integrated specific measures across the four pillars of trustworthiness. The data management and analysis section of the manuscript has been modified to explicitly outline these measures.  All approaches adopted to ensure the trustworthiness of our data are outlined in details in the data management and analysis section of the manuscript.  Thank you for the valid observation regarding when and how data analysis was initiated. We respectfully clarify that in our study, data collection and analysis occurred concurrently rather than as strictly sequential processes.  Though not explicitly stated in the manuscript, formal analysis was initiated in late August 2024, immediately following the completion of the initial phase of 10 KIIs and 3 FGDs.  To ensure a clear understanding by readers of the step-by-step processes involved in the data analysis, we outlined a detailed description of the analysis in the data management and analysis section of the manuscript. | Page 10, lines 180 to 192  Page 10, lines 177 to 180  Page 11, lines 207 to 213  Pages 11 to 13, lines 205 to 241  Page 11, lines 195 to 200  Page 11, lines 195 to 200  Pages 11 to 13, lines 201 to 241 |
|  | RESULTS | | |
| 4 | The result is good and enrich to be more accurate,brief and clear. For instance the quality of the quotations are poor.Try to be in line with the framework and the opportunities are few why? | Thank you for the valuable feedback on the quotations selected, their alignment with our analytical framework and for the opportunity to respectfully clarify our qualitative reporting approach. We recognize that in qualitative research, the "quality" of a quotation is often defined by its richness, depth, and direct alignment with the study's themes rather than its grammatical perfection or eloquence.  We respectfully clarify that the quotations selected in our manuscript were specifically chosen because they best represent the lived experiences, diverse perspectives, and core narratives of our participants.  We provide additional context for these quotes in the results section where needed to help the reader better appreciate their significance and alignment with our analytical framework (Levesques and colleagues’ framework on health care access).  Thank you for the valid observation regarding the opportunities identified in the study. We respectfully clarify that we recognize that in qualitative research, particularly when employing reflexive thematic analysis as outlined in our study, the rigor and value of the findings lie in the depth and complexity of the themes, rather than the quantity of themes generated. We deliberately focused on identifying rich and comprehensive themes that deeply explain the core patterns across our dataset and directly answer our central research question. Each of the two themes identified as opportunities for improving mental healthcare access is strongly supported by our data and reflects a distinct conceptual finding. | Pages 15 to 21, lines 268 to 409 |
|  | DISCUSSION | | |
| 5 | The discussion should be accurately referenced, explained and argued. Most importantly, drawing of implications and references as well as smooth flow of the ideas is missed. The strength and limitations should be part of the study itself not from else others. Regards, | Thank you for this valuable feedback. We appreciate the opportunity to respectfully clarify that in-text citations are provided in the discussion section of the manuscript and a full reference list also provided according to PLOS Global Public Health referencing guidelines. We organized the discussion section in line with themes identified according to the Levesque and colleagues’ framework adopted by the study discussing our study findings alongside their implications for integrating mental healthcare with routine HIV care.  Thank you for the observation regarding the strength and limitations of our study. We respectfully clarify that the strengths and limitations described in the manuscript are intended to serve as a precise, transparent account of the empirical realities and methodological constraints encountered during our study; furthermore, these limitations have helped identify critical areas and directions for future research. | Pages 21 to 27, lines 410 to 531  Pages 27 and 28, lines 535 to 563 |

**REVIEWER 7’s COMMENTS**

| **S/No** | **Comments** | **Response/corrections done** | **Reference (page and line)** |
| --- | --- | --- | --- |
| 1 | Remaining Minor Issues 1. Inconsistency in number of themes The Abstract states: "Seven themes..." However, Table 4 presents: • six barrier themes • at line 37, 38, it is mentioned 8 themes • two opportunity themes This indeed totals eight themes, but the wording throughout the manuscript occasionally refers to "seven themes". Please ensure consistency throughout all sections. | Thank you for this valid observation. All sections of the manuscript have been modified to ensure consistency and to accurately reflect that eight broad themes were identified in the study. Of the eight themes, **six** were barriers (1. unavailability of mental healthcare services integrated with HIV services, 2. lack of training for healthcare workers on mental healthcare, 3. lack of awareness/limited mental health literacy, 4. stigmatization, 5. long clinic waiting time and 6. transportation costs/productivity losses) and **two** were opportunities (1. existing support groups and 2. economic empowerment schemes). |  |
| 2 | Minor grammatical polishing still needed A few sentences require language editing. Examples: • "with resultant increase in mental health needs" → should read "resulting in increased mental health needs". • "Women and girls bear a substantial burden of the HIV pandemic accounting..." → consider: "Women and girls bear a substantial burden of the HIV pandemic, accounting for..." • "This means that HIV is no longer a fatal and terminal disease..." could be simplified for readability. | Thank you for these recommendations.  These changes have been implemented as recommended.  The sentence has been simplified and modified to improve readability. It now reads “*With these advancements, HIV is no longer considered terminal; rather, it is a manageable condition that permits long-term survival and healthy ageing.*” | Page 2, line 22  Page 3, line 52  Page 3, lines 60 to 61 |
| 3 | Data analysis description The manuscript simultaneously mentions: • thematic analysis • content analysis • directed content analysis The authors should explicitly state that they used: "Directed qualitative content analysis informed by Levesque's framework" to avoid methodological ambiguity. | Thank you for this recommendation. This change has been implemented as recommended. | Page 12, lines 219 to 221 |
| 4 | Participant involvement statement The limitation section mentions: "some who took part in conducting the research are living with HIV..." This statement is important but somewhat unclear. The authors should clarify whether these individuals were: • co-researchers, • peer researchers, • patient representatives, or • research assistants. | Thank you for this thoughtful question regarding the precise roles of the individuals living with HIV who assisted with this study. We respectfully clarify that in line with the principles of meaningful involvement; we engaged these individuals primarily as research assistants and peer researchers. These included three of the counsellors who supported the implementation of this study (2 as research assistants and 1 as a peer researcher) and who were living with HIV, though not openly. Their roles helped provide nuanced contexts to the issues elicited from the study participants. However, this information was not provided in the previous manuscript version due to confidentiality purposes and to avoid stigma.  We have now revised the limitations section of the manuscript to clearly state the roles of these individuals as research assistants and peer researchers. | Page 28, line 556 |
