# Supplementary material for: Exploring barriers to and opportunities for improving mental healthcare access for women caregivers living with HIV in Nigeria: A qualitative study
Source: PLOS Glob Public Health. 2026 Aug 14;6(8):e0005834. doi: 10.1371/journal.pgph.0005834 (PMC13475909; doi:10.1371/journal.pgph.0005834)
Supplement: Peer review history

# Peer review: Decision Letter 0

**DOI:** 10.1371/journal.pgph.0005834.r001

**Authors:** Peter James

4 Dec 2025

PGPH-D-25-03123

Exploring barriers to and opportunities for improving mental healthcare access for women caregivers living with HIV in Nigeria: A qualitative study

PLOS Global Public Health

Dear Dr. Nwokoro,

Thank you for submitting your manuscript to PLOS Global Public Health. After careful consideration, we feel that it has merit but does not fully meet PLOS Global Public Health’s publication criteria as it currently stands. Therefore, we invite you to submit a revised version of the manuscript that addresses the points raised during the review process.

**EDITOR**  **Comment** :

The reviewers considered your interesting and relevant but it requires futher revision before it can be considered for publication. The key issues raised by **reviewer 1**  include  grammar and writing style, linking findings to context, re-ordering the Levesque's framework section, clarifying FGD language, detailing the analysis process, adding reflexivity details, adjusting theme titles (avoiding "ignorance"), and strengthening theme evidence. The Discussion and Conclusion need substantial tightening and restructuring to avoid repetition. **Reviewer 2**  suggested changes as it relates to lack of meaningful involvement of women subjects, omission of violence/PTSD links to mental health, and inappropriate language. Additional material created by women living with HIV is also suggested for comparison

I suggest you and your co authors throughly address each of the issues raised when you submit your revised manuscript.

Please submit your revised manuscript by the**27th December 2025** . If you will need more time than this to complete your revisions, please reply to this message or contact the journal office at globalpubhealth@plos.org. When you're ready to submit your revision, log on to https://www.editorialmanager.com/pgph/ and select the 'Submissions Needing Revision' folder to locate your manuscript file.

Please include the following items when submitting your revised manuscript:

- A rebuttal letter that responds to each point raised by the editor and reviewer(s). You should upload this letter as a separate file labeled 'Response to Reviewers'.
- A marked-up copy of your manuscript that highlights changes made to the original version. You should upload this as a separate file labeled 'Revised Manuscript with Track Changes'.
- An unmarked version of your revised paper without tracked changes. You should upload this as a separate file labeled 'Manuscript'.

Guidelines for resubmitting your figure files are available below the reviewer comments at the end of this letter.

We look forward to receiving your revised manuscript.

Kind regards,

Peter Bai James, PhD

Academic Editor

PLOS Global Public Health

Journal Requirements:

If the reviewer comments include a recommendation to cite specific previously published works, please review and evaluate these publications to determine whether they are relevant and should be cited. There is no requirement to cite these works unless the editor has indicated otherwise.

Additional Editor Comments (if provided):

[Note: HTML markup is below. Please do not edit.]

Reviewers' comments:

Reviewer's Responses to Questions

**Comments to the Author**

1. Does this manuscript meet PLOS Global Public Health’s publication criteria?

Reviewer #1: Yes

Reviewer #2: Partly

**********

2. Has the statistical analysis been performed appropriately and rigorously?

Reviewer #1: N/A

Reviewer #2: N/A

**********

3. Have the authors made all data underlying the findings in their manuscript fully available (please refer to the Data Availability Statement at the start of the manuscript PDF file)?

The PLOS Data policy

Reviewer #1: No

Reviewer #2: Yes

**********

4. Is the manuscript presented in an intelligible fashion and written in standard English?

Reviewer #1: Yes

Reviewer #2: Yes

**********

Reviewer #1: I think this is an interesting paper, however it needs some work before it is worthy of publication. I have attached some detailed comments - if responded to I believe it will make the paper much stronger.

Reviewer #2: Thank you for all your work on this. Overall, I have found it an interesting and important read.

I suggest that you could strengthen the article by either amending the article where you can, or spelling out various limitations that I have indicated to you in my comments below.

1. Line 46 and throughout - please try to avoid use of ‘infections’ (see eg https://www.youtube.com/watch?v=tbx2tbFYSCU) by rearranging the sentence. Language can be very stigmatising for people with HIV. It can increase and perpetuate mental health issues. The word ‘infections’ is unnecessary here. eg ‘1.3 million more people acquired HIV globally in 2024…’

2. Line 47 *and throughout* - please avoid using letters to describe people (see above). Instead please spell these out.

3. Line 51 and throughout - please try to avoid militaristic language to describe a health condition.

4. Line 62 - please avoid this abbreviation also. How about spelling it all out the first time then say ‘hence described here as caregivers’?

5. Line 65 - I am curious to know why there is no further discussion of trauma or PTSD throughout the paper. Since an HIV diagnosis, especially for women in the context of perinatal health, can often start of increase violence against them and affect their mental health, it seems strange that this is not mentioned any further.

6. Line 67 - So what is going on in Nigeria for other people living with HIV? It would be good to clarify how services differ...

7. Line 109 - Were people openly living with HIV involved in the study design, implementation, analysis or write-up at all? If not, why not. See eg WHO 2017, section 6, especially 6.2.1. NB mental health forms an integral part of SRHR. https://iris.who.int/server/api/core/bitstreams/611f9d21-8170-495c-89b2-903886a83aee/content

8. Line 122 - I am curious to know why you used this narrow definition of caregiving? Why did you decide, for instance, not to include women living with HIV who are providing care to other family members, including their (sick) partners? See for example, a report from COWHLA in Malawi, which describes the mental health issues faced by women living with HIV who were caring for sick husbands who were in denial about their own HIV status and were refusing to let the women attend clinic to access tx themselves; or who were just abandoned them (https://cowlha.org/cowlhas-impact-through-the-stepping-stones-program-challenging-gender-norms-and-achieving-srhr-for-women-through-the-hiv-response/).

9. Line 128 - what was the gender and age breakdown of these key informants? And were any of them living openly with HIV?

10. Line 137 - were any of the researchers living openly with HIV? If not, why not? (see comment above about WHO Guideline 2017).

11. Line 141 - The experts in their own lived experiences are surely the women living with HIV themselves? Were they consulted? If not, please explain why not?

12. Line 151 - What were the education levels reached of all those interviewed? Was English a barrier to anyone taking part?

13. Line 199 - FGD participants - please provide education level (and, if relevant, level of English). Would also be good to know if they had partners at home or not.

14. Line 199 - KII participants - please provide gender and age breakdown by job title/level. And if any are openly living with HIV.

15. Line 207 - ‘Ignorance’ is a loaded word - suggest instead 'lack of awareness' or 'limited awareness’.

16. Line 207 - ‘low mental health literacy’ - I suggest instead ‘limited mental health literacy’.

17. Line 214. I suggest ‘or’ might be a clearer word to use here, rather than ‘and’.

18. Line 215. I suggest a semi-colon after the word ‘clinic’ here, instead of a comma, for clarity.

19. Line 248 - as above. I suggest ‘Lack of awareness and limited mental health literacy’ as more appropriate words, to avoid stigma.

20. Line 310-311 - some have described themselves as civil or public servants?

21. You may wish to look at and refer to the following articles also, to enhance this section. These have all be created by and/or with women living with HIV: eg Orza et al 2015 b. https://onlinelibrary.wiley.com/doi/full/10.7448/IAS.18.6.20289; Orza et al 2015 a. https://onlinelibrary.wiley.com/doi/full/10.7448/IAS.18.6.20285; Orza et al 2017. https://www.hhrjournal.org/2017/12/04/in-womens-eyes-key-barriers-to-womens-access-to-hiv-treatment-and-a-rights-based-approach-to-their-sustained-well-being/#_edn27; Dunaway et al 2022. https://journals.sagepub.com/doi/full/10.1177/17455057221080361#table2-17455057221080361; National Standards for Peer Support for HIV UK 2022. Beres et al 2017. https://positivelyuk.org/wp-content/uploads/2022/06/national_standards_final_web.pdf; https://www.tandfonline.com/doi/full/10.1080/09540121.2017.1317324

22. Line 358 ‘ignorance/low’ - see comments above.

23. Line 358 - Was there no mention of VAWG?? I'm really surprised at this - if there wasn’t, then it would seem that working more closely with caregivers as co-researchers might have brought this out...

24. Line 382. ‘Abuse’ is an inappropriate word here. How about ‘harmful substance use' instead? This fits in with a focus on ‘harm reduction’ to support people living with HIV and others.

25. Line 389 onwards. Again, I am surprised that there was no mention of VAWG when it is so widespread among women living with HIV.

26. Line 412 - please change wording again.

27. Line 453 - please see UK National Peer Support standards, ref. above.

28. Line 458 - I suggest there is also the need to talk about the knock-on consequences of mental health of caregivers to the children in their care also - certainly the caregivers have their own intrinsic rights to support - and it’s great that you focus on this here. But there are also instrumental issues here, in terms of adverse childhood experiences and the long term health of the children… see for example https://journals.sagepub.com/doi/full/10.1177/17455057221080361#table2-17455057221080361

29. Line 458 - I suggest that you also identify the other limitations of the study - eg no mention of VAWG (which is very strange); seeming lack of inclusion of caregivers in study design, implementation or analysis? You could also turn these into suggested points for inclusion in further study…

30. Line 485 - ‘ignorance and low…’ language again. And ‘WCLIHV’…

**********

what does this mean?). If published, this will include your full peer review and any attached files.

**Do you want your identity to be public for this peer review?**  If you choose “no”, your identity will remain anonymous but your review may still be made public.

For information about this choice, including consent withdrawal, please see our Privacy Policy

Reviewer #1: No

Reviewer #2: No

**********

[NOTE: If reviewer comments were submitted as an attachment file, they will be attached to this email and accessible via the submission site. Please log into your account, locate the manuscript record, and check for the action link "View Attachments". If this link does not appear, there are no attachment files.]

Attachment Submitted filename: Reviewer comments.docx

# Author comment: Author response to Decision Letter 1

**DOI:** 10.1371/journal.pgph.0005834.r002

11 Feb 2026

Attachment Submitted filename: Response to Reviewers.docx

# Peer review: Decision Letter 1

**DOI:** 10.1371/journal.pgph.0005834.r003

**Authors:** Katherine Kokkinias

31 May 2026

PGPH-D-25-03123R1

Exploring barriers to and opportunities for improving mental healthcare access for women caregivers living with HIV in Nigeria: A qualitative study

PLOS Global Public Health

Dear Dr. Nwokoro,

Thank you for submitting your manuscript to PLOS Global Public Health. After careful consideration, we feel that it has merit but does not fully meet PLOS Global Public Health’s publication criteria as it currently stands. Therefore, we invite you to submit a revised version of the manuscript that addresses the points raised during the review process.

The manuscript has been evaluated by six additional reviewers, and their comments are available below. Please pay particular attention to comments from reviewers 2, 4,6, and 8.

The reviewers have raised a number of concerns that need attention. They request additional information on methodological aspects of the study (such as the sample size determination, number of data collectors and any software used for data analysis), inclusion of mental health provisions in the study setting, and more discussion around the gap between screening policy and practice. They have also suggested points that could use additional clarification for the readers.

Could you please revise the manuscript to carefully address the concerns raised?

Please submit your revised manuscript by Jul 28 2026 11:59PM. If you will need more time than this to complete your revisions, please reply to this message or contact the journal office at globalpubhealth@plos.org. When you're ready to submit your revision, log on to https://www.editorialmanager.com/pgph/ and select the 'Submissions Needing Revision' folder to locate your manuscript file.

Please include the following items when submitting your revised manuscript:

- A letter that responds to each point raised by the editor and reviewer(s). You should upload this letter as a separate file labeled 'Response to Reviewers'.
- A marked-up copy of your manuscript that highlights changes made to the original version. You should upload this as a separate file labeled 'Revised Manuscript with Track Changes'.
- An unmarked version of your revised paper without tracked changes. You should upload this as a separate file labeled 'Manuscript'.

Guidelines for resubmitting your figure files are available below the reviewer comments at the end of this letter.

As the corresponding author, your ORCID iD is verified in the submission system and will appear in the published article. PLOS supports the use of ORCID, and we encourage all coauthors to register for an ORCID iD and use it as well. Please encourage your coauthors to verify their ORCID iD within the submission system before final acceptance, as unverified ORCID iDs will not appear in the published article. *Only*  the individual author can complete the verification step; PLOS staff *cannot*  verify ORCID iDs on behalf of authors.

We look forward to receiving your revised manuscript.

Kind regards,

Katherine Demi Kokkinias, Ph.D.

Staff Editor

PLOS Global Public Health

Journal Requirements:

If the reviewer comments include a recommendation to cite specific previously published works, please review and evaluate these publications to determine whether they are relevant and should be cited. There is no requirement to cite these works unless the editor has indicated otherwise.

Additional Editor Comments (if provided):

[Note: HTML markup is below. Please do not edit.]

Reviewers' comments:

Reviewer's Responses to Questions

**Comments to the Author**

Reviewer #2: (No Response)

Reviewer #3: All comments have been addressed

Reviewer #4: All comments have been addressed

Reviewer #5: All comments have been addressed

Reviewer #6: (No Response)

Reviewer #7: All comments have been addressed

Reviewer #8: (No Response)

**********

publication criteria?

Reviewer #2: Partly

Reviewer #3: Yes

Reviewer #4: Partly

Reviewer #5: Yes

Reviewer #6: Yes

Reviewer #7: Yes

Reviewer #8: Yes

**********

3. Has the statistical analysis been performed appropriately and rigorously?

Reviewer #2: N/A

Reviewer #3: Yes

Reviewer #4: Yes

Reviewer #5: Yes

Reviewer #6: Yes

Reviewer #7: Yes

Reviewer #8: (No Response)

**********

4. Have the authors made all data underlying the findings in their manuscript fully available (please refer to the Data Availability Statement at the start of the manuscript PDF file)?

The PLOS Data policy

Reviewer #2: Yes

Reviewer #3: Yes

Reviewer #4: Yes

Reviewer #5: Yes

Reviewer #6: Yes

Reviewer #7: Yes

Reviewer #8: Yes

**********

5. Is the manuscript presented in an intelligible fashion and written in standard English?

Reviewer #2: Yes

Reviewer #3: Yes

Reviewer #4: Yes

Reviewer #5: Yes

Reviewer #6: Yes

Reviewer #7: Yes

Reviewer #8: Yes

**********

Reviewer #2: Many thanks for your responses to my suggestions. I still have a few outstanding suggestions.

ABSTRACT

line 21. Thank you for removing the word 'infection' in one place. Please check also here and the rest of the article and remove 'infection' everywhere unless it is in the references. It is unnecessary and can further stigma.

Line 26 and throughout the article. Thank you for changing this throughout. However I suggest that for better flow, once you have described the group, you could just call them 'these caregivers' in most places, just using the full phrase again, to remind the reader who they are, when you mention them for the first time in each new section of the article.

INTRODUCTION

line 69. Thank you for now mentioning the lack of mention of VAWG in the limitations section. I think you may very well be right that they don't see it as a barrier to accessing mental health care. Unfortunately, VAWG can be so normalised (especially if it's in the form of coercive control or marital rape, rather than physical violence), that it may not be recognised by those on the receiving end as a stressor. It can also be a(nother) great source of shame and women may also feel that the VAWG is justified because they have HIV - and they may also fear that their children may be taken away from them. In either situation, it can of great help to the individual concerned if the possibility of VAWG (and, in this case, its links to mental health) is raised by the practitioner, to enable the woman to recognise that VAWG is never justified - and that it is absolutely ok to talk about it - and have it stopped, and its effects recognised and mitigated. I suggest you might consider this in future research.

Line 67 (now 71). Thank you for the helpful information you shared about mental health provision for others with HIV in Nigeria. This is very informative as background context for the reader to know. Could you add it to the article please.

RESULTS

line 246. Table 2. Part 1 - FGD participants. Could this be presented as a box, with 2 columns and 2 rows (ie 4 cells for data), in order to see the no. of participants who are eg 18-24 who have been on ART for <5 years, etc? This would provide further clarity to the reader.

Table 2 part 2. KII participants. Thank you for the disaggregation by gender. Could this be presented as a box too, so that we can see the breakdown by age and years experiencing care (x and y axes), with each of these 5 boxes subdivided by gender (so 12 data boxes in total). Again this would be helpful to the reader.

DISCUSSION

line 463. ACEs affect boys and men as well as girls and women. Maybe better to state this here.

line 550. About limited inclusion. Thank you for adding this here. I would suggest that you also add something here like: 'Lastly, *although some who took part in conducting the research are living with HIV, this has not been documented here, owing to ongoing stigma and discrimination that can arise. Despite their involvement,* ....the limited inclusion... '

Line 552. about the critical role: Suggest you amend wording to eg "... to explore certain key areas facing participants. For example, fear of.... could play a critical role...

Reviewer #3: No major concerns were identified regarding research ethics and publication ethics.

One minor comment and issues related to clarity and readability, including repetition of key terms and long sentences that could be simplified.

Reviewer #4: Review Report

Title: Exploring barriers to and opportunities for improving mental healthcare access for women caregivers living with HIV in Nigeria: A qualitative study

Comments

In general, the guideline of the journal is not described. This is seen on the abstract since it lacks sub sections. It also have consistencies for example it presented accommodation as a six component of access but missed in the others sections. Again, sven themes in the discussion and five themes were presented in the methods section and why?

Specifically, the abstract is weak. This was evidenced by the weak knowledge gap and definition of HIV. The type of the study design is also not mentioned. How many FGDs were conducted? The result needs more brief presentation and the conclusion is not focused.

In the introduction section the number, severity and size as well as distribution of HIV, its complications and the initiatives and the vivid gaps and how to fill the gaps were not presented. For instance, what is the magnitude of HIV in Nigeria? What type of mental disorders and is this mental disorders related to the HIV itself [unexplained neuro-psychiatric disorders] or due to giving care? Some of the sentences lack references, why E.g. line 85-90? The paragraphs are weakly constructed and linked. Who are these vulnerable groups? What are the packages of this mental health services?

In the methods section, what is the type of qualitative study design you have employed? Why FGDs? Why KIIs? What other parameters have you used in addition to “…in a secure and convenient location within the study sites” line 169. Were the interviews recorded? Most importantly, the quality and experience of the data collectors and how many data collectors were involved in the FGDs? Moreover, the trustworthiness of the data is NOT PRESENTED. WHY? Psycho-therapy is part of ART and why you focus on mental health and what is their difference? It didnt seem you have used soft-ware for the analysis?

In the result section, the whole section needs rewriting despite its coherence. This was manifested by lack of presenting both the negative and positive findings and its detail. For example lack of training of mental health integration with ART and why is that? Similarly, the approachability is for example given less detail presentation. IN addition, the identifier [E.g. age] of the study participants and the selection of appropriate and illustrative quote is doubtable. Some of the findings are questionable. For example, long clinic waiting time if there is no service how do they wait for a longer period of time? Why you fail to present the figure that contain the themes, categories, codes and quotations. On to top of that, if you have you used framework that contain five components why you presented seven components among which two is out of the framework?

Likewise, the discussion is relatively good. It fails to firmly present the main findings. It was poorly organized. This was evidenced by too long paragraphs. Avoid discrimination [line 435] which is out of the study objective. Similarly, it is not well organized. E.g. the opportunities were presented before the barriers. Furthermore, summarize well and present shortly. Explain well, justify it and use appropriate up to date references with important implications. Write the conclusion and the recommendation in consistent with the recommendation. Check some of the references. E.g. in reference Information sheet should be changed to fact sheet.

Regards.

Reviewer #5: I thoroughly examined the updated manuscript and discovered that the authors had successfully incorporated the recommendations from the earlier review. It does not require further changes.

Reviewer #6: REVIEWER COMMENTS

Title: Exploring barriers to and opportunities for improving mental healthcare access for women caregivers living with HIV in Nigeria: A qualitative study.

Thank you for the opportunity to review the above manuscript that addresses a critical and often overlooked intersection in global health: the mental health needs of women caregivers living with HIV (WCLHIV) in Nigeria. The study is timely, well-structured, and employs a robust qualitative methodology—incorporating 4 focus group discussions and 12 key informant interviews—to examine the systemic and individual barriers to mental healthcare for women caregivers living with HIV (WCLHIV) in Enugu State, Nigeria. Using Levesque’s conceptual framework, the authors identify seven key themes that hinder or facilitate access to care.

The paper has a number of strengths identified during the review:

1) The study adheres to the COREQ (Consolidated Criteria for Reporting Qualitative Research) checklist, which is essential for high-quality qualitative reporting.

2) The sampling strategy is appropriate for a qualitative design, and the inclusion of both service providers and service users ensures a balanced perspective.

3) The alignment of themes (Approachability, Acceptability, Availability, Affordability, and Appropriateness) with the population-level corollaries is a major strength. It moves the discussion beyond "lack of resources" to "complex barriers of engagement."

4) The identification of "productivity losses" and "transportation costs" as primary barriers is highly relevant for a Global Public Health audience.

5) The "hidden" opportunity found in Economic Empowerment schemes (Village Savings and Loan Associations) is a standout finding. It suggests that mental health interventions in LMICs should perhaps be integrated with livelihood support( there could be some studies already documented)

Comments to improve the paper:

1) The authors should briefly clarify if any saturation point was formally identified during the content analysis process to further justify the sample size

2) The authors mention that Nigerian national guidelines recommend routine mental health screening, yet it is rarely done. The discussion should more aggressively address why the gap exists between policy and practice, specifically regarding the Task-Shifting and Task-Sharing (TSTS) policy in Nigeria.

3) In the limitations section, the authors candidly note that Violence Against Women and Girls (VAWG) was not deeply explored due to the study design. Given the known link between HIV, caregiving stress, and domestic violence, the authors should suggest this as a primary avenue for future longitudinal research

4) The study notes the lack of a "two-way referral system” on page 15, line 264.The authors should provide a specific recommendation on how digital health or "referral trackers" could mitigate this systemic failure.

Reviewer #7: I have carefully reviewed your manuscript and found it to be a well-structured and meaningful contribution to the field of global public health. The study addresses an important and timely issue, and the overall quality of the work is commendable.

The manuscript is clearly written, with a logical flow of information from introduction to conclusion. The objectives are well defined, and the methodology is appropriate and sufficiently described to allow reproducibility. The results are presented in a coherent manner, supported by relevant data, and the discussion effectively interprets the findings in the context of existing literature.

I particularly appreciate:

The relevance of the research topic to current global health challenges

The sound methodological approach

The clarity in data presentation and analysis

The effort to contextualize findings within regional and global perspectives

The conclusions are justified by the data and provide useful insights for policy, practice, and future research.While the manuscript is strong overall, a few minor points may further improve clarity and impact:

Consider refining some sentences for improved readability and conciseness. Ensure consistency in formatting (e.g., units, abbreviations, referencing style). A brief elaboration on limitations would strengthen the discussion section.

Reviewer #8: 1. For the fact that the study focuses on women caregivers living with HIV, the use of the expression "people living with HIV" is not inconsistent. It is suggested that "women caregivers living with HIV" for consistency

2. There was no mention of saturation with the purposive sampling used. There should be a statement that indicated that the collection continued until thematic saturation was reached

3. Specify in the methodology section what the participants were caring for, may be ageing parents or children (or both)

4. Long clinic waiting time was quoted as a barrier, but no mention of the average wait time. If available, add quantitative context. For example, you may present the result as: "Patients reported waiting 2-4 hours on average."

**********

what does this mean?). If published, this will include your full peer review and any attached files.

**Do you want your identity to be public for this peer review?**  If you choose “no”, your identity will remain anonymous but your review may still be made public.

For information about this choice, including consent withdrawal, please see our Privacy Policy

Reviewer #2: No

Reviewer #3: **Yes:**  Mohammed Abumughessib

Reviewer #4: No

Reviewer #5: **Yes:**  Gyanesh Kumar Tiwari

Reviewer #6: No

Reviewer #7: No

Reviewer #8: **Yes:**  Taiwo Abiona

**********

[NOTE: If reviewer comments were submitted as an attachment file, they will be attached to this email and accessible via the submission site. Please log into your account, locate the manuscript record, and check for the action link "View Attachments". If this link does not appear, there are no attachment files.]

# Author comment: Author response to Decision Letter 2

**DOI:** 10.1371/journal.pgph.0005834.r004

27 Jun 2026

Attachment Submitted filename: Response_to_Reviewers_auresp_2.docx

# Peer review: Decision Letter 2

**DOI:** 10.1371/journal.pgph.0005834.r005

**Authors:** Andrés Henao-Martínez

14 Jul 2026

PGPH-D-25-03123R2

Exploring barriers to and opportunities for improving mental healthcare access for women caregivers living with HIV in Nigeria: A qualitative study

PLOS Global Public Health

Dear Dr. Nwokoro,

Thank you for submitting your manuscript to PLOS Global Public Health. After careful consideration, we feel that it has merit but does not fully meet PLOS Global Public Health’s publication criteria as it currently stands. Therefore, we invite you to submit a revised version of the manuscript that addresses the points raised during the review process.

Please submit your revised manuscript by Aug 13 2026 11:59PM. If you will need more time than this to complete your revisions, please reply to this message or contact the journal office at globalpubhealth@plos.org. When you're ready to submit your revision, log on to https://www.editorialmanager.com/pgph/ and select the 'Submissions Needing Revision' folder to locate your manuscript file.

Please include the following items when submitting your revised manuscript:

- A letter that responds to each point raised by the editor and reviewer(s). You should upload this letter as a separate file labeled 'Response to Reviewers'.
- A marked-up copy of your manuscript that highlights changes made to the original version. You should upload this as a separate file labeled 'Revised Manuscript with Track Changes'.
- An unmarked version of your revised paper without tracked changes. You should upload this as a separate file labeled 'Manuscript'.

Guidelines for resubmitting your figure files are available below the reviewer comments at the end of this letter.

As the corresponding author, your ORCID iD is verified in the submission system and will appear in the published article. PLOS supports the use of ORCID, and we encourage all coauthors to register for an ORCID iD and use it as well. Please encourage your coauthors to verify their ORCID iD within the submission system before final acceptance, as unverified ORCID iDs will not appear in the published article. *Only*  the individual author can complete the verification step; PLOS staff *cannot*  verify ORCID iDs on behalf of authors.

We look forward to receiving your revised manuscript.

Kind regards,

Andrés F. Henao-Martínez, M.D.

Academic Editor

PLOS Global Public Health

Journal Requirements:

If the reviewer comments include a recommendation to cite specific previously published works, please review and evaluate these publications to determine whether they are relevant and should be cited. There is no requirement to cite these works unless the editor has indicated otherwise.

Please review your reference list to ensure that it is complete and correct. If you have cited papers that have been retracted, please include the rationale for doing so in the manuscript text, or remove these references and replace them with relevant current references. Any changes to the reference list should be mentioned in the rebuttal letter that accompanies your revised manuscript. If you need to cite a retracted article, indicate the article’s retracted status in the References list and also include a citation and full reference for the retraction notice.

Additional Editor Comments (if provided):

[Note: HTML markup is below. Please do not edit.]

Reviewers' comments:

Reviewer's Responses to Questions

**Comments to the Author**

Reviewer #3: All comments have been addressed

Reviewer #4: All comments have been addressed

Reviewer #5: All comments have been addressed

Reviewer #7: All comments have been addressed

Reviewer #8: All comments have been addressed

**********

publication criteria?

Reviewer #3: Yes

Reviewer #4: Partly

Reviewer #5: Yes

Reviewer #7: No

Reviewer #8: Yes

**********

3. Has the statistical analysis been performed appropriately and rigorously?

Reviewer #3: Yes

Reviewer #4: N/A

Reviewer #5: Yes

Reviewer #7: Yes

Reviewer #8: Yes

**********

4. Have the authors made all data underlying the findings in their manuscript fully available (please refer to the Data Availability Statement at the start of the manuscript PDF file)?

The PLOS Data policy

Reviewer #3: Yes

Reviewer #4: Yes

Reviewer #5: Yes

Reviewer #7: Yes

Reviewer #8: Yes

**********

5. Is the manuscript presented in an intelligible fashion and written in standard English?

Reviewer #3: Yes

Reviewer #4: Yes

Reviewer #5: Yes

Reviewer #7: Yes

Reviewer #8: Yes

**********

Reviewer #3: (No Response)

Reviewer #4: We thank the authors for addressing the comments given on the previous versions. The abstract didn’t follow the guideline and format of the journal.For example the sub sections are missing and the background is wide.The introduction fails to completely present the scientific contents of it.The efforts made by the government and its partnersa in the continum of HIV care services and its gap were not well described. Try to summerize it well. Try to refrence it accordingly and entertain the gap with appropriate problem statement. The methods section is not comphrensive. E.g. Where is the type of the data collection tool and where the data was collected where is the trustworthiness of the collected data.When and how was the analysis initiated. The result is good and enrich to be more accurate,brief and clear. For instance the quality of the quotations are poor.Try to be in line with the framework and the opportunities are few why? The discussion should be accurately referenced, explained and argued. Most importantly, drawing of implications and references as well as smooth flow of the ideas is missed. The strength and limitations should be part of the study itself not from else others.

Regards,

Reviewer #5: Well done, authors. I thoroughly examined the revised manuscript and found that the authors had successfully incorporated the recommendations from the earlier review. It does not require further changes.

Reviewer #7: Remaining Minor Issues

1. Inconsistency in number of themes

The Abstract states:

"Seven themes..."

However, Table 4 presents:

• six barrier themes

• at line 37, 38, it is mentioned 8 themes

• two opportunity themes

This indeed totals eight themes, but the wording throughout the manuscript occasionally refers to "seven themes". Please ensure consistency throughout all sections.

2. Minor grammatical polishing still needed

A few sentences require language editing.

Examples:

• "with resultant increase in mental health needs" → should read "resulting in increased mental health needs".

• "Women and girls bear a substantial burden of the HIV pandemic accounting..." → consider:

"Women and girls bear a substantial burden of the HIV pandemic, accounting for..."

• "This means that HIV is no longer a fatal and terminal disease..." could be simplified for readability.

3. Data analysis description

The manuscript simultaneously mentions:

• thematic analysis

• content analysis

• directed content analysis

The authors should explicitly state that they used:

"Directed qualitative content analysis informed by Levesque's framework"

to avoid methodological ambiguity.

4. Participant involvement statement

The limitation section mentions:

"some who took part in conducting the research are living with HIV..."

This statement is important but somewhat unclear. The authors should clarify whether these individuals were:

• co-researchers,

• peer researchers,

• patient representatives, or

• research assistants.

Reviewer #8: The authors have addressed all the suggestions in my comments

**********

what does this mean?). If published, this will include your full peer review and any attached files.

**Do you want your identity to be public for this peer review?**  If you choose “no”, your identity will remain anonymous but your review may still be made public.

For information about this choice, including consent withdrawal, please see our Privacy Policy

Reviewer #3: **Yes:**  Mohammed Abu Mughaiseeb

Reviewer #4: No

Reviewer #5: **Yes:**  Gyanesh Kumar Tiwari

Reviewer #7: **Yes:**  Mohammed Abu Sayeed

Reviewer #8: **Yes:**  Taiwo Abiona

**********

[NOTE: If reviewer comments were submitted as an attachment file, they will be attached to this email and accessible via the submission site. Please log into your account, locate the manuscript record, and check for the action link "View Attachments". If this link does not appear, there are no attachment files.]

Attachment Submitted filename: Peer Review Report2.docx

# Author comment: Author response to Decision Letter 3

**DOI:** 10.1371/journal.pgph.0005834.r006

24 Jul 2026

Attachment Submitted filename: Response_to_Reviewers_auresp_3.docx

# Editor report: Decision Letter 3

**DOI:** 10.1371/journal.pgph.0005834.r007

**Authors:** Andrés Henao-Martínez

28 Jul 2026

Exploring barriers to and opportunities for improving mental healthcare access for women caregivers living with HIV in Nigeria: A qualitative study

PGPH-D-25-03123R3

Dear Dr. Nwokoro,

We are pleased to inform you that your manuscript 'Exploring barriers to and opportunities for improving mental healthcare access for women caregivers living with HIV in Nigeria: A qualitative study' has been provisionally accepted for publication in PLOS Global Public Health.

Before your manuscript can be formally accepted you will need to complete some formatting changes, which you will receive in a follow up email. A member of our team will be in touch with a set of requests.

Please note that your manuscript will not be scheduled for publication until you have made the required changes, so a swift response is appreciated.

IMPORTANT: The editorial review process is now complete. PLOS will only permit corrections to spelling, formatting or significant scientific errors from this point onwards. Requests for major changes, or any which affect the scientific understanding of your work, will cause delays to the publication date of your manuscript.

If your institution or institutions have a press office, please notify them about your upcoming paper to help maximize its impact. If they'll be preparing press materials, please inform our press team as soon as possible -- no later than 48 hours after receiving the formal acceptance. Your manuscript will remain under strict press embargo until 2 pm Eastern Time on the date of publication. For more information, please contact globalpubhealth@plos.org.

Thank you again for supporting Open Access publishing; we are looking forward to publishing your work in PLOS Global Public Health.

Best regards,

Andrés F. Henao-Martínez, M.D.

Academic Editor

PLOS Global Public Health

***********************************************************

Reviewer Comments (if any, and for reference):
